# Elucidation of Toxicity Pathways in Lung Epithelial Cells Induced by Silicon Dioxide Nanoparticles

**DOI:** 10.1371/journal.pone.0072363

**Published:** 2013-09-04

**Authors:** Odu Okoturo-Evans, Agnieszka Dybowska, Eugenia Valsami-Jones, John Cupitt, Magdalena Gierula, Alan R. Boobis, Robert J. Edwards

**Affiliations:** 1 Division of Experimental Medicine, Department of Medicine, Imperial College London, London, United Kingdom; 2 Earth Sciences Department, Natural History Museum, London, United Kingdom; 3 Geosystems Nanoscience, School of Geography, Earth and Environmental Sciences, University of Birmingham, Birmingham, United Kingdom; Universidad de Castilla-La Mancha, Spain

## Abstract

A study into the effects of amorphous nano-SiO_2_ particles on A549 lung epithelial cells was undertaken using proteomics to understand the interactions that occur and the biological consequences of exposure of lung to nanoparticles. Suitable conditions for treatment, where A549 cells remained viable for the exposure period, were established by following changes in cell morphology, flow cytometry, and MTT reduction. Label-free proteomics was used to estimate the relative level of proteins from their component tryptic peptides detected by mass spectrometry. It was found that A549 cells tolerated treatment with 100 µg/ml nano-SiO_2_ in the presence of 1.25% serum for at least 4 h. After this time detrimental changes in cell morphology, flow cytometry, and MTT reduction were evident. Proteomics performed after 4 h indicated changes in the expression of 47 proteins. Most of the proteins affected fell into four functional groups, indicating that the most prominent cellular changes were those that affected apoptosis regulation (*e.g.* UCP2 and calpain-12), structural reorganisation and regulation of actin cytoskeleton (*e.g.* PHACTR1), the unfolded protein response (*e.g.* HSP 90), and proteins involved in protein synthesis (*e.g.* ribosomal proteins). Treatment with just 10 µg/ml nano-SiO_2_ particles in serum-free medium resulted in a rapid deterioration of the cells and in medium containing 10% serum the cells were resistant to up to 1000 µg/ml nano-SiO_2_ particles, suggesting interaction of serum components with the nanoparticles. A variety of serum proteins were found which bound to nano-SiO_2_ particles, the most prominent of which were albumin, apolipoprotein A-I, hemoglobin, vitronectin and fibronectin. The use of a proteomics platform, with appropriately designed experimental conditions, enabled the early biological perturbations induced by nano-SiO_2_ in a model target cell system to be identified. The approach facilitates the design of more focused test systems for use in tiered evaluations of nanomaterials.

## Introduction

Engineered nanomaterials are being increasingly utilised in medical, pharmaceutical, food, energy, engineering and other industries to improve product performance [1]. The many and varied properties of nanomaterials in products from such industries result from the physical and chemical properties of their small size (<100 nm in any one dimension) [2]–[5]. Although nanotechnology provides many benefits, concerns have been raised about possible risks to human health due to exposure to nanoparticles which may occur accidentally or intentionally [6]–[9]. Nanoparticles can enter the human body by inhalation, ingestion or, in certain circumstances, through the skin [10] and once inside a biological environment the surface properties of the nanoparticles may allow direct interactions with biological macromolecules, such as nucleic acids, proteins and lipids [11], [12].

Nanomaterials have been reported to produce a variety of adverse, or potentially adverse, effects in vitro and in vivo [13]–[15]. The mechanisms for these effects remain unclear, although there is good evidence that they depend upon a number of the physicochemical characteristics of the nanomaterials, including size, aspect ratio, charge and surface chemistry [16], [17]. Whilst there is still some debate about how to regulate nanomaterials, for example the extent to which data requirements would differ from those for the bulk material, there is a measure of agreement that some testing in addition to that on the starting material should be undertaken [18], [19]. Currently, most testing strategies envisage a tiered approach, which would require in vivo testing for many nanomaterials before safety-in-use was adequately established [20], [21]. The development of reliable high throughput in vitro test systems (in vitro HTS), at least for use in product design and for lower tier toxicity assessments, would have obvious advantages for efficiency, resource utilization and extrapolation of findings to humans. Recently, some proposals for HTS systems to evaluate nanomaterials have been published [22]. However, these are based on known toxicity pathways, and it is not clear whether they provide sufficient coverage of toxicological space to provide a comprehensive assessment of the potential toxicity of nanomaterials [22].

High content approaches, such as toxicogenomics, which includes transciptomics, proteomics and metabonomics, have the potential to provide an unbiased evaluation of the biological response of an organism or a cell to adverse stressors. Thus, the use of a platform such as proteomics can enable all relevant toxicity pathways to be identified, and these can then be incorporated into a suitable HTS strategy [23]. In the present study we have explored this approach, in a proof-of-principle study, using silicon dioxide nanoparticles (nano-SiO_2_). These are relatively easy to prepare and inexpensive to produce and their properties have been found to be particularly suitable for biomedical applications, such as drug delivery and imaging [24], and for construction [25]. However, it has also been reported that rats exposed to nano-SiO_2_ by inhalation can develop pulmonary and cardiovascular damage such as pulmonary inflammation, myocardial ischemic damage, atrio-ventricular blockage, and increases in fibrinogen concentration and blood viscosity [26]. The mechanisms by which such damage occurs are unknown. Proteomics has been used in several recent publications to investigate cellular responses to carbon nanotubes [27]–[29]. Few other nanomaterials have been studied using proteomics. In one study, proteomics was successfully applied to assess the effects of nano-SiO_2_ on skin using a human keratinocytes cell line (HaCaT). Under the conditions used differences in the expression levels of proteins associated with oxidative stress, cytoskeleton, molecular chaperones, energy metabolism, apoptosis and tumor development were found [30].

In this present study, we chose to investigate the interaction and effects of nano-SiO_2_ on proteins expressed in human lung epithelial cell cultures in an attempt to understand the interactions that occur and the biological consequences of exposure of lung to nanoparticles. The results showed alteration in several pathways of toxicological importance and suggest that the expression levels of the proteins involved may prove useful as indicators of inhalation toxicity.

## Materials and Methods

### Physico-chemical Characterisation

Samples of a colloidal dispersion of nano-SiO_2_ with a nominal size of 22 nm (trade name Ludox TM-40, Sigma Aldrich) were characterised by X-ray diffraction (XRD), Brunauer-Emmett-Teller (BET) surface area analysis, transmission electron microscopy (TEM), fourier transform infrared (FTIR) spectroscopy and dynamic light scattering (DLS). Prior to TEM characterisation, the nano-SiO_2_ colloid suspension was diluted in water, deposited on a 300 mesh carbon coated copper grid (Agar Scientific, UK) and left to dry at room temperature overnight. Images were collected on a Hitachi 7100TEM using an accelerating voltage of 100 kV. TEM particle size was determined by measuring the diameter of 100 particles on selected TEM images using Digital Micrograph software. For DLS measurements, the nano-SiO_2_ colloidal suspension was diluted to a concentration of 40 µg/ml in MilliQ water (25°C) or cell culture medium (37°C) in the presence or absence of serum (see below for compositions), mixed by vortexing for 2 min and then transferred into disposable cuvettes for measurements using a Zetasizer Nano ZS (Malvern Instruments, UK) equipped with a He-Ne 633 nm laser. Measurement conditions were as follows: scattering angle of 173°, temperature equilibration time of 2 min, size distribution analysis algorithm (built within the DLS software) used to calculate particle size from the measured diffusion coefficient using non-negative least squares analysis. Particle surface charge was calculated from the measurement of electrophoretic mobility at 25°C in undiluted nano-SiO_2_ suspensions using approximately 1 ml of sample loaded into a disposable zeta cell and analysed using the Zetasizer instrument.

For XRD, BET surface area, and FTIR analyses the nano-SiO_2_ colloidal dispersion was dried to a powder in an oven at 60°C for 24 h. XRD was performed using an Enraf-Nonius diffractometer coupled to an INEL CPS 120 position-sensitive detector with Co K_α_ radiation. BET surface area was measured using a Micrometrics Gemini surface area analyser (Micromeritics, USA) linked to a FlowPrep 060 degasser by nitrogen adsorption using five adsorption points with P/P_0_ of 0.05–0.3. Samples were degassed overnight at 100°C for at least 12 h under flowing nitrogen gas prior to analysis. For FTIR spectroscopy the sample was mixed with potassium bromide to form a pellet prior to analysis using a PerkinElmer Spectrum 1 FTIR (PerkinElmer, UK).

### Cell Culture and Assessment of Cytotoxicity of Nano-SiO_2_

A549 human lung cancer cells (ATCC-number CCL-185) were cultured to 70–80% confluence in Dulbecco’s modified Eagle’s medium containing 100 IU/mL penicillin, 100 µg/mL streptomycin and 10% (v/v) fetal bovine serum (full growth medium) in a humidified atmosphere of air containing 5% CO_2_ at 37°C [17]. The cells, which were adherent, were removed from the flask by trypsination; the medium was removed, cells rinsed with 5 ml trypsin-EDTA solution (Sigma) and then incubated in a further 5 ml trypsin-EDTA solution for 5 min at 37°C followed by 5 ml of full growth medium. The cells were recovered by centrifugation at 1000 g for 5 min and then resuspended in medium. Cell viability was assessed by trypan blue exclusion and was typically >95%. Cells were passaged no more than 6-times.

The effects of nano-SiO_2_ on A549 cells was assessed in a series of experiments where cells were first seeded at 5×10^4^ cells/ml into a series of 6 well plates (2 ml per well) that were incubated for 3 days until the cells were 70–80% confluence. The medium was then removed, the adherent cells washed with phosphate-buffer saline (PBS), before addition of culture medium containing between 0–10% fetal bovine serum and 0–1000 µg/ml nano-SiO_2_. The cells were then incubated for up to 24 h. The effect of nano-SiO_2_ on the cells was assessed at various time points by examining their morphology by light microscopy, flow cytometry, and the so-called MTT assay. Light microscopy was performed using an inverted microscope (Olympus CKX41) and images were captured using a QICam (QImaging, Surrey, BC, Canada). Flow cytometry was performed on cells that were recovered by trypsinisation as described above. The supernatant was removed and then the cells were suspended in 188 µl PBS containing 80 µM 3-hexyl-2-[3-(3-hexyl-2(3H)benzoxazolylidene)-1-propenyl]benzoxazolium iodide (DiOC6) and 240 µM propidium iodide, incubated at 37°C for 45 min in the dark and then analysed by flow cytometry (using a FACSCalibur, BD, Oxford, UK) recording DiOC6 and propidium iodide (PI) fluorescence. The MTT assay [31] was performed in 96-well plates that were seeded with 0.1 ml 5×10^4^ cells/ml and cultured as described above before addition of fresh culture medium containing either 0% or 1.25% fetal bovine serum and between 0–100 µg/ml nano-SiO_2_. The cells were then incubated for up to 24 h after which 25 µl MTT reagent (5 mg/mL 3-[4,5-dimethylthiazol-2-yl]-2,5-diphenyltetrazolium bromide diluted in PBS) was added to the wells and the plate incubated for a further 2 h at 37°C. Then, 100 µl of a solubilisation solution (10% SDS in 50% DMF) was added to the wells and the plate and incubated overnight at 37°C. The absorbance of the purple formazan product was measured at 595 nm using a microtitre plate reader (Labsystems MultiSkan RC, VWR International).

### Proteomic Analysis of A549 Cells

A549 cells were cultured in T25 flasks until 80% confluent and then following treatment with nano-SiO_2_ (100 µg/ml) or vehicle control, the medium was removed, the cells were washed 3-times with 5 ml PBS before being dissolved in 0.1 ml 9 M urea/2% CHAPS. The protein content was determined using the bichinconic acid method (Perbio Science, UK) [32]. Each protein sample was denatured in lithium dodecyl sulphate buffer, reduced with 5 mM dithiothreitol and alkylated with 25 mM iodoacetamide for 20 min in the dark. Protein (10 µg) from each sample was separated by 1D SDS-PAGE using a 10% polyacrylamide gel and stained with InstantBlue (Novexin, UK). The gel lanes for the control and treated samples were sliced into 12 regions based on MW markers (PageRuler Prestained Protein Ladder, Geneflow) and the distribution of protein bands in the samples. The slices were decolourised, dried and digested with trypsin as previously described [33]. The resultant tryptic peptides were analysed by LC-tandem MS using an using an Agilent 1200 series Nanoflow LC system linked to an LTQ linear ion trap MS (Agilent Technologies UK Ltd., Berkshire, UK) as described previously [33]. Briefly peptide solution (8 µl) was injected into the Agilent LC and delivered with a flow rate of 10 ml/min onto a C18 trap-column (ProteCol, 0.3×10 mm, 300 Å; SGE Analytical Science Pty Ltd Victoria, Australia) the peptides were then delivered with a flow rate of 0.30 µl/min (from the nanoflow LC) to a C18 column (PicoFrit, 75 µm ID×10 cm ProteoPrep column, New Objective Inc., Massachussetts, USA) where they were separated by reverse phase chromatography. The mobile phase consisted of water containing 0.1% formic acid and a linear gradient of 5–50% acetonitrile containing 0.1% formic acid over 35 min. The eluent was directed into an LTQ linear ion trap MS. Peptides were ionized by positive mode electrospray using a voltage of 1.6 kV and the resulting ions were separated according to m/z values.

### Data Analysis

The raw data was imported into Progenesis software (version 2.5, Non-Linear Dynamics, Newcastle upon Tyne, UK) for comparative analysis of LC and m/z data between samples. The peptides (and hence proteins) were identified using Bioworks Browser software, version 3.3 (Thermo-Fisher Scientific) and the SEQUEST search engine against the *Homo sapiens* Refseq protein database (version no 2012). Proteins were selected if two or more peptides were identified with acceptable MS2 spectra having cross-correlation (Xcorr) values of 1.50, 2.00 and 3.00 for singly, doubly or triply charged ions, respectively [33]. Raw abundance intensities for each protein were assessed for possible differences in levels between samples from control and nano-SiO_2_ treated cells. Software was developed to aid the identification of trends in the Progenesis output. This software combined all the results for one gel divided into a series of slices (analysed by LC-MS/MS separately) into a single table of identified proteins. The program combined and used data from adjacent slices to aid the process. Protein isoforms were automatically combined, and proteins with shared peptides grouped together. Finally, the program sorted the protein groups by size and showed each group with a summary of the detected abundance of each peptide. When reduced to this form the task of identifying biologically interesting proteins which varied on treatment was greatly simplified. The software is written in the Ruby programming language and may be freely downloaded from github (https://github.com/jcupitt/protsift).

### Analysis of Proteins that Adhere to Nano-SiO_2_

Nano-SiO_2_ particles (100 µg/ml) were incubated in 5 ml culture medium supplemented with 1.25% serum for 4 h at 37°C. The suspension was then centrifuged at 5700 g for 10 min and the pellet re-suspended in 1 ml PBS, transferred to an Eppendorf microcentrifuge tube (ca 1.5 ml) and centrifuged at 10,000×g for 5 min. The supernatant was removed and the pellet re-suspended again in 1 ml PBS and centrifuged as before. This washing process was repeated a further 3-times. Finally, the pellet was re-suspended in 0.1 ml 9 M urea/2% CHAPS by vortex mixing for 1 min before centrifugation as before to remove any insoluble material. The supernatant was collected, the protein content was determined using the BCA method and 5 µg of protein was loaded onto an SDS-PAGE gel in preparation for proteomics as described above. The data derived from LC-MS/MS analysis of each gel slice was analysed using SEQUEST by searching a database comprising Bos taurus and Homo sapiens proteins from Refseq release 56. In addition, entire procedure was repeated in the presence of A549 cells by applying the same nano-SiO_2_–containing suspension to a T25 flask of confluent A549 cells. After 4 h the medium was removed and processed as described above.

### Immunoblotting Analysis

A549 cell lysates were separated by SDS-PAGE as described above and the proteins were transferred onto nitrocellulose filters for immunoblot analysis [23]. Filters were blocked in PBS containing BSA (3%), and incubated with a mouse polyclonal antibody against UCP2 (ab 67241: Abcam, Cambridge, UK) diluted 1∶1000 in PBS containing 0.1% BSA for 1 h at room temperature, washed 5-times in PBS containing 0.1% tween 20 and detected with goat anti-mouse IgG (H&L)-peroxidase conjugate (ab 97040: Abcam) diluted 1∶10000 in PBS containing 0.1% tween 20 for 30 min at room temperature, washed as before then developed with Luminata Crescendo Western HRP Substrate (Millipore, Watford, UK) on Amersham Hyperfilm (GE Healthcare, Little Chalfont, UK). The relative intensities of the immunoreactive bands were determined by densitometry as described previously [23].

### Statistical Analyses

Statistical calculations (Student’s *t*-test) and data handling was performed using Excel spreadsheets and GraphPad Prism (Version 4, GraphPad Software, Inc).

## Results

### Characterisation of Nano-SiO_2_ Particles

**Figure 1:**
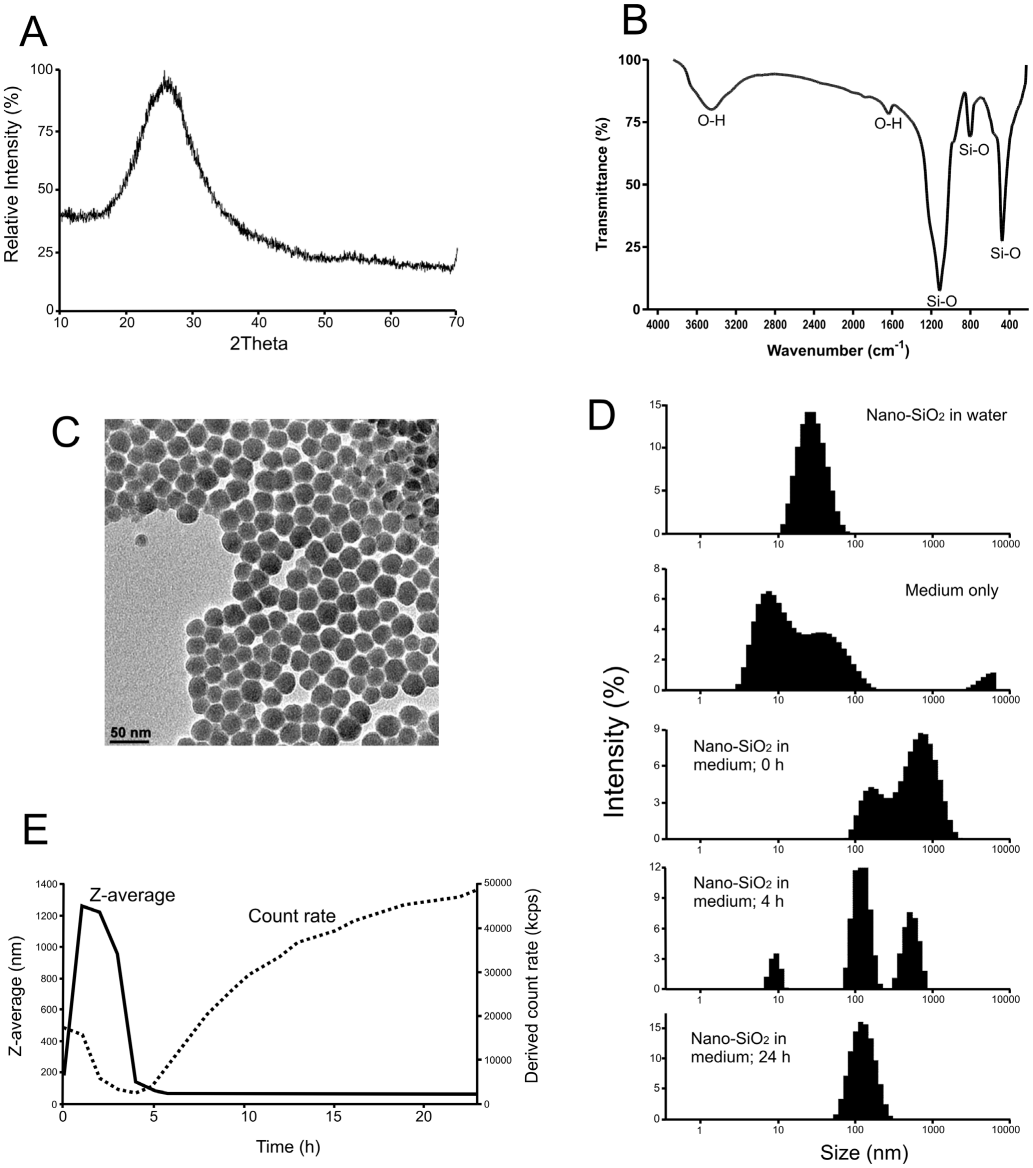
Physico-chemical characteristics of the nano-SiO_2_ preparation. (A) XRD analysis confirming the sample is amorphous silica. (B) FTIR spectrum indicating the presence of non-functionalised surfaces in the sample. (C) TEM image showing monodispersed particles with an average size of 25±2 nm. (D) DLS intensity weighed particle size distributions of nano-SiO_2_ particles in water, cell culture medium containing 1.25% serum (in the absence of nano-SiO_2_ particles), nano-SiO_2_ particles in cell culture medium containing 1.25% serum immediately after dispersion (0 h) and then after incubation at 37°C for 4 h and 24 h. (E) Changes in the average particle size (estimated from DLS) of nano-SiO_2_ dispersed in culture medium containing 1.25% serum at 37°C for up to 24 h.

Nano-SiO_2_ was acquired as colloidal suspension and, although basic characteristics of the particles were provided by the supplier, we examined the preparation further in order to ascertain the particle size distribution, surface charge, crystallinity and purity, so as to conform with current expectations of a minimum level of characterisation prior to toxicological assessment [34]. XRD analysis of the dried nano-SiO_2_ preparation showed a single peak centered at 26 2θ, which is consistent with amorphous silica, with no evidence for the presence of any crystalline phases of silica or any other impurity (Figure 1A). FTIR analysis produced a spectrum containing several absorption bands characteristic of SiO_2_ [22] (Figure 1B). The peaks at 1105 and 802 cm^–1^ correspond to antisymmetric and symmetric stretching vibrations of Si–O, respectively, the peak at 477 cm^–1^ corresponds to the bending vibration of the Si–O–Si bond, and the peaks at 3433 cm^–1^ and 1641 cm^–1^ correspond to Si–OH stretching and bending vibrations of the O–H bond of physically adsorbed water [35]. Only absorption peaks characteristic of Si-O or O-H vibrations (from absorbed water) were present in the scan indicating the surface of the SiO_2_ particles was not functionalised. TEM analysis (Figure 1C) indicated a uniform size distribution with the average particle size determined as 25±2.0 nm (n = 100) which agrees very well with the average hydrodynamic particle size as measured by DLS (sample diluted in water to 40 µg/mL, Zaver = 25.4 nm, SD = 0.1, PDI = 0.133, Figure 1D) and with the manufacturers stated particle size of 22 nm. DLS analysis of serum-containing medium (containing 1.25% FBS) revealed the presence of light-scattering particles with size distributions centred at around 10 nm and 30–40 nm. As no such peaks were found in serum-free medium, it would appear that these were due to the proteins present in serum. Addition of nano-SiO_2_ (40 µg/ml) to serum-containing medium resulted in a shift of the distribution towards larger particles sizes with two peaks observed at about 170 and 700 nm (Figure 1D) indicating that some particle agglomeration occurred immediately after dispersion (the average size of particles dispersed at the same concentration in water was 25 nm). The average particle size in the medium was observed to increase during the first 4 h following dispersion (Figure 1E), however, by 4 h (and up to 24 h after dispersion) the sample stabilised to average hydrodynamic particle size of 120 nm with a polydispersity index of 0.128 indicating that this was monodispersed suspension. The observed particle behaviour is likely due to interactions with the proteins in the medium, resulting in the formation of stable particle-protein complexes, as reported previously [36]. The BET surface area of the nano-SiO_2_ particles measured using dried samples was estimated as 112±0.5 m^2^/g. Further, the particle surface charge measured in a colloidal suspension using DLS was found to be −26.5±0.2 mV which indicates the relative electrical stability of the nanoparticles in the original colloidal dispersion.

### Effect of Nano-SiO_2_ on A549 Cells

Human lung-derived A549 cells were squamous in appearance as reported previously [37] and when cultured in vitro grew as a monolayer of cells that adhered to the culture flask. The effect of nano-SiO_2_ particles on these cells was examined in culture for up to 24 h. The condition of the cells was assessed from their morphology (light microscopy), by flow cytometry analysis and their ability to metabolise MTT.

Initially, the cells were assessed using culture medium containing 10% serum. In the absence of nano-SiO_2_ particles the cells maintained a normal phenotype throughout the incubation period and were unaffected when exposed to up to1000 µg/ml nano-SiO_2_ particles (Table 1). Thus, in the presence of 10% serum A549 cells appeared to be resistant to any cytotoxic effect of nano-SiO_2_ particles.

**Table 1:** Morphological changes in A549 cells resulting from incubation with nano-SiO_2_.

	Nano-SiO2 (µg/ml)				
time (h)	0	1	10	100	1000
**10% serum**					
0	+	+	+	+	+
1	+	+	+	+	+
2	+	+	+	+	+
4	+	+	+	+	+
6	+	+	+	+	+
8	+	+	+	+	+
24	+	+	+	+	+
**1.25% serum**					
0	+	+	+	+	+
1	+	+	+	+	−
2	+	+	+	+	−
4	+	+	+	+	−
6	+	+	+	−	−
8	+	+	+	−	−
24	+	+	+	−	−
**Serum-free**					
0	+	+	+	+	+
1	+	+	+	−	−
2	+	+	−	−	−
4	+	+	−	−	−
6	+	+	−	−	−
8	+	+	−	−	−
24	+	+	−	−	−

**Figure 2:**
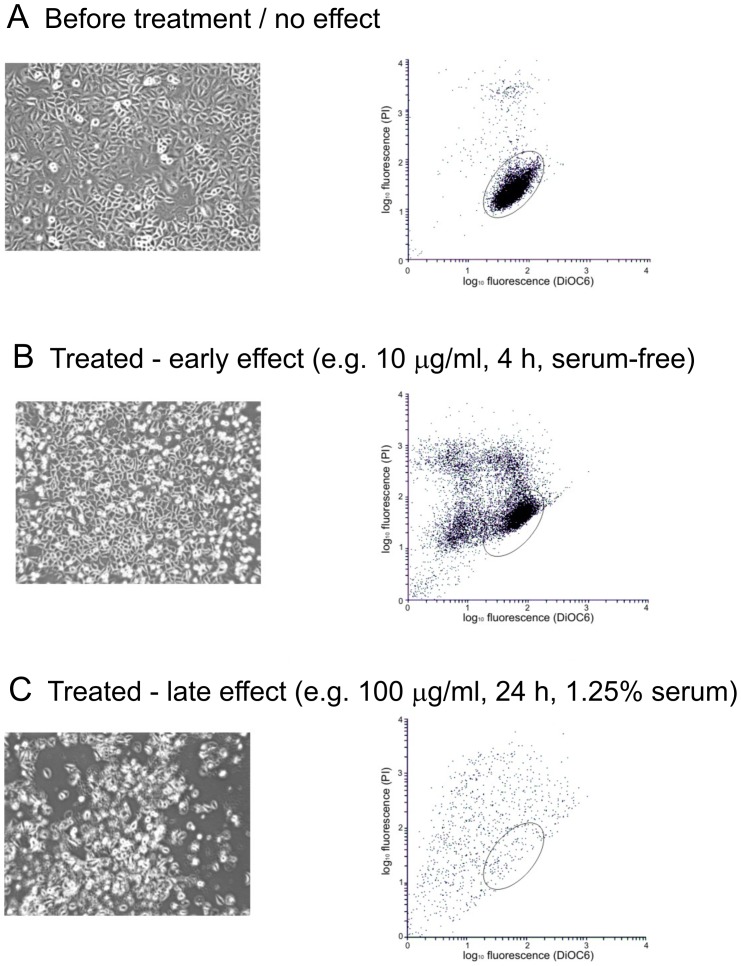
Typical morphology and flow cytometry of A549 cells. (A) Cells with a normal morphology as observed by light microscopy (magnification X250). Flow cytometry showed a relatively discrete distribution of fluorescent signals within the indicated area of interest. (B) Following treatment at effective doses a fraction of the cells took on a rounded appearance and became detached from the surface. Flow cytometry indicated a spread in the distribution of fluorescent signals outside the region of interest. (C). Later the cells became largely detached from the surface, started to clump together and lose their integrity; under such conditions very few cells were detected by flow cytometry.

A series of experiments were performed in which A549 cells were incubated with a variety of concentrations of nano-SiO_2_ for up to 24 h in medium containing different quantities of serum. The condition of the cells following treatment for different periods of time was assessed by light microscopy. In the absence of nano-SiO_2_ the cells grew normally with characteristic squamous cell morphology in the form of an adherent monolayer with very few rounded or floating cells (see figure 2). Those cultures that maintained such an appearance are indicated as ‘+’. In those cultures that responded to treatment with nano-SiO_2_ resulting in any cell abnormalities are indicated as ‘−’ (figure 2). Each set of conditions was replicated 3-times in separate cultures.

Under serum-free conditions, A549 cells maintained a normal appearance in culture for up to 24 h (Table 1). Similarly, when exposed to 1 µg/ml nano-SiO_2_ or less the cells were unaffected (Table 1). However, in the presence of 10 µg/ml nano-SiO_2_ A549 cells progressively took on a rounded appearance, shrank in size, and became detached from the culture flask (Table 1, Figure 2). This was evident in some cells after just 1 h in culture, and the fraction of cells affected increased with time, until no cells with the original morphology remained (Table 1, Figure 2). Detached cells were recovered at each time point and placed in medium containing 10% serum to neutralise the cytotoxic effect of nano-SiO_2_. However, these cells failed to adhere or proliferate indicating they were no longer viable.

The effect of nano-SiO_2_ particles was assessed further using different amounts of serum in the medium (serially diluted 2-fold from the original concentration of 10%) and from this a concentration of 1.25% serum was selected for more detailed assessment. In medium containing 1.25% serum and up to 10 µg/ml nano-SiO_2_ the cells maintained their morphology for 24 h (Figure 2b). Cells treated with 100 µg/ml nano-SiO_2_ remained unaffected for 4 h (Figure 2b) although some morphological changes were apparent by 6 h and by 24 h marked changes were evident (Table 1, Figure 2).

**Figure 3:**
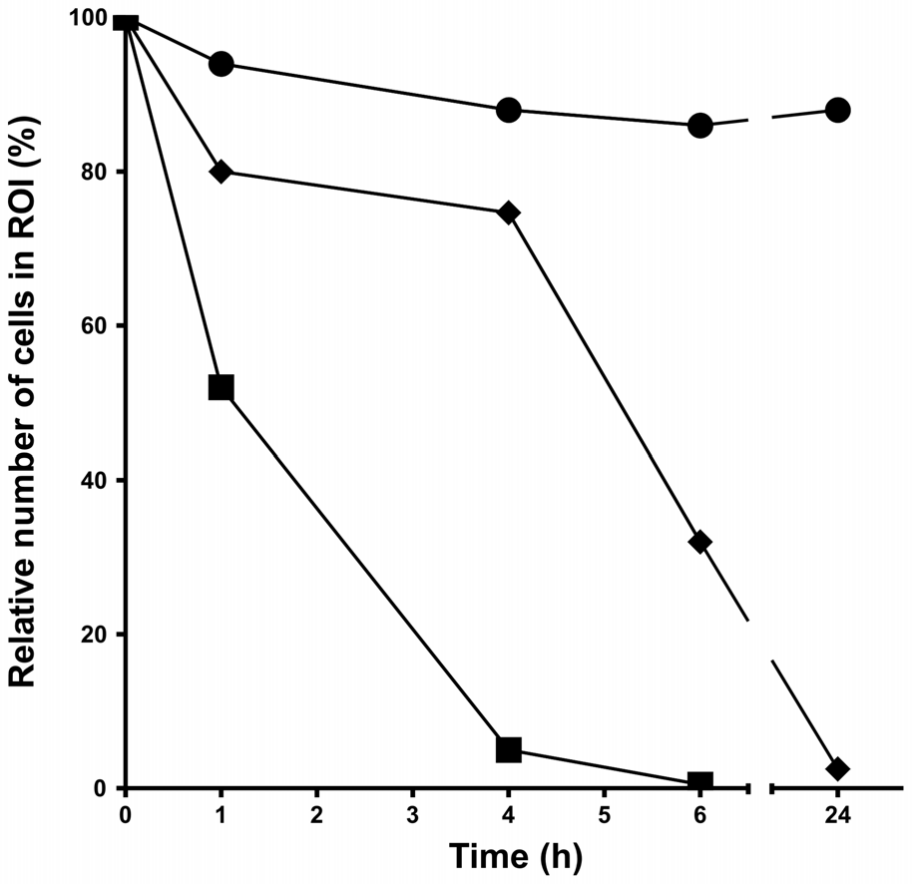
Flow cytometric analysis of A549 cells following treatment with nano-SiO_2_. A549 cells were treated for up to 24% serum (filled circles), 100 µg/ml nano-SiO_2_ in medium containing 1.25% serum (solid squares), and 10 µg/ml nano-SiO_2_ in serum-free medium (solid diamonds). At each time point the relative number of cells with fluorescent measurements in the region of interest (ROI) (see figure 2) are shown.

The condition of the cells was also examined by flow cytometry using a combination of PI and DiOC6 fluorescence measurements. A region of interest corresponding to the fluorescence characteristics of normal viable cells was defined and the number of cells in this region was estimated following treatment over time. In the absence of nano-SiO_2_ there was only a gradual and modest decrease in the number of cells in the region of interest, whether the cells were cultured in medium without serum or containing 1.25% serum (Figure 3). However, there was a rapid decrease in the number of cells in the region of interest following treatment with 10 µg/ml nano-SiO_2_ in serum-free medium. The effect was evident after just 1 h and by 4 h very few cells in the region of interest were found (Figure 3). In contrast, in cells treated with 100 µg/ml nano-SiO_2_ in medium containing 1.25% serum there was only a small decrease in the number of cells in the region of interest after 4 h, although thereafter the number of cells decreased until by 24 h very few were found (Figure 3).

**Figure 4:**
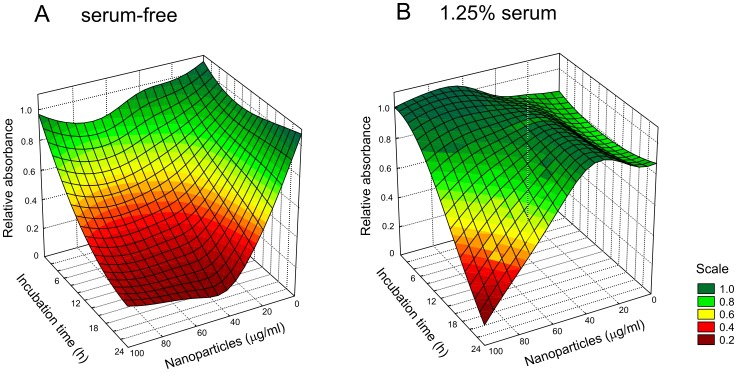
Assessment of the condition of A549 cells by MTT reduction. A549 cells were treated with up to 100 µg/ml nano-SiO_2_ for up to 24 h in the presence of (A) serum-free medium or (B) medium containing 1.25% serum. At each time point the ability of the cells to reduce MTT was determined. Each determination was performed in triplicate. The data have been plotted as surface plots that show the combined effect of treatment dose and time on MTT reduction. The colours represent different relative absorbance thresholds of MTT reduction as indicated by the scale.

MTT reduction, which is an indirect measure of mitochondrial function, was also assessed under these culture conditions. In serum-free medium at concentrations at or below 1 µg/ml nano-SiO_2_ there was no effect on MTT reduction, but above this threshold there was a rapid and substantial effect resulting in a precipitous decrease in MTT reduction (Figure 4). In contrast, in the presence of 1.25% serum, the cells were relatively tolerant and MTT reduction was affected only at a concentration of 100 µg/ml nano-SiO_2_.

Based on the changes in cell morphology, flow cytometry behaviour and MTT reduction, conditions for the proteomics studies selected were incubation for 4 h with 100 µg/ml nano-SiO_2_ in the presence of 1.25% serum.

### Proteomic Analysis of A549 Cells Treated with Nano-SiO_2_

**Figure 5:**
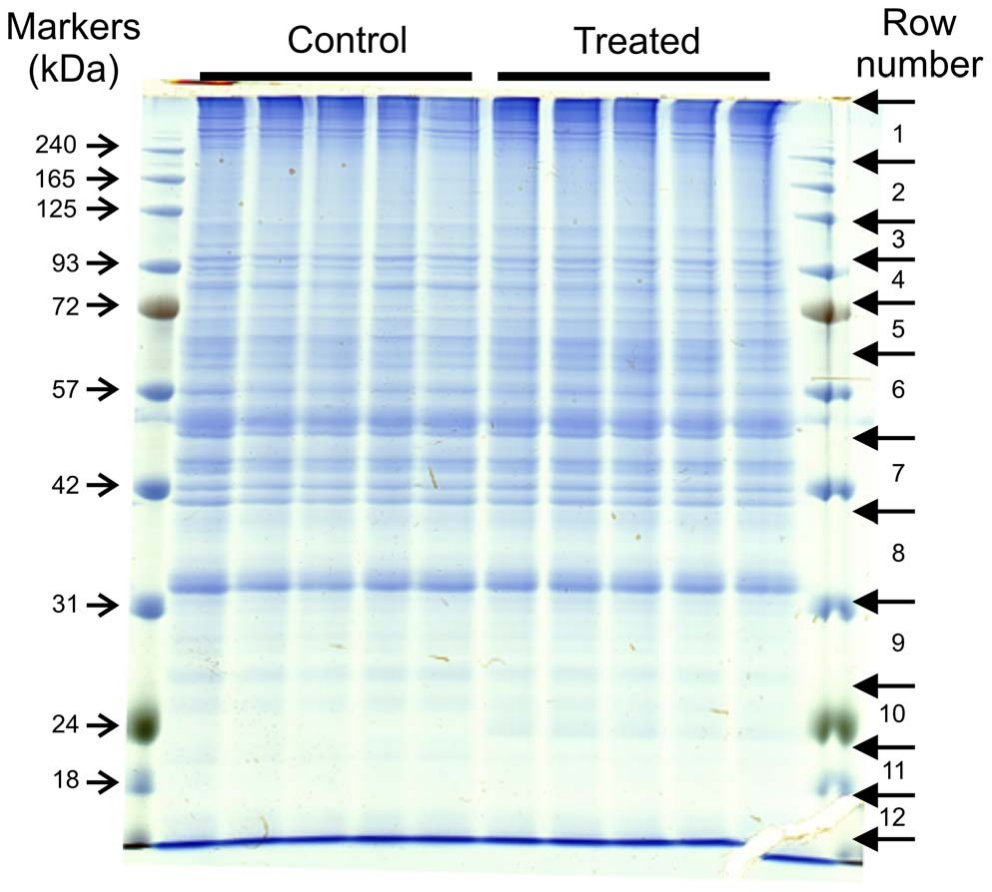
SDS-PAGE gel showing the distribution of proteins in A549 whole cell lysates. Replicate cell cultures grown in medium containing 1.25% serum were treated with vehicle control and 100 µg/ml nano-SiO_2_ A549 whole cell lysates for 4 h (n = 5). The gel was cut into 12 horizontal slices based on the migration of PageRuler Prestained Protein Ladder markers as a guide; each slice was further divided between each of the protein lanes to give 10 gel pieces for each row giving a total of 120 samples for analysis in this experiment.

**Figure 6:**
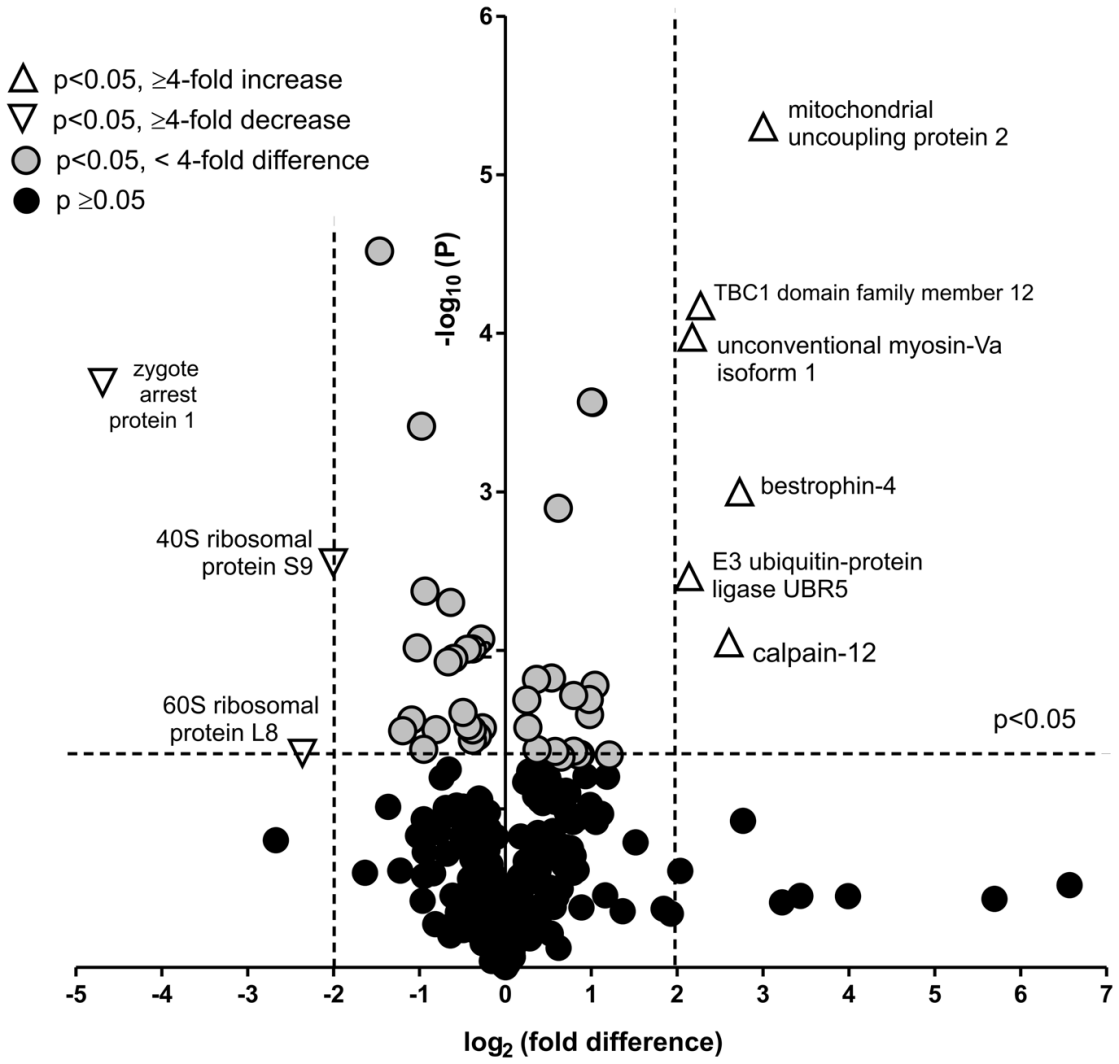
Volcano plot analysis showing the effect of nano-SiO_2_ treatment on protein expression in A549 cells. For each protein detected the relative level of protein expression following treatment is depicted on the basis of both fold change and statistical difference. The main proteins of interest are those furthest from the origin, and these are indicated as open triangles.

**Figure 7:**
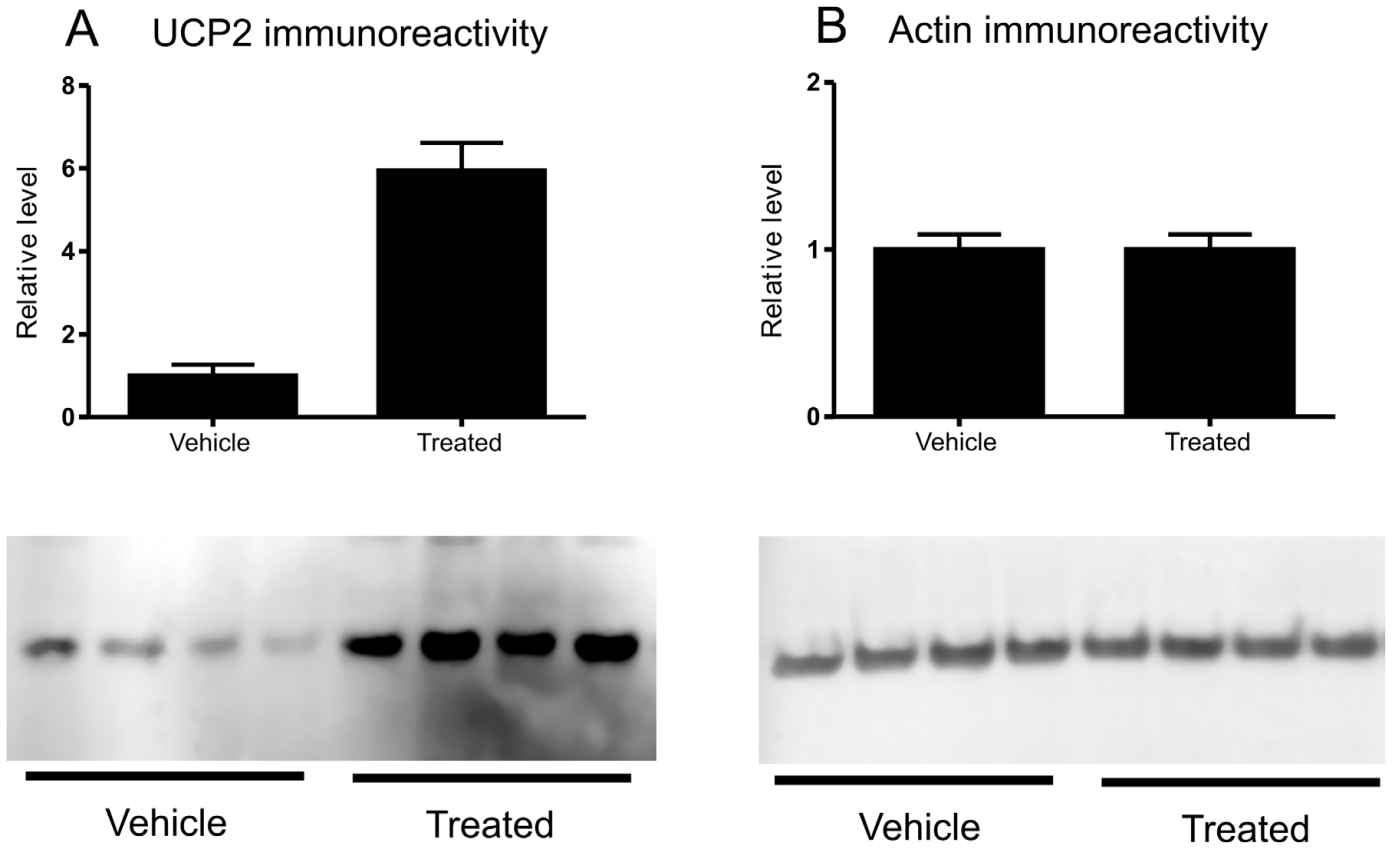
Immunoreactive levels of UCP2. Immunoblotting was performed on A549 cell homogenates following treatment with vehicle or nano-SiO_2_ and developed with antibodies against (A) UCP2 or (B) actin. The relative intensity of the immunoreactive bands was determined by densitometry as described in the Materials and Methods.

Proteomic analysis was undertaken on whole cell homogenates of A549 cells following treatment of the cells with either 100 µg/ml nano-SiO_2_ or vehicle (0.025% water) for 4 h in medium containing 1.25% serum (5 replicate incubations under each condition). Proteins in the samples were separated by 1D SDS-PAGE and stained with Instant blue (Figure 5). Each lane was then cut into a series of slices and the proteins contained in the gel pieces were digested with trypsin. The tryptic peptides produced were then analysed by LC-MS/MS. A total of 306 proteins or protein clusters were identified from LC-MS analysis of the peptides from the gel slices (Table S1). The relative expression levels of the proteins present in each set of equivalent slices of the gel was then deduced using Progenesis software. The results were then combined for all of the gel slices using custom software. The overall effect of treatment of the cells with nano-SiO_2_ on the detected proteins is illustrated in a volcano plot (Figure 6). In all, 47 proteins of interest were identified on the basis of statistical significance testing and these proteins were classified on the basis of their known function in biological processes (Table 2). Hence, proteins involved in apoptosis regulation (9 proteins), the unfolded protein response (12 proteins), structural reorganisation and regulation of actin cytoskeleton (15 proteins), and protein synthesis (6 proteins) were recognised. The levels of some of these proteins varied substantially, the greatest fold increase being in the level of mitochondrial UCP2. This was investigated further by immunoblotting, which confirmed the increase in levels of UCP2 protein, with a 6-fold change in immunoreactivity (Figure 7), similar to the 8-fold increase found by proteomics (Table 2).

**Table 2:** Differentially expressed proteins in A549 cells following treatment with nano-SiO_2_ particles.

Proteins	NCBI code	Gelregion	Folddifference	Probability
Peptide [charge]	X Corr			
**Apoptosis regulation proteins**				
Anoctamin-9	NP_001012302.2	6,7	1.8	0.045228
LDAIKMVWLQR [2+]	2.02			
FFTLQFFTHFSSLIYIAFILGRINGHPGKSTR [3+]	2.76			
Bestrophin-4	NP_695006.1	11	6.6	0.001001
FGGFSGLLLR [2+]	2.05			
YANLASVLVLR [2+]	2.19			
Calpain-12	NP_653292.2	1	6.1	0.008969
CCLRPGHYLVVPSTAHAGDEADFTLR [3+]	2.62			
TDVCQGSLGNCWFLAAAASLTLYPRLLR [3+]	2.55			
Cyclin-dependent kinase 4 inhibitor C	NP_523240.1, NP_001253.1	11	1.5	0.014932
NEVVSLMQANGAGGATNLQ [2+]	2.57			
TALQVMKLGNPEIAR [2+]	2.41			
Inositol 1,4,5-trisphosphate receptor type 3	NP_002215.2	9,10	−2.1	0.02743
EPVDPTTKGRVASFSIPGSSSR [3+]	2.6			
QRLGFVDVQNCISR [2+]	2.65			
Mitochondrial uncoupling protein 2	NP_003346.2	11	8.0	0.000005
AGGGRR [1+]	1.58			
FQAQARAGGGR [2+]	2.18			
Phosphatase and actin regulator 1	NP_112210.1, NP_001229577.1	7	−1.9	0.004217
SKSDTPYLAEAR [2+]	2.46			
RADKPWTR [2+]	2.17			
Serine/threonine-protein kinase SMG1	NP_055907.3	10,11	−1.6	0.011783
NLVLKESQR [2+]	2.13			
NSASPKHSLNGESR [2+]	2.01			
Zygote arrest protein 1	NP_783318.1	11,12	−25.8	0.000204
IDAAVQCSLGRR [2+]	2.1			
TVAVYSPLALRR [2+]	2.26			
**Unfolded protein response proteins**				
ATP-binding cassette sub-family A member 5	NP_061142.2, NP_758424.1	1,2	−1.6	0.004972
KKGENVEALR [2+]	2.19			
GIGYR [1+]	1.55			
ATP-binding cassette sub-family A member 8	NP_009099.1	6,7	1.3	0.042196
KGCFSKRKNKIATR [2+]	2.61			
KNKIATR [1+]	1.52			
Brefeldin A-inhibited guanine nucleotide-exchange protein 3	NP_065073.3	5,6	1.2	0.030546
EWLGRVGR [1+]	1.52			
SLSTAPVVQPLSIQDLVR [2+]	2			
BTB/POZ domain-containing adapter for CUL3-mediated RhoA degradation protein 1	NP_849194.1	8	2.1	0.01655
AGGAGR [1+]	1.57			
GPDPALLEATGGAAGAGGAGR [2+]	2.23			
Carboxypeptidase D isoform 1 precursor	NP_001295.2	7,8	1.7	0.043196
EVVGR [1+]	1.57			
EAAAAGLPGLAR [2+]	2.08			
Conserved oligomeric Golgi complex subunit 8	NP_115758.3	6,7	−1.2	0.008452
MNSLTLNR [1+]	2.05			
FPEAQWR [2+]	2.18			
E3 ubiquitin-protein ligase UBR5	NP_056986.2	11	4.4	0.003424
EEASLR [1+]	1.53			
MTAREEASLR [2+]	2.52			
F-box only protein 41	NP_001073879.2	10,11	−1.4	0.009824
LERLSEEVEQKIAGQVGR [2+]	2.37			
ALEKLEVDR [2+]	2.1			
Heat shock protein HSP 90-alpha isoform 1	NP_001017963.2, NP_031381.2, NP_005339.3	5,6	−1.3	0.034606
GTKVILHLKEDQTEYLEER [2+]	2.09			
GVVDSEDLPLNISR [2+]	4.22			
Ras-related protein Rab-5C isoform a	NP_958842.1, NP_001238968.1, NP_004574.2	11	−1.3	0.009775
GAQAAIVVYDITNTDTFAR [2+]	4.66			
GVDLQENNPASR [2+]	4.55			
Rho guanine nucleotide exchange factor 1 isoform 2	NP_004697.2, NP_945328.1, NP_945353.1	11,12	2.0	0.025357
LLLKSHSR [2+]	2.18			
KGGVGMPSR [2+]	2.02			
Ubiquitin-like modifier-activating enzyme 1	NP_003325.2, NP_695012.1	2,3	−2.3	0.032011
QMNPHIR [2+]	2.14			
LAGTQPLEVLEAVQR [2+]	3.72			
**Protein synthesis proteins**				
40S ribosomal protein S9	NP_001004.2	12	−4.0	0.0028
KTYVTPR [2+]	2.34			
LFEGNALLR [2+]	3.81			
40S ribosomal protein S13	NP_001008.1	12	−1.5	0.011217
LILIESR [2+]	2.18			
DSHGVAQVR [2+]	2.78			
60S ribosomal protein L8	NP_000964.1, NP_150644.1	9	−5.1	0.044527
AVVGVVAGGGR [2+]	4.1			
AVDFAER [2+]	2.53			
60S ribosomal protein L13 isoform 1	NP_150254.1, NP_000968.2, NP_001230059.1, NP_001230060.1	11	−2.8	0.00003
VATWFNQPAR [2+]	3.5			
TIGISVDPR [2+]	2.41			
GFSLEELR [2+]	2.85			
Protein PRRC2A	NP_542417.2, NP_004629.3	10,11	−2.0	0.000385
GVPSR [1+]	1.5			
QGSVTAPGGHPR [2+]	2.13			
VNSGLSSDPHFEEPGPMVR [2+]	2.52			
Protein SCAF8	NP_055707.3	7	2.0	0.000273
FPPIETR [2+]	2.27			
DVVGRPIDPR [2+]	2.11			
**Structural proteins**				
Cadherin EGF LAG seven-pass G-type receptor 3 precursor	NP_001398.2	9,10	1.6	0.047065
GLGGR [1+]	1.6			
TQDQDSQR [2+]	2.02			
CLIP-associating protein 1 isoform 2	NP_001135745.1, NP_001135746.1, NP_001193980.1, NP_056097.1	11	−2.0	0.009613
KGALLELLKITR [2+]	2.48			
ASTVSTKSVSTTGSLQRSR [2+]	2.52			
Contactin-5 isoform 1 precursor	NP_001230199.1, NP_001230200.1, NP_780775.1, NP_055176.1	7,8	2.3	0.04553
NGTEIDLESDYR [2+]	2.85			
MIRTNEAVPKTAPTNVSGR [3+]	2.88			
Diacylglycerol kinase theta	NP_001338.2	2,3	−1.9	0.041917
DARADAAPAPESDPR [2+]	2			
EGNLPSGAR [1+]	1.69			
FYVAESR [1+]	1.58			
Elongation factor 1-gamma	NP_001395.1	7	−1.3	0.031715
TFLVGER [2+]	2.69			
KLDPGSEETQTLVR [2+]	4.37			
Homeobox protein unc-4 homolog	NP_001073930.1	8	2.0	0.020386
LDLVESR [1+]	1.89			
EALALR [1+]	1.55			
Laminin subunit beta-3 precursor	NP_000219.2, NP_001017402.1, NP_001121113.1	5	1.2	0.020519
MEELRHQAR [1+]	1.84			
SFNGLLTMYQR [2+]	2			
Latent-transforming growth factor beta-binding protein 3 isoform 1 precursor	NP_001123616.1, NP_066548.2	9,10	−1.6	0.00904
MNGGQCSSR [2+]	2.07			
GLGGR [1+]	1.6			
Stomatin-like protein 2	NP_038470.1	7	−1.2	0.030743
ILEPGLNILIPVLDR [2+]	4.16			
ATVLESEGTR [2+]	2.82			
RAPR [1+]	1.71			
TBC1 domain family member 12	NP_056003.1	10,11	4.8	0.000067
DCRDLEEAR [2+]	2.11			
DLEEAR [1+]	2.26			
Tetraspanin-32	NP_620591.3	1,2	−1.4	0.029738
QELAAIQDVFLCCGKKSPFSR [3+]	2.54			
EDCLQGIR [1+]	1.89			
TRAF3-interacting protein 1 isoform 1	NP_056465.2, NP_001132962.1	9,10	−1.8	0.031608
RPPLTEKLLSKPPFR [2+]	2.25			
KPREKDKDKEKAKENGGNR [2+]	2.18			
Tropomyosin alpha/beta	NP_689476.2, NP_001036816.1, NP_001036817.1, NP_001036818.1, NP_003281.1, NP_705935.1, NP_001138632.1, NP_003280.2, NP_998839.1, NP_001018004.1, NP_001018005.1, NP_001018007.1, NP_001018008.1	9	−1.3	0.036743
EQAEAEVASLNR [2+]	4.06			
KYEEVAR [2+]	2.93			
RIQLVEEELDR [2+]	3.56			
IQLVEEELDR [2+]	3.9			
AEFAER [1+]	1.9			
KLVIIEGDLER [2+]	2.95			
Tubulin polyglutamylase TTLL4	NP_055455.3	8,9	−1.4	0.024614
QKWIVKPPASAR [2+]	2.16			
IYLFSDGLVR [2+]	2.11			
Unconventional myosin-Va isoform 1	NP_000250.3, NP_001135967.1	12	4.5	0.000106
ACGVLETIR [2+]	2.26			
LLESQLQSQKR [2+]	2.27			
**Others proteins**				
Breast carcinoma-amplified sequence 4 isoform b	NP_942094.2, NP_001010974.1, NP_060313.3	1,2	1.7	0.019224
GGGAPR [1+]	1.63			
MQRTGGGAPRPGR [2+]	2.46			
Cytosolic acyl coenzyme A thioester hydrolase isoform hBACHb	NP_863654.1	5,6	1.5	0.001268
FEEGKGR [1+]	1.81			
LVAGQGCVGPRR [2+]	2.12			
Histone H4	NP_001029249.1, NP_003529.1, NP_003530.1, NP_003531.1, NP_003532.1, NP_003533.1, NP_003534.1, NP_003535.1, NP_003536.1, NP_003537.1, NP_003486.1, NP_003539.1, NP_778224.1, NP_068803.1	12	1.8	0.045714
DNIQGITKPAIR [2+]	3.48			
ISGLIYEETR [2+]	4.27			
TLYGFGG [1+]	2.39			
Neogenin isoform 3 precursor	NP_001166095.1, NP_001166094.1, NP_002490.2	4,5	1.5	0.043386
QPLLLDDR [2+]	2.09			
SPLVR [1+]	1.7			
Uncharacterized protein C13orf30	NP_872314.1	5	2.0	0.000271
EALSYALVLRDSTKR [3+]	2.52			
EALSYALVLR [2+]	2.24			
Uncharacterized protein KIAA2013 precursor	NP_612355.1	1	1.3	0.015185
MWLQQRLKGLPGLLSSSWAR [3+]	2.59			
GEVVPLGPGVPALVANGFLALDVAANR [3+]	2.55			

Cultures of A549 cells (n = 5) were treated for 4 h with 100 µg/ml or vehicle only (n = 5) in medium containing 1.25% serum. Total cell homogenates were prepared, separated by SDS-PAGE, proteins extracted, digested with trypsin and analysed by LC-MS/MS. Relative amounts of each protein were determined from the ion intensity of the component peptides using Progenesis software and those that varied significantly are indicated by probability and fold difference (treated/untreated, except those shown as minus values which represent fold decreases, i.e. untreated/treated). The identity of the component peptides are shown along with their charge and XCorr score.

### Proteomic Analysis of Protein Corona of Nano-SiO_2_ Particles Incubated with Serum

**Figure 8:**
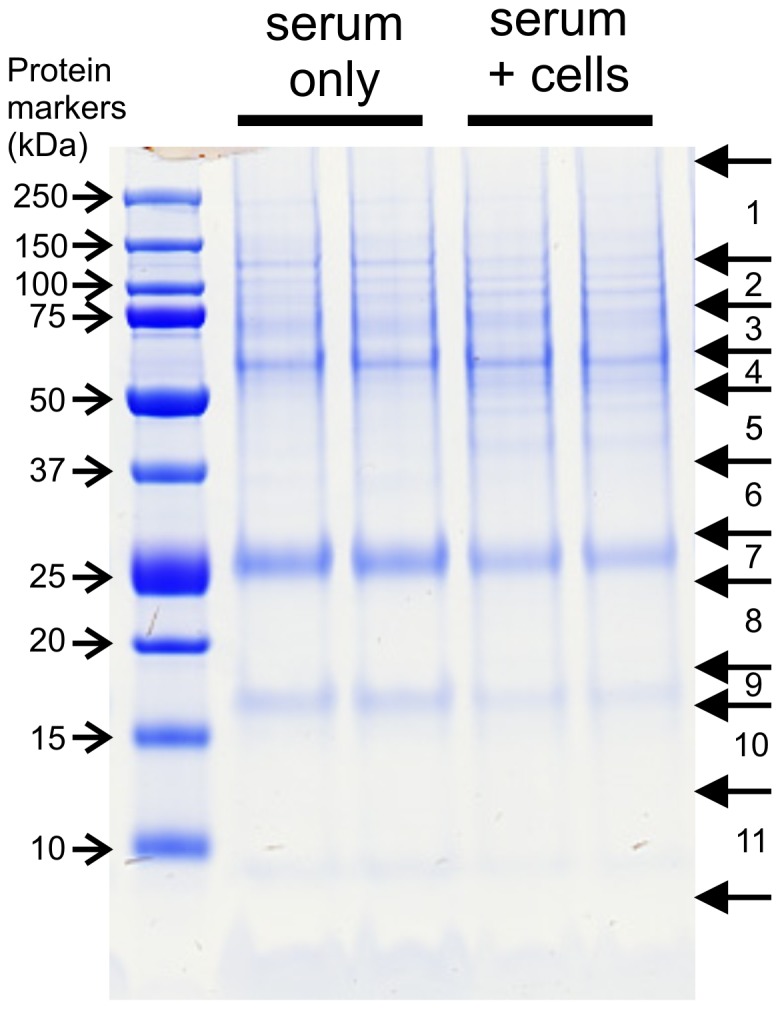
Analysis of proteins that bind to nano-SiO_2_ particles. Nano-SiO_2_ particles were incubated with 1.25% serum only or 1.25% serum and A549 cells. The nanoparticles were recovered and washed by centrifugation and then bound proteins separated by SDS-PAGE which were stained with InstantBlue. For proteomic analysis the gel was cut into 11 horizontal slices based on the migration of proteins markers and the bands in the material eluted from the nanoparticles. Each slice was further divided between each of the protein lanes for analysis by proteomics as detailed in the Materials and Methods.

Proteomic analysis was undertaken to identify proteins from serum and A549 cells that bind to nano-SiO_2_ particles. Nano-SiO_2_ particles were incubated with serum-containing medium alone or in the presence of A549 cells under the same conditions as those used above to treat the cells. SDS-PAGE analysis of washed nano-SiO_2_ particles indicated selective binding of several proteins as evident from distinct protein bands in a stained gel, albeit over a background of numerous other proteins (Figure 8). Proteomic analysis indicated the presence of 18 distinct bovine proteins, but no human proteins (Table 3). Similar results were found whether incubations were performed with bovine serum-containing medium alone or in the presence of human A549 cells. The data suggest that only proteins from the serum, and not from the cells, bound to the nanoparticles in this study. Of the protein bands observed, the one at 16 kDa (row 9) was mainly a mixture of hemoglobin subunits alpha and gamma (i.e. fetal hemoglobin), the one at 25 kDa (row 7) was mainly apolipoprotein A-I, and the one at 65 kDa (row 4) appeared to be a mixture of alpha-1-antiproteinase and alpha-2-HS-glycoprotein as well as albumin and apolipoprotein A-I. Peptides corresponding to albumin and apolipoprotein A-I were found spread across several regions of the gel. Albumin peptides were found in every row examined. This may be the result of non-specific contamination by this highly abundant serum protein. Complement C3 was found in several rows. This protein is known to undergo proteolysis to produce a variety of mature proteins. Besides these proteins, there was also clear evidence of binding of several other serum glycoproteins including the cell adhesion proteins, fibronectin and victronectin.

**Table 3:** Serum proteins that bind to nano-SiO_2_ particles.

	row											protein coverage (%)
Protein	1	2	3	4	5	6	7	8	9	10	11	
albumin [Bos taurus]	•	•	•	•	•	•	•	•	•	•	•	44
alpha-1-antiproteinase [Bos taurus]				•								21
alpha-2-HS-glycoprotein [Bos taurus]				•								32
alpha-2-macroglobulin [Bos taurus]	•											10
annexin A2 [Bos taurus]						•						29
apolipoprotein A-I [Bos taurus]				•	•	•	•	•	•	•	•	67
apolipoprotein A-II [Bos taurus]											•	40
apolipoprotein B-100 [Bos taurus]	•											10
complement C3 [Bos taurus]		•	•		•	•						16
complement factor B [Bos taurus]		•										17
fibronectin [Bos taurus]	•											12
gelsolin isoform b [Bos taurus]		•										21
hemoglobin subunit alpha [Bos taurus]									•			26
hemoglobin, gamma 2 [Bos taurus]									•	•		56
inter-alpha-trypsin inhibitor heavy chain H2 [Bos taurus]			•			•						12
peroxiredoxin-1 [Bos taurus]								•				18
pigment epithelium- derived factor [Bos taurus]					•		•					45
vitronectin [Bos taurus]			•									19
Approx. MW (kDa)	200	130	75	65	48	32	25	20	16	14	5	

Nano-SiO_2_ particles were incubated with fetal bovine serum under the same conditions as those used to treat A549 cells. The nanoparticles were recovered and bound proteins identified by proteomics as described in Materials and Methods. The table lists the main proteins identified in each row (the 4 with the highest Sf scores; minimum score of 3.0) and the rows in which they were present. Underlined symbols indicate the row where the highest protein coverage was found. The MW equivalent to the centre of each row is indicated. The experiment was performed twice and also repeated in the presence of A549 cells. A similar result was found on each occasion, i.e. all proteins identified were bovine in origin and none could be attributed to a human source (i.e. A549 cells). MS details supporting the identification of the proteins are provided in Table S2.

## Discussion

The present study was undertaken to explore the feasibility of using proteomics to identify toxicity pathways in cultured cells as a basis for the development of high throughput screens, and more focused assessments, of nanomaterials. In order to explore proof-of-principle, it was necessary to establish experimental conditions in which the cells could be studied under conditions of stress induced by nanoparticles, yet retained their viability, to ensure specificity of the response.

As others have shown [38], the effects of nanoparticles on cultured cells are critically dependent upon the incubation conditions. In the present study, it was shown that the composition of the medium used to maintain A549 cells markedly influenced the effects of nano-SiO_2_ particles on the cells. Under typical culture conditions, where the medium contained 10% serum, concentrations of nano-SiO_2_ up to 1000 µg/ml had no obvious effect on the morphology, function or viability of the cells. The protective effects of 10% serum on the cytotoxicity of nano-SiO_2_ particles have been reported previously [39]. The mechanism is not entirely clear. It was thought that aggregation was at least in part involved. However, recent studies appear to refute this, suggesting that it is the presence of proteins on the surface of the particle that reduced its toxicity [40]. In marked contrast to serum-containing medium, in serum-free medium concentrations of nano-SiO_2_ above a threshold of 1 µg/ml caused rapid and extensive cell damage, with a steep concentration-effect relationship. DLS analysis under such conditions also indicated agglomeration and sedimentation of nano-SiO_2_ particles (Figure S1). Neither 10% nor 0% serum was suitable for studying the potential effects of nano-SiO_2_ on the proteome of the cells, as in one case there was no evidence of an adverse effect and in the other cytotoxicity occurred too rapidly for any proteomic response to manifest. Consequently, the quantity of serum in the medium was varied and an amount established (1.25%) that allowed a cytotoxic effect of treatment with 100 µg/ml nano-SiO_2_ to occur over a period of time, the cells ultimately dying from apoptosis, as shown by flow cytometric analysis. Prior to this, changes in the cell proteome should become established and measureable. Indeed, under such conditions the profile of expressed proteins in A549 cells was altered. The function of many of the proteins affected appeared to be linked. Indeed, it proved possible to classify these proteins into four main groups that suggest plausible pathways of affected biological processes. These processes were those involved in the regulation of apoptosis, the unfolded protein response, structural organisation of the cell and protein synthesis.

An effect on the regulation of apoptosis was evident from the involvement of several proteins. These include cyclin-dependent kinase 4 inhibitor C and serine/threonine-protein kinase SMG1, which have previously been shown to be affected in response to DNA damage leading to activation of the intrinsic apoptotic pathway through alterations in mitochondrial membrane permeability [41]. Similarly, UCP2 is a mitochondrial membrane protein and its over-expression has previously been reported to inhibit apoptosis in A549 cells under hypoxic conditions by inhibiting reactive oxygen species production, which otherwise can damage DNA [42]. Effects on the permeability of mitochondria were also supported by the changes in the levels of anoctamin-9, inositol 1,4,5-trisphosphate receptor type 3 and bestrophin 4, which are all proteins involved in calcium regulation [43]. Changes in intracellular Ca^2+^ levels trigger the activation of calpains [44] and a member of this family (calpain 12) was also over-expressed in the nano-SiO_2_ treated cells. This protein is involved in a cascade of biochemical events that cause degradation of cytoplasmic and nuclear membranes leading to a breakdown in cellular architecture and the morphological changes associated with apoptosis (such as cellular condensation, membrane blebbing and nuclear fragmentation) [45]. In addition to alterations in proteins that could potentially affect the intrinsic (mitochondrial) apoptotic pathway depletion of phosphatase and actin regulator 1 in the nano-SiO_2_-treated cells also occurred and this response has previously been reported to trigger the extrinsic apoptotic pathway in HUVEC cells [46].

Changes suggesting that nano-SiO_2_ affected pathways involved in protein degradation were also evident. Alterations in the levels of E3 ubiquitin-protein ligase UBR5, ubiquitin-like modifier-activating enzyme 1, heat shock protein HSP 90-alpha isoform 1 and brefeldin A-inhibited guanine nucleotide-exchange protein 3 were found. These proteins are associated with Golgi membranes, secretory pathways and the ubiquitin-proteasome pathway [47]. They are all involved in the unfolded protein response (UPR), which can be activated by aberrations in endoplasmic reticulum function leading to accumulation of unfolded or misfolded proteins causing ER stress. The UPR is activated to maintain homeostasis by removing damaged proteins [48]. Like DNA damage, ER stress also activates the intrinsic apoptosis pathway, so it is possible that nano-SiO_2_ may activate apoptosis pathways through the ER stress culminating from the UPR. However, the UPR also causes an increasing demand on the Golgi network, which was evident from the changes in proteins required for normal Golgi function, such as conserved oligomeric Golgi complex subunit 8, and vesicular trafficking, such as rho guanine nucleotide exchange factor 1 isoform 2 and ras-related protein Rab-5C isoform a. These responses may also be associated with activation of autophagic cell death pathways that are characterised by the accumulation of membranous vesicles in the cytoplasm [49]. It is very likely that the UPR is associated with the decrease in protein synthesis-related pathways as seen by the reduction in several ribosomal proteins as well as changes in proteins involved in mRNA surveillance and processing, such as protein SCAF8 and protein PRRC2A.

Tropomyosin alpha-3 chain isoform 1, unconventional myosin-Va isoform 1, TBC1 domain family member 12, diacylglycerol kinase theta and TRAF3-interacting protein 1 isoform 1 are all proteins involved in transcriptional regulation and regulation of the actin cytoskeleton and these proteins were also affected by treatment with nano-SiO_2_. Hence, the cells appeared to be responding to nano-SiO_2_ by activating pathways associated with structural reorganisation which will also affect transcriptional regulation and cell cycle progression [39]. These effects activate other cell death pathways such as cornification, a form of cell death defined by considerable structural modification [50].

It is notable that these changes all occurred in cells that appeared morphologically and functionally normal at the time of analysis. Thus, the proteomic changes observed indicate an early response of the cells to treatment with nano-SiO_2_. Such alterations may represent affected pathways, including those leading to apoptosis and other mechanism of cell death [51]. The changes in PI and DiOC6 fluorescence that are observed some hours after nano-SiO_2_ exposure are consistent with alterations in nuclear membrane and mitochondrial membrane permeabilisation, as has been reported by others [52]. Further studies that define more precisely the pathways affected and their relationship to the process of cell death should be informative as to cytotoxic mechanisms of nano-SiO_2_ exposure. Proteomic studies on lung-derived BEAS-2B cells with titanium dioxide nanoparticles revealed changes in some of proteins affected here, but in general the main pathways affected differed from those identified in the present study [28]. For example, Ge et al (2011) found no change in the unfolded protein response and in the present study, evidence of oxidative stress was not prominent. Similarly, in studies on single wall carbon nanotubes in HepG2 cells, whilst there was some overlap in the functional categories of proteins identified, there was only minor overlap in the individual proteins affected [27]. The key difference between the findings of the present study and those of most previous studies concerning the toxicity of nanoparticles to cultured cells was the relative lack of evidence of oxidative stress. Whilst this will clearly depend on the nature of nanoparticle and the cell type used, previous studies on nano-SiO_2_ and/or A549 cells have reported increased oxidative stress [53]–[58]. This difference in findings may be because of the choice of experimental conditions in the present study, which enabled cellular responses to be assessed prior to loss of viability or deterioration in morphology. This emphasises the importance of study design in such investigations. Early responses are likely to be the most discriminating.

It is known that in a biological environment nano-SiO_2_ will associate with macromolecules (*e.g.* proteins and lipids) that coat the nanoparticles and form what has been termed a “corona”. Such a coating influences the uptake, toxicity, cellular response and clearance rates of nano-SiO_2_ particles [59], [60]. Analysis of the corona of A549 cells incubated with culture medium by SDS-PAGE indicated preferential binding of three main serum proteins, and these were identified as albumin, apolipoprotein A-I and hemoglobin. Interaction of nano-SiO_2_ with these proteins has been reported previously [11]. Additionally, proteomic analysis indicated the presence of fibronectin and vitronectin, which have also been reported to bind to silica [61], [62]. It is possible that the presence of such proteins on the surface of the nanoparticles were responsible for the attenuation of cytotoxicity observed, although it is not possible from the present work to eliminate the effect of other non-proteinaceous components of serum. Apolipoproteins are lipid binding proteins with detergent-like amphipathic properties that behave as water-soluble carriers in lymph and blood. A coating of apolipoprotein A-I on the surface of nano-SiO_2_ particles may impart amphipathic properties on the nanoparticles thus facilitating their transport through the lymphatic and circulatory systems and possible elimination. The presence of vitronectin and fibronectin in the corona is of interest as these proteins are involved in cell membrane stability, integrity, adhesion and wound healing and such proteins in the coating could affect the ability of nano-SiO_2_ particles to penetrate cells. It would be interesting to examine this further by identifying any intracellular proteins that bind nano-SiO_2_ particles, as this may give clues as to the mechanism of toxicity.

## Conclusions

The use of a proteomics platform, with appropriately designed experimental conditions, enabled the early biological perturbations induced by nano-SiO_2_ in a model target cell system to be identified. Similar studies, on nanoparticles with different physico-chemical characteristics should enable the toxicity pathways/key events on their interaction with cells to be mapped. This could then serve as a basis for the design of suitable HTS and more focused test systems for use in the tiered evaluation of both candidate and existing nanomaterials. The biological matrix in which nanomaterials are located has a marked effect on their biological activity. Once suitable assays have been developed, risk assessment will require evaluation of nanomaterials under realistic biological conditions, rather than in the artificial environment of cultured cells, as is often the case at present. The present study has focused on developing screening approaches for hazard identification, but a suitable risk assessment strategy will need to take into account exposure relative to toxicological potency, which will require higher tier approaches.

## Supporting Information

Figure S1 **Particle agglomeration and sedimentation in the absence of serum.** DLS determinations of the average particle size of nano-SiO_2_ dispersed in culture medium without addition of serum were distinctly different from those obtained in the presence of serum. Incubations were performed under similar conditions to those described in figure 1D and 1E, except that serum was omitted from the culture medium. The results show variation in the z-average, which is indicative of particle agglomeration, and a marked decrease in the count rate indicative of a loss of particles from the suspension and consistent with particle sedimentation.
(DOCX)

Table S1 **Proteins identified in A549 cells.** MS data of tryptic peptide mixtures were analysed by SEQUEST and the peptides were assigned to human proteins. The data was then further analysed to reveal all proteins that contain each peptide and also where assignments overlap due to proteins containing one or more common peptides (clusters). The table lists all proteins or protein clusters by descriptive name and NCBI code and the corresponding peptides that led to their assignment, along with the charge of the peptide and the cross-correlation score for the peptide. The region of gel that from which the peptides were extracted is also indicated.
(DOCX)

Table S2 **Identification of proteins bound to nano-SiO_2_ particles.** The identification of the proteins listed in Table 3 was based on MS data of tryptic peptides that was analysed by SEQUEST. For each protein the corresponding peptides found are indicated along with their charge and cross-correlation score (Xcorr). The final score (Sf) for each protein is also shown.
(DOCX)
