# Supplementary figures and images for: Elucidation of Toxicity Pathways in Lung Epithelial Cells Induced by Silicon Dioxide Nanoparticles
Source: PLoS One. 2013 Sep 4;8(9):e72363. doi: 10.1371/journal.pone.0072363 (PMC3762866; doi:10.1371/journal.pone.0072363)

**Figure S1.** **Particle agglomeration and sedimentation in the absence of serum.**


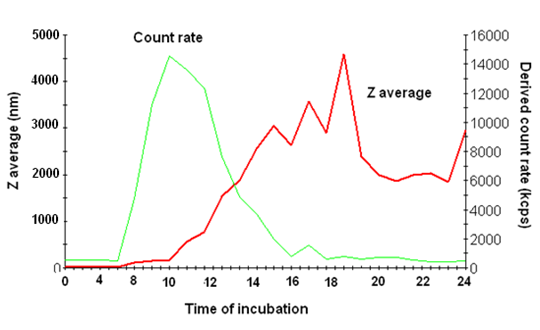

Supplement: Figure S1 — Particle agglomeration and sedimentation in the absence of serum. DLS determinations of the average particle size of nano-SiO2 dispersed in culture medium without addition of serum were distinctly different from those obtained in the presence of serum. Incubations were performed under similar conditions to those described in figure 1D and 1E, except that serum was omitted from the culture medium. The results show variation in the z-average, which is indicative of particle agglomeration, and a marked decrease in the count rate indicative of a loss of particles from the suspension and consistent with particle sedimentation. (DOCX) [file pone.0072363.s001.docx]
