# Supplementary material for: Elucidation of Toxicity Pathways in Lung Epithelial Cells Induced by Silicon Dioxide Nanoparticles
Source: PLoS One. 2013 Sep 4;8(9):e72363. doi: 10.1371/journal.pone.0072363 (PMC3762866; doi:10.1371/journal.pone.0072363)
Supplement: Table S1 — Proteins identified in A549 cells. MS data of tryptic peptide mixtures were analysed by SEQUEST and the peptides were assigned to human proteins. The data was then further analysed to reveal all proteins that contain each peptide and also where assignments overlap due to proteins containing one or more common peptides (clusters). The table lists all proteins or protein clusters by descriptive name and NCBI code and the corresponding peptides that led to their assignment, along with the charge of the peptide and the cross-correlation score for the peptide. The region of gel that from which the peptides were extracted is also indicated. (DOCX) [file pone.0072363.s002.docx]

**Table S1. Proteins identified in A549 cells.**

| cluster information |  |  |  |  |  |
| --- | --- | --- | --- | --- | --- |
| protein NCBI code | protein description |  |  |  |  |
| peptide sequence | peptide charge (+) | peptide score | gel region |  |  |
| ** cluster of 2 proteins |  |  |  |  |  |
| NP_001230183.1 | DENN domain-containing protein 5A isoform 2 | | |  |  |
| NP_056028.2 | DENN domain-containing protein 5A isoform 1 | | |  |  |
| TMGSTIR | 1 | 1.54 | 9 |  |  |
| KVLAR | 1 | 1.61 | 9 |  |  |
| NP_057336.3 | baculoviral IAP repeat-containing protein 6 | |  |  |  |
| KVLAR | 1 | 1.61 | 9 |  |  |
| YNLACHR | 1 | 1.85 | 9 |  |  |
| ** cluster of 2 proteins |  |  |  |  |  |
| NP_004597.2 | transcription initiation factor TFIID subunit 1 isoform 1 | | |  |  |
| NP_620278.1 | transcription initiation factor TFIID subunit 1 isoform 2 | | |  |  |
| RLSLKNAKQLLR | 2 | 2.02 | 5 |  |  |
| EEEEQR | 1 | 1.90 | 4 |  |  |
| NP_001165780.1 | nexilin isoform 2 |  |  |  |  |
| NP_653174.3 | nexilin isoform 1 |  |  |  |  |
| EEEEQR | 1 | 2.02 | 3 |  |  |
| KAEEEAR | 1 | 1.65 | 4 |  |  |
| ** cluster of 3 proteins |  |  |  |  |  |
| NP_057444.2 | translational activator of cytochrome c oxidase 1 | | |  |  |
| RAAAR | 1 | 1.58 | 1 |  |  |
| GCGAAPGR | 1 | 2.02 | 1 |  |  |
| NP_892023.1 | guanine nucleotide-binding protein G(olf) subunit alpha isoform 1 | | | |  |
| FQVDKVNFHMFDVGGQR | 2 | 2.39 | 1 |  |  |
| RAAAR | 1 | 1.58 | 1 |  |  |
| NP_997246.2 | nck-associated protein 5 isoform 1 | |  |  |  |
| RAAAR | 1 | 1.58 | 1 |  |  |
| DWVQCPKSQTPGSR | 2 | 2.34 | 1 |  |  |
| ** cluster of 3 proteins |  |  |  |  |  |
| NP_003487.1 | transformation/transcription domain-associated protein isoform 2 | | | |  |
| NP_001231509.1 | transformation/transcription domain-associated protein isoform 1 | | | |  |
| VLQLLR | 1 | 1.93 | 10 |  |  |
| IAALNALAACNYLPQSR | 2 | 2.27 | 10 |  |  |
| LTSVTR | 1 | 1.67 | 10 |  |  |
| KWMEVVVITHKGGQR | 2 | 2.35 | 11 |  |  |
| NP_001184188.1 | origin recognition complex subunit 3 isoform 3 | | |  |  |
| NP_036513.2 | origin recognition complex subunit 3 isoform 2 | | |  |  |
| NP_862820.1 | origin recognition complex subunit 3 isoform 1 | | |  |  |
| VLQLLR | 1 | 1.93 | 10 |  |  |
| ENVVNFIDCLVR | 2 | 2.22 | 10 |  |  |
| NP_000962.2 | 60S ribosomal protein L7 |  |  |  |  |
| VLQLLR | 1 | 1.93 | 10 |  |  |
| IALTDNALIAR | 2 | 4.25 | 10 |  |  |
| ** cluster of 6 proteins |  |  |  |  |  |
| NP_001017981.1 | RING finger protein 215 precursor | |  |  |  |
| RGGAR | 1 | 1.53 | 2 |  |  |
| GPAAR | 1 | 1.52 | 3 |  |  |
| NP_001243437.1 | carabin isoform 2 |  |  |  |  |
| AGTPAPATGPPR | 2 | 2.11 | 2 |  |  |
| GPAAR | 1 | 1.52 | 3 |  |  |
| NP_620152.1 | ankyrin repeat domain-containing protein 54 | | |  |  |
| LDDLCTR | 2 | 2.47 | 3 |  |  |
| RGGAR | 1 | 1.53 | 2 |  |  |
| NP_006550.1 | KH domain-containing, RNA-binding, signal transduction-associated protein 1 | | | | |
| RGGAR | 1 | 1.53 | 2 |  |  |
| GGGGGSR | 1 | 1.53 | 3 |  |  |
| NP_149025.1 | galactose-3-O-sulfotransferase 3 | |  |  |  |
| RGGAR | 1 | 1.53 | 2 |  |  |
| AARTWNALDAGLYDHFNATFWR | 3 | 2.52 | 2 |  |  |
| NP_078928.3 | leucine-rich repeat serine/threonine-protein kinase 1 | | |  |  |
| AYVLALCRSPSGSR | 2 | 2.45 | 1 |  |  |
| RGGAR | 1 | 1.53 | 2 |  |  |
| ** cluster of 2 proteins |  |  |  |  |  |
| NP_006752.1 | 14-3-3 protein epsilon |  |  |  |  |
| LAEQAER | 2 | 2.84 | 9 |  |  |
| YLAEFATGNDR | 2 | 3.96 | 9 |  |  |
| NP_006817.1 | 14-3-3 protein theta |  |  |  |  |
| YLAEVACGDDR | 2 | 4.18 | 10 |  |  |
| LAEQAER | 2 | 2.84 | 9 |  |  |
| EKVESELR | 2 | 2.53 | 10 |  |  |
| ** cluster of 2 proteins |  |  |  |  |  |
| NP_997193.1 | coiled-coil domain-containing protein 74B | |  |  |  |
| LAGGSADTVR | 2 | 2.34 | 11 |  |  |
| ENKDLR | 1 | 1.72 | 10 |  |  |
| NP_001004334.2 | probable G-protein coupled receptor 179 precursor | | |  |  |
| YLAEFPEALAR | 2 | 2.07 | 10 |  |  |
| ENKDLR | 1 | 1.72 | 10 |  |  |
| ** cluster of 5 proteins |  |  |  |  |  |
| NP_689955.1 | glypican-2 precursor |  |  |  |  |
| DGSGGKGGGGSAR | 2 | 2.05 | 5 |  |  |
| ARPPR | 1 | 1.52 | 5 |  |  |
| NP_055149.2 | uncharacterized protein C9orf4 |  |  |  |  |
| AGGLGGGSVPARAR | 2 | 2.01 | 5 |  |  |
| ARPPR | 1 | 1.52 | 5 |  |  |
| NP_828848.3 | cytosolic phospholipase A2 delta | |  |  |  |
| GLPFPRVEPSPQDQHQPR | 2 | 2.82 | 5 |  |  |
| ARPPR | 1 | 1.52 | 5 |  |  |
| NP_001230469.1 | differential display clone 8 isoform 2 | |  |  |  |
| NP_001230470.1 | differential display clone 8 isoform 1 | |  |  |  |
| LQSARLLGWGGGR | 2 | 2.02 | 5 |  |  |
| ARPPR | 1 | 1.52 | 5 |  |  |
| NP_068603.4 | cytosolic carboxypeptidase-like protein 5 isoform 1 | | |  |  |
| GLRTPPKSHNGLPVSCSENTLSR | 3 | 2.58 | 4 |  |  |
| ARPPR | 1 | 1.52 | 5 |  |  |
| ** cluster of 2 proteins |  |  |  |  |  |
| NP_005991.1 | tubulin alpha-4A chain |  |  |  |  |
| NLDIERPTYTNLNR | 2 | 4.01 | 6 |  |  |
| AVFVDLEPTVIDEIR | 2 | 2.66 | 7 |  |  |
| NP_006073.2 | tubulin alpha-1B chain |  |  |  |  |
| NP_116093.1 | tubulin alpha-1C chain |  |  |  |  |
| NP_006000.2 | tubulin alpha-1A chain |  |  |  |  |
| NLDIERPTYTNLNR | 2 | 4.01 | 6 |  |  |
| AVFVDLEPTVIDEVR | 2 | 5.66 | 7 |  |  |
| ** cluster of 2 proteins |  |  |  |  |  |
| NP_002565.1 | peroxiredoxin-1 |  |  |  |  |
| NP_859047.1 | peroxiredoxin-1 |  |  |  |  |
| NP_859048.1 | peroxiredoxin-1 |  |  |  |  |
| NP_001189360.1 | peroxiredoxin-1 |  |  |  |  |
| QITVNDLPVGR | 2 | 3.78 | 12 |  |  |
| SVDETLR | 2 | 2.21 | 11 |  |  |
| NP_005800.3 | peroxiredoxin-2 isoform a |  |  |  |  |
| QITVNDLPVGR | 2 | 3.78 | 12 |  |  |
| GLFIIDGKGVLR | 2 | 2.40 | 11 |  |  |
| ** cluster of 2 proteins |  |  |  |  |  |
| NP_001156002.1 | SH3 domain-containing protein 21 isoform 1 | |  |  |  |
| EVESLR | 1 | 1.97 | 10 |  |  |
| WVPARGWLR | 1 | 1.85 | 9 |  |  |
| NP_079390.3 | centrosomal protein of 290 kDa |  |  |  |  |
| KELKKETDAAEKLR | 3 | 2.71 | 10 |  |  |
| EVESLR | 1 | 1.97 | 10 |  |  |
| ** cluster of 2 proteins |  |  |  |  |  |
| NP_000250.3 | unconventional myosin-Va isoform 1 | |  |  |  |
| NP_001135967.1 | unconventional myosin-Va isoform 2 | |  |  |  |
| ACGVLETIR | 2 | 2.26 | 12 |  |  |
| LLESQLQSQKR | 2 | 2.27 | 12 |  |  |
| NP_061198.2 | unconventional myosin-Vc |  |  |  |  |
| ACGVLETIR | 2 | 2.26 | 12 |  |  |
| EKMSEITKQLLESYDIEDVR | 2 | 2.62 | 12 |  |  |
| ** cluster of 2 proteins |  |  |  |  |  |
| NP_060570.2 | EF-hand domain-containing protein 1 isoform 1 | | |  |  |
| NP_001165891.1 | EF-hand domain-containing protein 1 isoform 2 | | |  |  |
| EVHERNDGR | 2 | 2.14 | 5 |  |  |
| GKTFR | 1 | 1.62 | 5 |  |  |
| NP_919306.2 | zinc finger protein 30 isoform b |  |  |  |  |
| NLVSMGHSR | 2 | 2.38 | 5 |  |  |
| GKTFR | 1 | 1.62 | 5 |  |  |
| ** cluster of 3 proteins |  |  |  |  |  |
| NP_110394.3 | AT-hook-containing transcription factor | |  |  |  |
| QGRPLPR | 1 | 1.71 | 7 |  |  |
| DPLAR | 1 | 1.69 | 6 |  |  |
| NP_071895.3 | inactive rhomboid protein 1 |  |  |  |  |
| GWRKQKEGAAAPQPKVR | 3 | 2.52 | 5 |  |  |
| DPLAR | 1 | 1.69 | 6 |  |  |
| NP_036234.3 | arginine-glutamic acid dipeptide repeats protein isoform a | | | |  |
| NP_001036146.1 | arginine-glutamic acid dipeptide repeats protein isoform a | | | |  |
| NP_001036147.1 | arginine-glutamic acid dipeptide repeats protein isoform b | | | |  |
| DPLAR | 1 | 1.69 | 6 |  |  |
| AAKASSSAHEGR | 2 | 2.08 | 7 |  |  |
| ** cluster of 2 proteins |  |  |  |  |  |
| NP_004218.1 | cytohesin-3 |  |  |  |  |
| VVEGNHVVYR | 2 | 2.03 | 6 |  |  |
| DIERL | 1 | 1.54 | 6 |  |  |
| NP_005404.1 | homeobox protein SIX3 |  |  |  |  |
| DRAAAAKNR | 1 | 1.86 | 6 |  |  |
| DIERL | 1 | 1.54 | 6 |  |  |
| ** cluster of 5 proteins |  |  |  |  |  |
| NP_004360.2 | collagen alpha-3(VI) chain isoform 1 precursor | | |  |  |
| NP_476508.2 | collagen alpha-3(VI) chain isoform 5 precursor | | |  |  |
| RAGGSR | 1 | 1.80 | 8 |  |  |
| LVLKQ | 1 | 1.70 | 8 |  |  |
| NP_476506.3 | collagen alpha-3(VI) chain isoform 3 precursor | | |  |  |
| TLIERLVDYLDVGFDTTR | 2 | 2.90 | 7 |  |  |
| RAGGSR | 1 | 1.80 | 8 |  |  |
| NP_733936.1 | succinate-semialdehyde dehydrogenase,  mitochondrial isoform 1 precursor | | | |  |
| NP_001071.1 | succinate-semialdehyde dehydrogenase,  mitochondrial isoform 2 precursor | | | |  |
| LVLKQ | 1 | 1.70 | 8 |  |  |
| RLGSTFPGCR | 1 | 1.52 | 9 |  |  |
| NP_056009.1 | unconventional myosin-Id |  |  |  |  |
| LVLKQ | 1 | 1.70 | 8 |  |  |
| SGFILSVPGN | 1 | 1.80 | 8 |  |  |
| NP_002840.2 | receptor-type tyrosine-protein phosphatase R isoform 1 precursor | | | |  |
| NP_570897.2 | receptor-type tyrosine-protein phosphatase R isoform 2 | | |  |  |
| NP_001193944.1 | receptor-type tyrosine-protein phosphatase R isoform 3 | | |  |  |
| NP_001193945.1 | receptor-type tyrosine-protein phosphatase R isoform 4 | | |  |  |
| YKTILPNPLSR | 1 | 2.41 | 9 |  |  |
| LVLKQ | 1 | 1.70 | 8 |  |  |
| ** cluster of 3 proteins |  |  |  |  |  |
| NP_849194.1 | BTB/POZ domain-containing adapter for CUL3-mediated  RhoA degradation protein 1 | | | |  |
| AGGAGR | 1 | 1.57 | 8 |  |  |
| GPDPALLEATGGAAGAGGAGR | 2 | 2.23 | 8 |  |  |
| NP_001073889.1 | SH3 and multiple ankyrin repeat domains protein 3 | | |  |  |
| AGGAGR | 1 | 1.57 | 8 |  |  |
| YPVARR | 1 | 1.80 | 8 |  |  |
| NP_919431.2 | PH domain leucine-rich repeat-containing protein phosphatase 1 | | | |  |
| AGGAGR | 1 | 1.57 | 8 |  |  |
| LFCNSSLR | 2 | 2.10 | 9 |  |  |
| ** cluster of 2 proteins |  |  |  |  |  |
| NP_002002.3 | fibroblast growth factor receptor 4 isoform 1 precursor | | |  |  |
| NP_998812.1 | fibroblast growth factor receptor 4 isoform 1 precursor | | |  |  |
| NP_075252.2 | fibroblast growth factor receptor 4 isoform 2 precursor | | |  |  |
| LAPAGR | 1 | 1.53 | 9 |  |  |
| ARRPPGPDLSPDGPR | 2 | 2.76 | 9 |  |  |
| NP_001032412.2 | peroxisomal proliferator-activated receptor A-interacting  complex 285 kDa protein isoform 1 | | | |  |
| LAPAGR | 1 | 1.53 | 9 |  |  |
| SSSEVLVLAETLDGVR | 2 | 2.80 | 9 |  |  |
| ** cluster of 41 proteins |  |  |  |  |  |
| NP_000714.3 | voltage-dependent L-type calcium channel subunit beta-1 isoform 1 | | | |  |
| FAVR | 1 | 1.58 | 7 |  |  |
| QDTFDADTPGSR | 2 | 2.02 | 7 |  |  |
| NP_031372.2 | opioid growth factor receptor |  |  |  |  |
| FAVR | 1 | 1.58 | 7 |  |  |
| EPGVNWHAKPLTLR | 2 | 2.34 | 6 |  |  |
| NP_001092304.1 | homeobox protein GBX-1 |  |  |  |  |
| FAVR | 1 | 1.58 | 7 |  |  |
| QVKIWFQNR | 2 | 2.01 | 8 |  |  |
| NP_009131.2 | A-kinase anchor protein 13 isoform 2 | |  |  |  |
| NP_006729.4 | A-kinase anchor protein 13 isoform 1 | |  |  |  |
| EALLAQR | 1 | 1.89 | 7 |  |  |
| FAVR | 1 | 1.58 | 7 |  |  |
| NP_115921.2 | fibronectin type III domain-containing protein 1 precursor | | | |  |
| FAVR | 1 | 1.58 | 7 |  |  |
| RAPR | 1 | 1.71 | 7 |  |  |
| NP_065815.1 | caskin-1 |  |  |  |  |
| FAVR | 1 | 1.58 | 7 |  |  |
| LLLDSGINAHVR | 2 | 2.74 | 6 |  |  |
| NP_055938.1 | FERM domain-containing protein 4B | |  |  |  |
| FAVR | 1 | 1.58 | 7 |  |  |
| ISVSR | 1 | 1.78 | 7 |  |  |
| NP_003226.4 | thyroglobulin precursor |  |  |  |  |
| FQAPEPLNWTGSWDASKPR | 2 | 2.46 | 7 |  |  |
| EPPAR | 1 | 1.56 | 7 |  |  |
| NP_079478.1 | EF-hand domain-containing protein D1 isoform 1 | | |  |  |
| APTASADAELSAQLSRR | 2 | 2.67 | 8 |  |  |
| EPPAR | 1 | 1.56 | 7 |  |  |
| NP_149132.2 | mitogen-activated protein kinase kinase kinase 9 | | |  |  |
| AFWIGDEVAVKAARHDPD EDISQTIENVR | 3 | 2.52 | 7 |  |  |
| EPPAR | 1 | 1.56 | 7 |  |  |
| NP_055601.2 | rho guanine nucleotide exchange factor 17 | |  |  |  |
| RAPR | 1 | 1.71 | 7 |  |  |
| EPPAR | 1 | 1.56 | 7 |  |  |
| NP_001094889.1 | zinc finger protein 541 |  |  |  |  |
| EASPGSTR | 2 | 2.59 | 8 |  |  |
| RAPR | 1 | 1.71 | 7 |  |  |
| NP_003465.3 | disintegrin and metalloproteinase domain-containing protein 12  isoform 1 preproprotein | | | | |
| RAPR | 1 | 1.71 | 7 |  |  |
| KTLIRLLFTNKKTTIEKLR | 2 | 2.22 | 8 |  |  |
| NP_061496.2 | voltage-dependent T-type calcium channel subunit alpha-1G isoform 1 | | | | |
| NP_001243263.1 | voltage-dependent T-type calcium channel subunit alpha-1G isoform 26 | | | | |
| NP_938190.1 | voltage-dependent T-type calcium channel subunit alpha-1G isoform 14 | | | | |
| NP_938192.1 | voltage-dependent T-type calcium channel subunit alpha-1G isoform 9 | | | | |
| NP_938193.1 | voltage-dependent T-type calcium channel subunit alpha-1G isoform 7 | | | | |
| NP_938194.1 | voltage-dependent T-type calcium channel subunit alpha-1G isoform 5 | | | | |
| NP_938196.1 | voltage-dependent T-type calcium channel subunit alpha-1G isoform 11 | | | | |
| NP_938197.1 | voltage-dependent T-type calcium channel subunit alpha-1G isoform 6 | | | | |
| NP_938198.1 | voltage-dependent T-type calcium channel subunit alpha-1G isoform 8 | | | | |
| NP_938199.1 | voltage-dependent T-type calcium channel subunit alpha-1G isoform 4 | | | | |
| NP_938200.1 | voltage-dependent T-type calcium channel subunit alpha-1G isoform 10 | | | | |
| NP_938201.1 | voltage-dependent T-type calcium channel subunit alpha-1G isoform 12 | | | | |
| NP_938202.1 | voltage-dependent T-type calcium channel subunit alpha-1G isoform 13 | | | | |
| NP_938406.1 | voltage-dependent T-type calcium channel subunit alpha-1G isoform 3 | | | | |
| NP_938191.2 | voltage-dependent T-type calcium channel subunit alpha-1G isoform 2 | | | | |
| NP_001243253.1 | voltage-dependent T-type calcium channel subunit alpha-1G isoform 16 | | | | |
| NP_001243254.1 | voltage-dependent T-type calcium channel subunit alpha-1G isoform 17 | | | | |
| NP_001243255.1 | voltage-dependent T-type calcium channel subunit alpha-1G isoform 18 | | | | |
| NP_001243256.1 | voltage-dependent T-type calcium channel subunit alpha-1G isoform 19 | | | | |
| NP_001243257.1 | voltage-dependent T-type calcium channel subunit alpha-1G isoform 20 | | | | |
| NP_001243258.1 | voltage-dependent T-type calcium channel subunit alpha-1G isoform 21 | | | | |
| NP_001243259.1 | voltage-dependent T-type calcium channel subunit alpha-1G isoform 22 | | | | |
| NP_001243260.1 | voltage-dependent T-type calcium channel subunit alpha-1G isoform 23 | | | | |
| NP_001243261.1 | voltage-dependent T-type calcium channel subunit alpha-1G isoform 24 | | | | |
| NP_001243262.1 | voltage-dependent T-type calcium channel subunit alpha-1G isoform 25 | | | | |
| NP_001243288.1 | voltage-dependent T-type calcium channel subunit alpha-1G isoform 27 | | | | |
| NP_001243289.1 | voltage-dependent T-type calcium channel subunit alpha-1G isoform 28 | | | | |
| NP_001243290.1 | voltage-dependent T-type calcium channel subunit alpha-1G isoform 29 | | | | |
| QAAIRTDSLDVQGLGSR | 2 | 2.62 | 8 |  |  |
| RAPR | 1 | 1.71 | 7 |  |  |
| NP_001810.2 | secretogranin-1 precursor |  |  |  |  |
| SLGEKR | 1 | 1.57 | 6 |  |  |
| RAPR | 1 | 1.71 | 7 |  |  |
| NP_002512.1 | natriuretic peptides B preproprotein | |  |  |  |
| EVATEGIR | 2 | 2.12 | 6 |  |  |
| RAPR | 1 | 1.71 | 7 |  |  |
| NP_001120730.1 | HHIP-like protein 1 isoform a precursor | |  |  |  |
| SRLGKPFLNISR | 2 | 2.18 | 8 |  |  |
| RAPR | 1 | 1.71 | 7 |  |  |
| NP_065168.2 | gap junction gamma-2 protein |  |  |  |  |
| FLTR | 1 | 1.54 | 6 |  |  |
| RAPR | 1 | 1.71 | 7 |  |  |
| NP_002195.1 | integrin alpha-3 isoform a precursor | |  |  |  |
| NP_005492.1 | integrin alpha-3 isoform b precursor | |  |  |  |
| LAGAPR | 1 | 1.54 | 8 |  |  |
| RAPR | 1 | 1.71 | 7 |  |  |
| NP_112571.2 | Fc receptor-like protein 5 isoform 1 precursor | | |  |  |
| NP_001182317.1 | Fc receptor-like protein 5 isoform 2 precursor | | |  |  |
| YLGKEILR | 2 | 2.38 | 6 |  |  |
| RAPR | 1 | 1.71 | 7 |  |  |
| NP_443171.2 | Fc receptor-like protein 3 precursor | |  |  |  |
| ENVPR | 1 | 1.66 | 8 |  |  |
| RAPR | 1 | 1.71 | 7 |  |  |
| NP_787098.3 | PKHD domain-containing transmembrane protein C17orf101 isoform 2 | | | |  |
| NP_078924.1 | PKHD domain-containing transmembrane protein C17orf101 isoform 1 | | | |  |
| FMFMEEGANKTVEPR | 2 | 2.38 | 6 |  |  |
| RAPR | 1 | 1.71 | 7 |  |  |
| NP_037505.1 | probable palmitoyltransferase ZDHHC8 isoform 2 | | |  |  |
| RAPR | 1 | 1.71 | 7 |  |  |
| SPGLAR | 1 | 1.53 | 6 |  |  |
| NP_940863.3 | LON peptidase N-terminal domain and RING finger protein 2 | | | |  |
| LGDALAR | 1 | 1.67 | 7 |  |  |
| RAPR | 1 | 1.71 | 7 |  |  |
| NP_954974.2 | protein FAM179A |  |  |  |  |
| RAPR | 1 | 1.71 | 7 |  |  |
| LSEGLAASSR | 1 | 1.78 | 6 |  |  |
| NP_001374.3 | diphthamide biosynthesis protein 1 | |  |  |  |
| QEAIATAR | 2 | 2.32 | 8 |  |  |
| RAPR | 1 | 1.71 | 7 |  |  |
| NP_872383.1 | outer dense fiber protein 3-like protein 2 | |  |  |  |
| SCTPAYSMQGRAKSR | 2 | 2.68 | 8 |  |  |
| RAPR | 1 | 1.71 | 7 |  |  |
| NP_038470.1 | stomatin-like protein 2 |  |  |  |  |
| ILEPGLNILIPVLDR | 2 | 4.16 | 7 |  |  |
| ATVLESEGTR | 2 | 2.82 | 7 |  |  |
| RAPR | 1 | 1.71 | 7 |  |  |
| NP_115599.2 | pecanex-like protein 3 |  |  |  |  |
| AEPPDPLPDKMRQSVR | 2 | 2.99 | 8 |  |  |
| RAPR | 1 | 1.71 | 7 |  |  |
| NP_055514.3 | zinc finger protein 646 |  |  |  |  |
| FLTR | 1 | 1.54 | 6 |  |  |
| HFPSLPELSRHR | 2 | 2.18 | 6 |  |  |
| NP_001184036.1 | schwannomin-interacting protein 1 isoform 2 | | |  |  |
| NP_001184037.1 | schwannomin-interacting protein 1 isoform 3 | | |  |  |
| NP_001184038.1 | schwannomin-interacting protein 1 isoform 4 | | |  |  |
| NP_001184042.1 | IQ motif containing J-schwannomin interacting protein 1 fusion protein isoform 1 | | | | |
| NP_055390.1 | schwannomin-interacting protein 1 isoform 1 | | |  |  |
| NP_001184043.1 | IQ motif containing J-schwannomin interacting protein 1 fusion protein isoform 2 | | | | |
| FLTR | 1 | 1.54 | 6 |  |  |
| HAESQQKHMAEKMPAK | 2 | 2.18 | 6 |  |  |
| NP_065175.4 | valine--tRNA ligase, mitochondrial isoform 2 precursor | | |  |  |
| NP_001161206.1 | valine--tRNA ligase, mitochondrial isoform 1 | | |  |  |
| FLTR | 1 | 1.54 | 6 |  |  |
| FHSVSTQSEPHGSPISR | 2 | 2.11 | 7 |  |  |
| NP_758952.4 | voltage-dependent calcium channel subunit alpha-2/delta-4 | | | |  |
| FLTR | 1 | 1.54 | 6 |  |  |
| FPLWYRQASEHPAGSFVFNLR | 3 | 2.88 | 6 |  |  |
| NP_065782.2 | aryl hydrocarbon receptor repressor isoform 1 | | |  |  |
| NP_001229341.1 | aryl hydrocarbon receptor repressor isoform 2 | | |  |  |
| FLTR | 1 | 1.54 | 6 |  |  |
| QPAAGAPSPGDSCPLAGSAVLEGR | 2 | 3.09 | 6 |  |  |
| NP_001180462.1 | serine/threonine-protein kinase Nek4 isoform 2 | | |  |  |
| NP_003148.2 | serine/threonine-protein kinase Nek4 isoform 1 | | |  |  |
| FLTR | 1 | 1.54 | 6 |  |  |
| TNIIKVGDLGIAR | 2 | 2.20 | 7 |  |  |
| NP_036385.3 | transketolase-like protein 1 isoform a | |  |  |  |
| NP_001139405.1 | transketolase-like protein 1 isoform b | |  |  |  |
| NP_001139406.1 | transketolase-like protein 1 isoform c | |  |  |  |
| VVVLDGDTR | 1 | 1.75 | 5 |  |  |
| FLTR | 1 | 1.54 | 6 |  |  |
| NP_055950.1 | nuclear pore complex protein Nup205 | |  |  |  |
| FLTR | 1 | 1.54 | 6 |  |  |
| SVSGFLHFDTATKVR | 2 | 2.13 | 7 |  |  |
| NP_872407.1 | PH domain-containing protein C10orf81 isoform 1 | | |  |  |
| NP_001180363.1 | PH domain-containing protein C10orf81 isoform 2 | | |  |  |
| NP_001180364.1 | PH domain-containing protein C10orf81 isoform 2 | | |  |  |
| FLTR | 1 | 1.54 | 6 |  |  |
| QDIKATQQNTEEELSLGNKR | 3 | 2.51 | 6 |  |  |
| NP_060526.2 | transmembrane protein 39B |  |  |  |  |
| FIGSIVKEASQR | 2 | 2.01 | 5 |  |  |
| FLTR | 1 | 1.54 | 6 |  |  |
| NP_001002837.1 | phosphatidylinositol 4,5-bisphosphate 5-phosphatase A | | |  |  |
| EALAPNSLSPSPQGHR | 2 | 2.23 | 5 |  |  |
| SPGLAR | 1 | 1.53 | 6 |  |  |
| NP_000896.1 | pro-neuropeptide Y preproprotein | |  |  |  |
| YYSALRHYINLITR | 2 | 2.22 | 9 |  |  |
| ENVPR | 1 | 1.66 | 8 |  |  |
| ** cluster of 48 proteins |  |  |  |  |  |
| NP_059509.1 | ubiquilin-3 |  |  |  |  |
| NP_001138818.1 | RNA-binding motif protein, X-linked-like-3 | |  |  |  |
| NP_057524.3 | inactive serine/threonine-protein kinase VRK3 isoform 1 | | |  |  |
| NP_001020949.1 | inactive serine/threonine-protein kinase VRK3 isoform 2 | | |  |  |
| GSGSR | 1 | 1.59 | 4 |  |  |
| EALP | 1 | 1.50 | 3 |  |  |
| FLVLPSLGR | 2 | 2.06 | 4 |  |  |
| NP_001104490.1 | transcription elongation factor SPT5 isoform a | | |  |  |
| NP_003160.2 | transcription elongation factor SPT5 isoform a | | |  |  |
| NP_001124296.1 | transcription elongation factor SPT5 isoform a | | |  |  |
| NP_001124297.1 | transcription elongation factor SPT5 isoform b | | |  |  |
| GSGSR | 1 | 1.59 | 4 |  |  |
| GGFGSPGGGSGGMSRGR | 2 | 2.45 | 3 |  |  |
| NP_003684.2 | scavenger receptor class F member 1 isoform 1 precursor | | |  |  |
| GSGSR | 1 | 1.59 | 4 |  |  |
| HFGSFQKGQAEAKVKR | 2 | 2.08 | 4 |  |  |
| NP_055246.2 | paladin |  |  |  |  |
| GSGSR | 1 | 1.59 | 4 |  |  |
| VESLELAIR | 1 | 1.99 | 5 |  |  |
| NP_055935.4 | Alstrom syndrome protein 1 |  |  |  |  |
| VRAHAWNMKFNLAHDC GYSISELNEDDR | 3 | 3.02 | 2 |  |  |
| EAGR | 1 | 1.53 | 2 |  |  |
| NP_001180278.1 | myocyte-specific enhancer factor 2C isoform 5 | | |  |  |
| NP_001180279.1 | myocyte-specific enhancer factor 2C isoform 1 | | |  |  |
| NP_002388.2 | myocyte-specific enhancer factor 2C isoform 1 | | |  |  |
| NEFHSPIGLTRPSPDER | 2 | 2.12 | 3 |  |  |
| EAGR | 1 | 1.53 | 2 |  |  |
| NP_003021.3 | short stature homeobox protein 2 isoform b | |  |  |  |
| NP_001157150.1 | short stature homeobox protein 2 isoform c | |  |  |  |
| NP_006875.2 | short stature homeobox protein 2 isoform a | |  |  |  |
| AAGGGGGGGGGGGGGGGG GGVGGGGAGGGAGGGR | 2 | 2.34 | 1 |  |  |
| EAGR | 1 | 1.53 | 2 |  |  |
| NP_001129495.1 | transcription factor NF-E2 45 kDa subunit isoform 2 | | |  |  |
| NP_001185863.1 | multidrug resistance-associated protein 7 isoform MRP7 | | |  |  |
| NP_258261.2 | multidrug resistance-associated protein 7 isoform MRP7A | | |  |  |
| NP_006154.1 | transcription factor NF-E2 45 kDa subunit isoform 1 | | |  |  |
| LALVR | 1 | 1.67 | 1 |  |  |
| EAGR | 1 | 1.53 | 2 |  |  |
| NP_055840.2 | myelin transcription factor 1-like protein | |  |  |  |
| EAGR | 1 | 1.53 | 2 |  |  |
| SKGVR | 1 | 1.50 | 1 |  |  |
| NP_004876.2 | neuropeptide FF receptor 2 isoform 1 | |  |  |  |
| QTAKSSWSR | 2 | 2.01 | 3 |  |  |
| EAGR | 1 | 1.53 | 2 |  |  |
| NP_004464.2 | forkhead box protein E1 |  |  |  |  |
| NP_065194.2 | gamma-tubulin complex component 6 | |  |  |  |
| NP_116166.9 | G-protein coupled receptor 124 precursor | |  |  |  |
| EAGR | 1 | 1.53 | 2 |  |  |
| GAGGR | 1 | 1.77 | 2 |  |  |
| NP_001035500.1 | uncharacterized protein C9orf131 isoform B | |  |  |  |
| NP_001035501.1 | uncharacterized protein C9orf131 isoform C | |  |  |  |
| NP_001035502.1 | uncharacterized protein C9orf131 isoform D | |  |  |  |
| NP_976044.2 | uncharacterized protein C9orf131 isoform A | |  |  |  |
| ESSLEDPSR | 2 | 2.11 | 3 |  |  |
| EAGR | 1 | 1.53 | 2 |  |  |
| RASDILTPR | 2 | 2.43 | 3 |  |  |
| NP_001077430.1 | WD repeat-containing protein 62 isoform 1 | |  |  |  |
| NP_775907.4 | WD repeat-containing protein 62 isoform 2 | |  |  |  |
| NP_001011719.1 | arylsulfatase H |  |  |  |  |
| NP_004415.2 | transcription factor E4F1 |  |  |  |  |
| NP_055831.1 | microtubule-associated serine/threonine-protein kinase 3 | | | |  |
| NQIQQVFVER | 2 | 2.49 | 2 |  |  |
| EALP | 1 | 1.50 | 3 |  |  |
| EAGR | 1 | 1.53 | 2 |  |  |
| ASSSGGSGGGSGGR | 2 | 2.44 | 2 |  |  |
| NP_001020331.1 | CD97 antigen isoform 3 preproprotein | |  |  |  |
| NP_001775.2 | CD97 antigen isoform 2 preproprotein | |  |  |  |
| NP_510966.1 | CD97 antigen isoform 1 preproprotein | |  |  |  |
| EAGR | 1 | 1.53 | 2 |  |  |
| QVGLR | 1 | 1.73 | 2 |  |  |
| NP_510880.2 | unconventional myosin-XVIIIa isoform a | |  |  |  |
| NP_976063.1 | unconventional myosin-XVIIIa isoform b | |  |  |  |
| SRAPELVTKKFPVDLR | 2 | 2.21 | 3 |  |  |
| EAGR | 1 | 1.53 | 2 |  |  |
| NP_060899.2 | transcription factor TFIIIB component B'' homolog | | |  |  |
| EAGR | 1 | 1.53 | 2 |  |  |
| SSTEKTGGDNDVEESSR | 2 | 2.59 | 3 |  |  |
| NP_004517.2 | DNA replication licensing factor MCM2 | |  |  |  |
| DYVIEDDVNMAIR | 2 | 2.45 | 3 |  |  |
| EAGR | 1 | 1.53 | 2 |  |  |
| NP_060830.2 | gamma-taxilin isoform 1 |  |  |  |  |
| NP_001162154.1 | gamma-taxilin isoform 2 |  |  |  |  |
| VEEAARGR | 2 | 2.22 | 1 |  |  |
| EAGR | 1 | 1.53 | 2 |  |  |
| NP_001009999.1 | lysine-specific histone demethylase 1A isoform a | | |  |  |
| NP_055828.2 | lysine-specific histone demethylase 1A isoform b | | |  |  |
| EAGRIADQFLGAMYTLPR | 2 | 2.24 | 2 |  |  |
| EAGR | 1 | 1.53 | 2 |  |  |
| NP_115901.2 | PDZ domain-containing protein 4 | |  |  |  |
| NPKTGLTLERVGPESSPYLSR | 2 | 2.23 | 2 |  |  |
| EAGR | 1 | 1.53 | 2 |  |  |
| NP_055715.3 | cordon-bleu protein-like 1 |  |  |  |  |
| QSLLTAIR | 2 | 2.38 | 2 |  |  |
| EAGR | 1 | 1.53 | 2 |  |  |
| NP_570629.2 | dipeptidyl aminopeptidase-like protein 6 isoform 1 | | |  |  |
| ERGGGGGGAGGRPR | 2 | 2.20 | 2 |  |  |
| GAGGR | 1 | 1.77 | 2 |  |  |
| NP_004332.2 | CAD protein |  |  |  |  |
| EALP | 1 | 1.50 | 3 |  |  |
| GAGGR | 1 | 1.77 | 2 |  |  |
| NP_065905.2 | lisH domain and HEAT repeat-containing protein KIAA1468 | | | |  |
| LLVAESCGALAPYLPKEIR | 2 | 2.15 | 2 |  |  |
| GAGGR | 1 | 1.77 | 2 |  |  |
| NP_689848.1 | oncoprotein-induced transcript 3 protein precursor | | |  |  |
| NSPLEIMSR | 1 | 2.03 | 4 |  |  |
| EALP | 1 | 1.50 | 3 |  |  |
| NP_001008781.2 | protocadherin Fat 3 precursor |  |  |  |  |
| FEIDKASGAIR | 2 | 2.08 | 4 |  |  |
| EALP | 1 | 1.50 | 3 |  |  |
| NP_001159530.1 | relaxin receptor 2 isoform 2 |  |  |  |  |
| NP_570718.1 | relaxin receptor 2 isoform 1 |  |  |  |  |
| EALP | 1 | 1.50 | 3 |  |  |
| EVAVANR | 1 | 1.71 | 2 |  |  |
| NP_005754.2 | alpha-aminoadipic semialdehyde synthase, mitochondrial | | | |  |
| EALP | 1 | 1.50 | 3 |  |  |
| NSSQAVQAVR | 2 | 2.10 | 2 |  |  |
| NP_055927.2 | microtubule-associated serine/threonine-protein kinase 2 | | | |  |
| NP_004827.4 | eukaryotic translation initiation factor 2-alpha kinase 3 precursor | | | |  |
| EALP | 1 | 1.50 | 3 |  |  |
| METR | 1 | 1.63 | 2 |  |  |
| NP_054789.2 | uncharacterized protein C6orf15 precursor | |  |  |  |
| EALP | 1 | 1.50 | 3 |  |  |
| YPGGSWGNINR | 1 | 1.78 | 2 |  |  |
| NP_065811.1 | E3 ubiquitin-protein ligase HECW2 | |  |  |  |
| SLPSVRQDVSR | 2 | 2.56 | 2 |  |  |
| EALP | 1 | 1.50 | 3 |  |  |
| NP_783297.2 | tight junction protein ZO-1 isoform b | |  |  |  |
| NP_003248.3 | tight junction protein ZO-1 isoform a | |  |  |  |
| GFGIAISGGR | 2 | 2.15 | 2 |  |  |
| EALP | 1 | 1.50 | 3 |  |  |
| NP_061939.3 | MAGE-like protein 2 |  |  |  |  |
| QAPQAR | 1 | 1.78 | 3 |  |  |
| EALP | 1 | 1.50 | 3 |  |  |
| NP_113678.2 | lon protease homolog 2, peroxisomal | |  |  |  |
| EALP | 1 | 1.50 | 3 |  |  |
| DIITR | 1 | 1.84 | 4 |  |  |
| NP_065928.2 | dynein heavy chain 2, axonemal |  |  |  |  |
| EALP | 1 | 1.50 | 3 |  |  |
| LYQLMSEPQFSR | 2 | 2.06 | 3 |  |  |
| NP_071765.2 | roundabout homolog 3 precursor | |  |  |  |
| FSVSPRGQLNITAVQR | 2 | 2.52 | 4 |  |  |
| EALP | 1 | 1.50 | 3 |  |  |
| NP_055820.2 | protein KIAA0284 isoform 2 |  |  |  |  |
| EALP | 1 | 1.50 | 3 |  |  |
| KPTMAEARAVSR | 2 | 2.22 | 3 |  |  |
| NP_009135.4 | probable ATP-dependent RNA helicase DDX20 | | |  |  |
| EALP | 1 | 1.50 | 3 |  |  |
| NKVIEQRVPVLASSSQSGDSES DSDSYSSR | 3 | 2.74 | 2 |  |  |
| NP_996816.2 | usherin isoform B |  |  |  |  |
| EALP | 1 | 1.50 | 3 |  |  |
| TGPAPPEGLR | 2 | 2.48 | 4 |  |  |
| NGSILCDQIGGQCNCKRHVSGR | 3 | 2.93 | 3 |  |  |
| NP_078800.3 | probable cation-transporting ATPase 13A3 | |  |  |  |
| EALP | 1 | 1.50 | 3 |  |  |
| FPFSSALQR | 1 | 1.52 | 2 |  |  |
| NP_005910.1 | myocyte enhancer factor 2B isoform b | |  |  |  |
| EALP | 1 | 1.50 | 3 |  |  |
| SPGGTSPERSPGTAR | 2 | 2.37 | 2 |  |  |
| NP_115791.3 | caspase recruitment domain-containing protein 11 | | |  |  |
| EALP | 1 | 1.50 | 3 |  |  |
| EGHQLLLLEGCIRGER | 2 | 2.29 | 3 |  |  |
| ERDQAFHSR | 2 | 2.18 | 2 |  |  |
| NP_001034679.2 | probable ubiquitin carboxyl-terminal hydrolase FAF-X isoform 3 | | | |  |
| NP_001034680.2 | probable ubiquitin carboxyl-terminal hydrolase FAF-X isoform 4 | | | |  |
| NGGDGERNR | 2 | 2.05 | 3 |  |  |
| METR | 1 | 1.63 | 2 |  |  |
| NP_061322.2 | matrin-3 isoform a |  |  |  |  |
| NP_954659.1 | matrin-3 isoform a |  |  |  |  |
| NP_001181883.1 | matrin-3 isoform a |  |  |  |  |
| NP_001181884.1 | matrin-3 isoform a |  |  |  |  |
| GPSLNPVLDYDHGSR | 2 | 2.11 | 2 |  |  |
| TEEGPTLSYGR | 2 | 3.57 | 3 |  |  |
| METR | 1 | 1.63 | 2 |  |  |
| GPGPLQER | 2 | 2.33 | 3 |  |  |
| NP_004645.2 | probable ubiquitin carboxyl-terminal hydrolase FAF-Y | | |  |  |
| FWFTHNVLFNVSNR | 2 | 2.38 | 3 |  |  |
| METR | 1 | 1.59 | 3 |  |  |
| NP_065972.3 | ninein isoform 2 |  |  |  |  |
| NP_891989.2 | ninein isoform 1 |  |  |  |  |
| NP_891991.1 | ninein isoform 5 |  |  |  |  |
| METR | 1 | 1.63 | 2 |  |  |
| SEEYEAEGQLR | 2 | 2.58 | 3 |  |  |
| NP_001013694.4 | uncharacterized protein C17orf97 | |  |  |  |
| METR | 1 | 1.63 | 2 |  |  |
| EGGAAGPRGSR | 2 | 2.45 | 3 |  |  |
| NP_056016.1 | ribosome biogenesis protein BOP1 | |  |  |  |
| MAGSRGAGR | 2 | 2.60 | 2 |  |  |
| QVGLR | 1 | 1.73 | 2 |  |  |
| ** cluster of 4 proteins |  |  |  |  |  |
| NP_570925.2 | receptor-type tyrosine-protein phosphatase S isoform 4 precursor | | | |  |
| NP_002841.3 | receptor-type tyrosine-protein phosphatase S isoform 1 precursor | | | |  |
| NP_570923.2 | receptor-type tyrosine-protein phosphatase S isoform 3 precursor | | | |  |
| NP_570924.2 | receptor-type tyrosine-protein phosphatase S isoform 2 precursor | | | |  |
| ASAPR | 1 | 1.67 | 10 |  |  |
| TGEQAPASAPR | 2 | 2.11 | 11 |  |  |
| NP_001098673.1 | RUN and SH3 domain-containing protein 1 isoform a | | |  |  |
| NP_001098674.1 | RUN and SH3 domain-containing protein 1 isoform b | | |  |  |
| NP_001098675.1 | RUN and SH3 domain-containing protein 1 isoform c | | |  |  |
| NP_055143.2 | RUN and SH3 domain-containing protein 1 isoform d | | |  |  |
| ASAPR | 1 | 1.67 | 10 |  |  |
| FPLSR | 1 | 1.76 | 10 |  |  |
| NP_056224.3 | E1A-binding protein p400 |  |  |  |  |
| VELEEKR | 2 | 2.23 | 10 |  |  |
| ASAPR | 1 | 1.67 | 10 |  |  |
| NP_079441.1 | RNA polymerase II elongation factor ELL3 | |  |  |  |
| VELEEKR | 2 | 2.23 | 10 |  |  |
| EPVQALPSSASR | 2 | 2.46 | 11 |  |  |
| ** cluster of 2 proteins |  |  |  |  |  |
| NP_002267.2 | keratin, type I cytoskeletal 19 |  |  |  |  |
| NP_000215.1 | keratin, type I cytoskeletal 18 |  |  |  |  |
| NP_954657.1 | keratin, type I cytoskeletal 18 |  |  |  |  |
| LAADDFR | 2 | 2.82 | 12 |  |  |
| IVLQIDNAR | 2 | 2.65 | 11 |  |  |
| NP_000412.3 | keratin, type I cytoskeletal 10 |  |  |  |  |
| SQYEQLAEQNR | 2 | 4.81 | 11 |  |  |
| SLLEGEGSSGGGGR | 2 | 4.71 | 11 |  |  |
| LAADDFR | 2 | 2.82 | 12 |  |  |
| LENEIQTYR | 2 | 4.05 | 11 |  |  |
| ** cluster of 2 proteins |  |  |  |  |  |
| NP_689476.2 | tropomyosin alpha-3 chain isoform 1 | |  |  |  |
| NP_001036816.1 | tropomyosin alpha-3 chain isoform 4 | |  |  |  |
| NP_001036817.1 | tropomyosin alpha-3 chain isoform 3 | |  |  |  |
| NP_001036818.1 | tropomyosin alpha-3 chain isoform 5 | |  |  |  |
| NP_003281.1 | tropomyosin alpha-4 chain isoform 2 | |  |  |  |
| NP_705935.1 | tropomyosin alpha-3 chain isoform 2 | |  |  |  |
| NP_001138632.1 | tropomyosin alpha-4 chain isoform 1 | |  |  |  |
| NP_003280.2 | tropomyosin beta chain isoform 1 | |  |  |  |
| NP_998839.1 | tropomyosin beta chain isoform 2 | |  |  |  |
| NP_001018004.1 | tropomyosin alpha-1 chain isoform 3 | |  |  |  |
| NP_001018005.1 | tropomyosin alpha-1 chain isoform 1 | |  |  |  |
| NP_001018007.1 | tropomyosin alpha-1 chain isoform 2 | |  |  |  |
| NP_001018008.1 | tropomyosin alpha-1 chain isoform 6 | |  |  |  |
| EQAEAEVASLNR | 2 | 4.06 | 9 |  |  |
| KYEEVAR | 2 | 2.93 | 9 |  |  |
| RIQLVEEELDR | 2 | 3.56 | 9 |  |  |
| IQLVEEELDR | 2 | 3.90 | 9 |  |  |
| AEFAER | 1 | 1.90 | 9 |  |  |
| KLVIIEGDLER | 2 | 2.95 | 9 |  |  |
| NP_000357.3 | tropomyosin alpha-1 chain isoform 5 | |  |  |  |
| NP_001018006.1 | tropomyosin alpha-1 chain isoform 4 | |  |  |  |
| NP_001018020.1 | tropomyosin alpha-1 chain isoform 7 | |  |  |  |
| AELSEGQVR | 2 | 3.15 | 9 |  |  |
| KYEEVAR | 2 | 2.93 | 9 |  |  |
| RIQLVEEELDR | 2 | 3.56 | 9 |  |  |
| IQLVEEELDR | 2 | 3.90 | 9 |  |  |
| AEFAER | 1 | 1.90 | 9 |  |  |
| ** cluster of 3 proteins |  |  |  |  |  |
| NP_006253.2 | peripherin |  |  |  |  |
| KLLEGEESR | 2 | 3.55 | 7 |  |  |
| LLEGEESR | 2 | 3.14 | 7 |  |  |
| FLEQQNAALRGELSQAR | 2 | 3.94 | 6 |  |  |
| NP_005547.3 | keratin, type II cytoskeletal 7 |  |  |  |  |
| AKQEELEAALQR | 2 | 3.70 | 6 |  |  |
| GQLEALQVDGGR | 2 | 4.77 | 6 |  |  |
| KLLEGEESR | 2 | 3.55 | 7 |  |  |
| SAYGGPVGAGIR | 2 | 4.12 | 6 |  |  |
| LPDIFEAQIAGLR | 2 | 3.89 | 7 |  |  |
| AKLEAAIAEAEER | 2 | 3.86 | 6 |  |  |
| LDADPSLQR | 2 | 3.09 | 6 |  |  |
| LLEGEESR | 2 | 3.14 | 7 |  |  |
| EVTINQSLLAPLR | 2 | 4.55 | 6 |  |  |
| NP_001918.3 | desmin |  |  |  |  |
| KLLEGEESR | 2 | 3.55 | 7 |  |  |
| TSGGAGGLGSLR | 2 | 2.69 | 6 |  |  |
| LLEGEESR | 2 | 3.14 | 7 |  |  |
| ** cluster of 3 proteins |  |  |  |  |  |
| NP_694881.1 | heat shock cognate 71 kDa protein isoform 2 | |  |  |  |
| NP_005336.3 | heat shock 70 kDa protein 1A/1B |  |  |  |  |
| NP_005337.2 | heat shock 70 kDa protein 1A/1B |  |  |  |  |
| NP_006588.1 | heat shock cognate 71 kDa protein isoform 1 | |  |  |  |
| TTPSYVAFTDTER | 2 | 4.34 | 10 |  |  |
| VEIIANDQGNR | 2 | 4.18 | 10 |  |  |
| IINEPTAAAIAYGLDR | 2 | 4.87 | 10 |  |  |
| NP_002146.2 | heat shock 70 kDa protein 6 |  |  |  |  |
| TTPSYVAFTDTER | 2 | 4.34 | 10 |  |  |
| IINEPTAAAIAYGLDR | 2 | 4.87 | 10 |  |  |
| NP_005518.3 | heat shock 70 kDa protein 1-like |  |  |  |  |
| NP_068814.2 | heat shock-related 70 kDa protein 2 | |  |  |  |
| GTLEPVEKALR | 2 | 2.33 | 10 |  |  |
| TTPSYVAFTDTER | 2 | 4.34 | 10 |  |  |
| VEIIANDQGNR | 2 | 4.18 | 10 |  |  |
| ** cluster of 4 proteins |  |  |  |  |  |
| NP_001398.2 | cadherin EGF LAG seven-pass G-type receptor 3 precursor | | |  |  |
| GLGGR | 1 | 1.60 | 9 |  |  |
| TQDQDSQR | 2 | 2.02 | 10 |  |  |
| NP_001123616.1 | latent-transforming growth factor beta-binding protein 3 isoform 1 precursor | | | | |
| NP_066548.2 | latent-transforming growth factor beta-binding protein 3 isoform 2 precursor | | | | |
| MNGGQCSSR | 2 | 2.07 | 10 |  |  |
| GLGGR | 1 | 1.60 | 9 |  |  |
| NP_597707.1 | ankyrin repeat and SAM domain-containing protein 3 isoform 1 | | | |  |
| GLGGR | 1 | 1.60 | 9 |  |  |
| FLLDSGANANVR | 2 | 2.03 | 8 |  |  |
| NP_002365.3 | microtubule-associated protein 2 isoform 1 | |  |  |  |
| GLGGR | 1 | 1.60 | 9 |  |  |
| GRISTPER | 2 | 2.55 | 9 |  |  |
| ** cluster of 70 proteins |  |  |  |  |  |
| NP_001026884.3 | inverted formin-2 isoform 2 |  |  |  |  |
| NP_071934.3 | inverted formin-2 isoform 1 |  |  |  |  |
| QGLL | 1 | 1.61 | 11 |  |  |
| EGGPR | 1 | 1.53 | 11 |  |  |
| NP_001120936.1 | zinc finger protein 469 |  |  |  |  |
| VTCPSTGLGLGR | 2 | 2.51 | 10 |  |  |
| EGGPR | 1 | 1.53 | 11 |  |  |
| NP_112557.2 | cyclin-dependent kinase 13 isoform 2 | |  |  |  |
| NP_003709.3 | cyclin-dependent kinase 13 isoform 1 | |  |  |  |
| QGLL | 1 | 1.61 | 11 |  |  |
| SPAGGG | 1 | 1.50 | 10 |  |  |
| NP_055538.2 | syntaphilin |  |  |  |  |
| GGSPAR | 1 | 1.51 | 9 |  |  |
| SPAGGG | 1 | 1.59 | 9 |  |  |
| NP_061978.6 | tenascin-X isoform 1 precursor |  |  |  |  |
| AGPFSSR | 1 | 1.64 | 9 |  |  |
| SPAGGG | 1 | 1.59 | 9 |  |  |
| NP_006827.1 | translational activator GCN1 |  |  |  |  |
| MKIDPEAFITR | 2 | 2.36 | 12 |  |  |
| DPGVR | 1 | 1.75 | 12 |  |  |
| NP_149014.3 | rho GTPase-activating protein SYDE1 | |  |  |  |
| QGLL | 1 | 1.61 | 11 |  |  |
| DPGVR | 1 | 1.75 | 12 |  |  |
| NP_000085.1 | collagen alpha-1(VII) chain precursor | |  |  |  |
| GEPGSVPNVDR | 1 | 1.68 | 12 |  |  |
| DPGVR | 1 | 1.75 | 12 |  |  |
| NP_542417.2 | protein PRRC2A |  |  |  |  |
| NP_004629.3 | protein PRRC2A |  |  |  |  |
| GVPSR | 1 | 1.50 | 10 |  |  |
| QGSVTAPGGHPR | 2 | 2.13 | 11 |  |  |
| VNSGLSSDPHFEEPGPMVR | 2 | 2.52 | 10 |  |  |
| NP_443128.2 | CUB and sushi domain-containing protein 2 | |  |  |  |
| GVPSR | 1 | 1.50 | 10 |  |  |
| VGSPR | 1 | 1.70 | 9 |  |  |
| NP_056982.1 | zinc finger and BTB domain-containing protein 7A | | |  |  |
| NP_001073988.2 | BAH and coiled-coil domain-containing protein 1 | | |  |  |
| QGLL | 1 | 1.61 | 11 |  |  |
| GVPSR | 1 | 1.50 | 10 |  |  |
| NP_612401.2 | SH3KBP1-binding protein 1 |  |  |  |  |
| MAAAATAAEGVPSR | 2 | 2.09 | 11 |  |  |
| GVPSR | 1 | 1.50 | 10 |  |  |
| NP_073603.2 | ceramide kinase |  |  |  |  |
| VGSPR | 1 | 1.70 | 9 |  |  |
| LFARGIEENPKPDSHS | 2 | 3.22 | 10 |  |  |
| NP_060023.1 | DNA helicase INO80 |  |  |  |  |
| VLLTR | 1 | 1.58 | 10 |  |  |
| QGLL | 1 | 1.61 | 11 |  |  |
| NP_919269.2 | xin actin-binding repeat-containing protein 1 isoform 1 | | |  |  |
| NP_001185550.1 | xin actin-binding repeat-containing protein 1 isoform 2 | | |  |  |
| QGLL | 1 | 1.61 | 11 |  |  |
| EPAASGDVQGTR | 2 | 2.34 | 12 |  |  |
| NP_001242983.1 | uncharacterized protein C9orf72 isoform a | |  |  |  |
| NP_060795.1 | uncharacterized protein C9orf72 isoform a | |  |  |  |
| ILGPR | 1 | 1.84 | 10 |  |  |
| QGLL | 1 | 1.61 | 11 |  |  |
| NP_001171134.1 | telomere-associated protein RIF1 isoform 2 | |  |  |  |
| NP_001171135.1 | telomere-associated protein RIF1 isoform 2 | |  |  |  |
| NP_001171136.1 | telomere-associated protein RIF1 isoform 2 | |  |  |  |
| NP_060621.3 | telomere-associated protein RIF1 isoform 1 | |  |  |  |
| KKALISSR | 1 | 1.88 | 10 |  |  |
| QGLL | 1 | 1.61 | 11 |  |  |
| NP_003768.2 | dynein heavy chain 11, axonemal | |  |  |  |
| QGLL | 1 | 1.61 | 11 |  |  |
| VWDAYTGLEGTVKDMTASLR | 3 | 2.55 | 11 |  |  |
| NP_001229601.1 | tetratricopeptide repeat protein 34 | |  |  |  |
| QGLL | 1 | 1.61 | 11 |  |  |
| LRATCLAELQEFGR | 2 | 2.30 | 10 |  |  |
| NP_079406.3 | putative hexokinase HKDC1 |  |  |  |  |
| QGLL | 1 | 1.61 | 11 |  |  |
| FLYHMR | 1 | 1.74 | 10 |  |  |
| NP_055542.1 | histone-lysine N-methyltransferase MLL4 | |  |  |  |
| GGQSSRGGR | 2 | 2.02 | 12 |  |  |
| QGLL | 1 | 1.61 | 11 |  |  |
| NP_542937.2 | dachshund homolog 1 isoform a |  |  |  |  |
| NP_542938.2 | dachshund homolog 1 isoform b |  |  |  |  |
| NP_004383.3 | dachshund homolog 1 isoform c |  |  |  |  |
| QGLL | 1 | 1.61 | 11 |  |  |
| VLNDSLTPEIEADRSGGR | 2 | 2.35 | 12 |  |  |
| NP_219499.1 | cortactin-binding protein 2 |  |  |  |  |
| QGLL | 1 | 1.61 | 11 |  |  |
| NTVTQALSR | 1 | 1.91 | 10 |  |  |
| NP_079504.2 | TRAF3-interacting JNK-activating modulator | |  |  |  |
| QGLL | 1 | 1.61 | 11 |  |  |
| DLQMNQALR | 2 | 2.84 | 10 |  |  |
| NP_001002812.1 | myomegalin isoform 2 |  |  |  |  |
| NP_055459.4 | myomegalin isoform 1 |  |  |  |  |
| NP_001185763.2 | myomegalin isoform 9 |  |  |  |  |
| NP_001185761.1 | myomegalin isoform 8 |  |  |  |  |
| LVEQLSR | 1 | 1.95 | 10 |  |  |
| QGLL | 1 | 1.61 | 11 |  |  |
| NP_055816.2 | msx2-interacting protein |  |  |  |  |
| QGLL | 1 | 1.61 | 11 |  |  |
| VVLTR | 1 | 1.58 | 12 |  |  |
| NP_690595.1 | RNA-binding protein 10 isoform 2 | |  |  |  |
| NP_001191395.1 | RNA-binding protein 10 isoform 3 | |  |  |  |
| NP_001191396.1 | RNA-binding protein 10 isoform 4 | |  |  |  |
| NP_001191397.1 | RNA-binding protein 10 isoform 5 | |  |  |  |
| NP_005667.2 | RNA-binding protein 10 isoform 1 | |  |  |  |
| QGLL | 1 | 1.61 | 11 |  |  |
| SQDDGGENRSR | 2 | 2.30 | 10 |  |  |
| NP_001409.3 | eukaryotic translation initiation factor 4 gamma 2 isoform 1 | | | |  |
| NP_001166176.1 | eukaryotic translation initiation factor 4 gamma 2 isoform 1 | | | |  |
| QGLL | 1 | 1.61 | 11 |  |  |
| FSPTMGRHR | 1 | 1.81 | 10 |  |  |
| NP_056193.2 | vacuolar protein sorting-associated protein 13D isoform 1 | | | |  |
| NP_060626.2 | vacuolar protein sorting-associated protein 13D isoform 2 | | | |  |
| QGLL | 1 | 1.61 | 11 |  |  |
| LNFLQRVR | 2 | 2.31 | 12 |  |  |
| NP_055864.2 | trafficking kinesin-binding protein 2 | |  |  |  |
| LSIGESITNRR | 2 | 2.12 | 11 |  |  |
| QGLL | 1 | 1.61 | 11 |  |  |
| NP_055335.2 | myb-binding protein 1A isoform 2 | |  |  |  |
| NP_001099008.1 | myb-binding protein 1A isoform 1 | |  |  |  |
| QGLL | 1 | 1.61 | 11 |  |  |
| NSPLTVPMFLSLFSR | 2 | 2.65 | 10 |  |  |
| NP_689811.2 | probable E3 ubiquitin-protein ligase MARCH10 | | |  |  |
| NP_001094345.1 | probable E3 ubiquitin-protein ligase MARCH10 | | |  |  |
| QGLL | 1 | 1.61 | 11 |  |  |
| FAELMRLNHNQVER | 2 | 2.35 | 11 |  |  |
| NP_060595.3 | rho guanine nucleotide exchange factor 10-like protein isoform 1 | | | |  |
| QGLL | 1 | 1.61 | 11 |  |  |
| DILALRVGGR | 2 | 2.13 | 11 |  |  |
| NP_203754.2 | 182 kDa tankyrase-1-binding protein | |  |  |  |
| NLEVSSCVGSGGSSEARESA VGQMGWSGGLSLR | 3 | 2.50 | 12 |  |  |
| QGLL | 1 | 1.61 | 11 |  |  |
| NP_001171146.1 | patatin-like phospholipase domain-containing protein 5 isoform 2 | | | |  |
| NP_620169.1 | patatin-like phospholipase domain-containing protein 5 isoform 1 | | | |  |
| QGYLDALR | 1 | 1.68 | 12 |  |  |
| QGLL | 1 | 1.61 | 11 |  |  |
| NP_075573.2 | calpain-10 isoform c |  |  |  |  |
| NP_075571.1 | calpain-10 isoform a |  |  |  |  |
| QGLL | 1 | 1.61 | 11 |  |  |
| RAGAR | 1 | 1.50 | 11 |  |  |
| NP_001231903.1 | serine/threonine-protein phosphatase PP1-gamma catalytic subunit isoform 2 | | | | |
| NP_002701.1 | serine/threonine-protein phosphatase PP1-gamma catalytic subunit isoform 1 | | | | |
| MADLDKLNIDSIIQR | 2 | 2.62 | 12 |  |  |
| QGLL | 1 | 1.61 | 11 |  |  |
| NP_699207.1 | coiled-coil domain-containing protein 96 | |  |  |  |
| DILTKTKQAR | 2 | 2.15 | 10 |  |  |
| QGLL | 1 | 1.61 | 11 |  |  |
| NP_055564.3 | uncharacterized protein KIAA0586 isoform 5 | |  |  |  |
| FNSPSPKSRPQRPKVIER | 2 | 2.07 | 12 |  |  |
| QGLL | 1 | 1.61 | 11 |  |  |
| NP_056236.2 | transmembrane protein 186 |  |  |  |  |
| TFGFLSR | 1 | 2.11 | 12 |  |  |
| QGLL | 1 | 1.61 | 11 |  |  |
| NP_001401.2 | multiple epidermal growth factor-like domains protein 8 precursor | | | |  |
| GAMYLLGGLTAGGVTR | 1 | 2.01 | 10 |  |  |
| QGLL | 1 | 1.61 | 11 |  |  |
| NP_005426.2 | rho guanine nucleotide exchange factor 5 | |  |  |  |
| NP_005551.3 | laminin subunit alpha-5 precursor | |  |  |  |
| QGLL | 1 | 1.61 | 11 |  |  |
| EAPAR | 1 | 1.66 | 11 |  |  |
| NP_001108225.1 | endoglin isoform 1 precursor |  |  |  |  |
| NP_000109.1 | endoglin isoform 2 precursor |  |  |  |  |
| QGLL | 1 | 1.61 | 11 |  |  |
| VLPGHSAGPR | 1 | 1.78 | 11 |  |  |
| NP_612361.1 | atherin |  |  |  |  |
| TDVLTGLSIR | 2 | 2.38 | 11 |  |  |
| QGLL | 1 | 1.61 | 11 |  |  |
| NP_079136.2 | N-lysine methyltransferase SETD6 isoform b | |  |  |  |
| NP_001153777.1 | N-lysine methyltransferase SETD6 isoform a | |  |  |  |
| GGLLER | 1 | 1.77 | 12 |  |  |
| QGLL | 1 | 1.61 | 11 |  |  |
| NP_689726.3 | meiosis inhibitor protein 1 |  |  |  |  |
| QGLL | 1 | 1.61 | 11 |  |  |
| DSEKAILQR | 1 | 1.59 | 11 |  |  |
| NP_000435.3 | phosphate-regulating neutral endopeptidase | | |  |  |
| QGLL | 1 | 1.61 | 11 |  |  |
| LEIKIAEIMIPHENR | 2 | 2.31 | 12 |  |  |
| NP_001073996.1 | unconventional myosin-VIIb |  |  |  |  |
| RAVVVIQAHAR | 1 | 2.11 | 10 |  |  |
| QGLL | 1 | 1.61 | 11 |  |  |
| NP_056327.4 | dynein heavy chain 1, axonemal |  |  |  |  |
| QGLL | 1 | 1.61 | 11 |  |  |
| KGVFGPPLGR | 2 | 2.32 | 12 |  |  |
| NP_940857.2 | SCO-spondin precursor |  |  |  |  |
| QGLL | 1 | 1.61 | 11 |  |  |
| EAGCPAGR | 2 | 2.38 | 11 |  |  |
| GLWNCTAR | 2 | 2.08 | 11 |  |  |
| NP_055901.2 | dendrin |  |  |  |  |
| QGLL | 1 | 1.61 | 11 |  |  |
| VAQLAGLPAPLRPER | 2 | 2.73 | 11 |  |  |
| NP_689914.2 | ATP-binding cassette sub-family A member 13 | | |  |  |
| LVGAISR | 1 | 1.69 | 12 |  |  |
| QGLL | 1 | 1.61 | 11 |  |  |
| NP_065867.2 | kinesin-like protein KIF17 isoform a | |  |  |  |
| NP_001116291.1 | kinesin-like protein KIF17 isoform b | |  |  |  |
| EEYEERLAR | 1 | 1.97 | 10 |  |  |
| QGLL | 1 | 1.61 | 11 |  |  |
| NP_001186978.2 | tetratricopeptide repeat protein 40 | |  |  |  |
| QGLL | 1 | 1.61 | 11 |  |  |
| TLAHGALAQLGSLQPLSVGCVEIR | 3 | 2.70 | 11 |  |  |
| NP_000102.1 | chloride anion exchanger |  |  |  |  |
| QGLL | 1 | 1.61 | 11 |  |  |
| KLIDAVGFSPLR | 2 | 2.36 | 10 |  |  |
| NP_057195.2 | telomere length regulation protein TEL2 homolog | | |  |  |
| QGPAGSPSR | 2 | 2.47 | 12 |  |  |
| QGLL | 1 | 1.61 | 11 |  |  |
| NP_057726.3 | spectrin beta chain, brain 4 |  |  |  |  |
| QLLAAFTIFR | 2 | 2.78 | 10 |  |  |
| QGLL | 1 | 1.61 | 11 |  |  |
| NP_001182457.1 | uncharacterized protein LOC100507050 precursor | | |  |  |
| YEQDADPR | 2 | 2.17 | 11 |  |  |
| QGLL | 1 | 1.61 | 11 |  |  |
| NP_113584.3 | E3 ubiquitin-protein ligase HUWE1 | |  |  |  |
| QGLL | 1 | 1.61 | 11 |  |  |
| LQAVQAR | 1 | 1.52 | 11 |  |  |
| NP_055318.2 | small subunit processome component 20 homolog | | |  |  |
| QGLL | 1 | 1.61 | 11 |  |  |
| MAIVLR | 1 | 1.76 | 11 |  |  |
| NP_005076.3 | nuclear pore complex protein Nup214 | |  |  |  |
| EIINQQRKR | 1 | 1.64 | 10 |  |  |
| QGLL | 1 | 1.61 | 11 |  |  |
| NP_055519.1 | centrosomal protein of 104 kDa |  |  |  |  |
| VLLTR | 1 | 1.58 | 10 |  |  |
| QHQASILEYLPPDDSNTR | 2 | 2.08 | 10 |  |  |
| NP_055895.1 | neurexin-2-beta isoform alpha-1 precursor | |  |  |  |
| NP_620060.1 | neurexin-2-beta isoform alpha-2 precursor | |  |  |  |
| VLLTR | 1 | 1.58 | 10 |  |  |
| AGGGAGSHSSAQR | 2 | 2.48 | 11 |  |  |
| FGLRAIVADPVTFKSR | 2 | 2.45 | 11 |  |  |
| NP_005695.3 | receptor-type tyrosine-protein phosphatase U isoform 3 precursor | | | |  |
| NP_573439.2 | receptor-type tyrosine-protein phosphatase U isoform 1 precursor | | | |  |
| NP_573438.3 | receptor-type tyrosine-protein phosphatase U isoform 2 precursor | | | |  |
| NP_001181930.1 | receptor-type tyrosine-protein phosphatase U isoform 4 precursor | | | |  |
| LVLTNPEGR | 2 | 2.00 | 11 |  |  |
| VLLTR | 1 | 1.58 | 10 |  |  |
| NP_001092093.1 | obscurin isoform b |  |  |  |  |
| NP_443075.2 | obscurin isoform a |  |  |  |  |
| DAVASAR | 1 | 1.81 | 11 |  |  |
| EAPAR | 1 | 1.66 | 11 |  |  |
| VVLTR | 1 | 1.58 | 12 |  |  |
| NP_057040.2 | 39S ribosomal protein L4, mitochondrial isoform a | | |  |  |
| NP_666499.1 | 39S ribosomal protein L4, mitochondrial isoform a | | |  |  |
| NP_666500.1 | 39S ribosomal protein L4, mitochondrial isoform b | | |  |  |
| ISYAKTKTR | 2 | 2.32 | 10 |  |  |
| RAGAR | 1 | 1.50 | 11 |  |  |
| NP_702914.1 | outer dense fiber protein 2 isoform 4 | |  |  |  |
| NP_702917.1 | outer dense fiber protein 2 isoform 5 | |  |  |  |
| NP_702910.1 | outer dense fiber protein 2 isoform 3 | |  |  |  |
| NP_702918.1 | outer dense fiber protein 2 isoform 7 | |  |  |  |
| NP_001229283.1 | outer dense fiber protein 2 isoform 8 | |  |  |  |
| NP_702915.1 | outer dense fiber protein 2 isoform 6 | |  |  |  |
| KNIDLTAIISDLR | 2 | 2.23 | 11 |  |  |
| RAGAR | 1 | 1.50 | 11 |  |  |
| NP_036433.2 | membrane-associated guanylate kinase, WW and PDZ domain-containing  protein 2 | | | | |
| RAGAR | 1 | 1.50 | 11 |  |  |
| AIELIKSGGR | 2 | 2.23 | 12 |  |  |
| NP_940927.2 | kinesin-like protein KIF7 |  |  |  |  |
| RAGAR | 1 | 1.50 | 11 |  |  |
| APGPATASAAAAMR | 1 | 1.56 | 10 |  |  |
| NP_000531.2 | ryanodine receptor 1 isoform 1 |  |  |  |  |
| NP_001036188.1 | ryanodine receptor 1 isoform 2 |  |  |  |  |
| LLYQQARLHTR | 2 | 2.14 | 11 |  |  |
| EAPAR | 1 | 1.66 | 11 |  |  |
| IEIMGASRR | 1 | 1.51 | 12 |  |  |
| ** cluster of 6 proteins |  |  |  |  |  |
| NP_001243211.1 | keratin, type II cytoskeletal 8 isoform 1 | |  |  |  |
| NP_001243222.1 | keratin, type II cytoskeletal 8 isoform 2 | |  |  |  |
| NP_775109.2 | keratin, type II cytoskeletal 6C |  |  |  |  |
| NP_056932.2 | keratin, type II cytoskeletal 2 oral | |  |  |  |
| NP_005546.2 | keratin, type II cytoskeletal 6B |  |  |  |  |
| NP_002264.1 | keratin, type II cytoskeletal 8 isoform 2 | |  |  |  |
| NP_005545.1 | keratin, type II cytoskeletal 6A |  |  |  |  |
| NKYEDEINKR | 2 | 3.27 | 12 |  |  |
| AQYEEIAQR | 2 | 3.53 | 12 |  |  |
| YEDEINKR | 2 | 2.07 | 11 |  |  |
| NP_006112.3 | keratin, type II cytoskeletal 1 |  |  |  |  |
| TLLEGEESR | 2 | 2.94 | 12 |  |  |
| NKYEDEINKR | 2 | 3.27 | 12 |  |  |
| YEDEINKR | 2 | 2.07 | 11 |  |  |
| NP_000415.2 | keratin, type II cytoskeletal 5 |  |  |  |  |
| NKYEDEINKR | 2 | 3.27 | 12 |  |  |
| YEDEINKR | 2 | 2.07 | 11 |  |  |
| GLGVGFGSGGGSSSSVKF VSTTSSSRKSFKS | 2 | 2.04 | 12 |  |  |
| ISISTSGGSFRNR | 1 | 2.22 | 12 |  |  |
| NP_002263.3 | keratin, type II cytoskeletal 4 |  |  |  |  |
| NLDLDSIIAEVR | 2 | 3.28 | 12 |  |  |
| AQYEEIAQR | 2 | 3.53 | 12 |  |  |
| NP_000414.2 | keratin, type II cytoskeletal 2 epidermal | |  |  |  |
| AQYEEIAQR | 2 | 3.53 | 12 |  |  |
| YEDEINKR | 2 | 2.07 | 11 |  |  |
| GFSSGSAVVSGGSR | 2 | 5.33 | 12 |  |  |
| GGGFGGGSSFGGGSGF SGGGFGGGGFGGGR | 2 | 6.76 | 11 |  |  |
| NP_004684.2 | keratin, type II cytoskeletal 75 |  |  |  |  |
| FSSVSVARSAAGSGGLGR | 2 | 2.02 | 12 |  |  |
| YEDEINKR | 2 | 2.07 | 11 |  |  |
| NP_001029249.1 | histone H4 |  |  |  |  |
| NP_003529.1 | histone H4 |  |  |  |  |
| NP_003530.1 | histone H4 |  |  |  |  |
| NP_003531.1 | histone H4 |  |  |  |  |
| NP_003532.1 | histone H4 |  |  |  |  |
| NP_003533.1 | histone H4 |  |  |  |  |
| NP_003534.1 | histone H4 |  |  |  |  |
| NP_003535.1 | histone H4 |  |  |  |  |
| NP_003536.1 | histone H4 |  |  |  |  |
| NP_003537.1 | histone H4 |  |  |  |  |
| NP_003486.1 | histone H4 |  |  |  |  |
| NP_003539.1 | histone H4 |  |  |  |  |
| NP_778224.1 | histone H4 |  |  |  |  |
| NP_068803.1 | histone H4 |  |  |  |  |
| DNIQGITKPAIR | 2 | 3.48 | 12 |  |  |
| ISGLIYEETR | 2 | 4.27 | 12 |  |  |
| TLYGFGG | 1 | 2.39 | 12 |  |  |
| NP_958842.1 | ras-related protein Rab-5C isoform a | |  |  |  |
| NP_001238968.1 | ras-related protein Rab-5C isoform b | |  |  |  |
| NP_004574.2 | ras-related protein Rab-5C isoform a | |  |  |  |
| GAQAAIVVYDITNTDTFAR | 2 | 4.66 | 11 |  |  |
| GVDLQENNPASR | 2 | 4.55 | 11 |  |  |
| NP_001137532.1 | NHS-like protein 1 isoform 2 |  |  |  |  |
| NP_065197.1 | NHS-like protein 1 isoform 1 |  |  |  |  |
| SLSFSGPRYGR | 2 | 2.12 | 5 |  |  |
| GALGAAEGCSLDGLAR | 2 | 2.58 | 6 |  |  |
| NP_006167.1 | neurogranin |  |  |  |  |
| NP_001119653.1 | neurogranin |  |  |  |  |
| GGAGGGPSGD | 1 | 1.95 | 10 |  |  |
| GRKGPGPGGPGGAGVAR | 2 | 2.24 | 9 |  |  |
| NP_808760.1 | histone H2A.J |  |  |  |  |
| NP_254280.1 | histone H2A type 3 |  |  |  |  |
| NP_619541.1 | histone H2A.V isoform 2 |  |  |  |  |
| NP_036544.1 | histone H2A.V isoform 1 |  |  |  |  |
| NP_001035807.1 | histone H2A type 2-A |  |  |  |  |
| NP_066409.1 | histone H2A type 1-D |  |  |  |  |
| NP_066408.1 | histone H2A type 1 |  |  |  |  |
| NP_066544.1 | histone cluster 1, H2aj |  |  |  |  |
| NP_066390.1 | histone H2A type 1-B/E |  |  |  |  |
| NP_003508.1 | histone H2A type 2-C |  |  |  |  |
| NP_542163.1 | histone H2A type 1-H |  |  |  |  |
| NP_003504.2 | histone H2A type 1-B/E |  |  |  |  |
| NP_734466.1 | histone H2A type 1-A |  |  |  |  |
| NP_003500.1 | histone H2A type 1 |  |  |  |  |
| NP_003501.1 | histone H2A type 1 |  |  |  |  |
| NP_003502.1 | histone H2A type 1 |  |  |  |  |
| NP_003503.1 | histone H2A type 1-C |  |  |  |  |
| NP_003505.1 | histone H2A type 1 |  |  |  |  |
| NP_003507.1 | histone H2A type 2-A |  |  |  |  |
| NP_002096.1 | histone H2A.x |  |  |  |  |
| NP_002097.1 | histone H2A.Z |  |  |  |  |
| HLQLAIR | 2 | 2.74 | 12 |  |  |
| AGLQFPVGR | 2 | 3.63 | 12 |  |  |
| NP_003371.2 | vimentin |  |  |  |  |
| SYVTTSTR | 2 | 2.20 | 12 |  |  |
| TYSLGSALRPSTSR | 2 | 3.06 | 12 |  |  |
| SLYASSPGGVYATR | 2 | 4.68 | 12 |  |  |
| NP_055455.3 | tubulin polyglutamylase TTLL4 |  |  |  |  |
| QKWIVKPPASAR | 2 | 2.16 | 9 |  |  |
| IYLFSDGLVR | 2 | 2.11 | 9 |  |  |
| NP_056057.2 | arf-GAP with Rho-GAP domain, ANK repeat and PH domain-containing  protein 1 isoform a | | | | |
| NP_001128662.1 | arf-GAP with Rho-GAP domain, ANK repeat and PH domain-containing  protein 1 isoform d | | | | |
| NP_001035207.1 | arf-GAP with Rho-GAP domain, ANK repeat and PH domain-containing  protein 1 isoform c | | | | |
| LAGLLR | 1 | 1.61 | 6 |  |  |
| DFERLGR | 2 | 2.05 | 6 |  |  |
| GLGAGVSKVR | 2 | 2.07 | 5 |  |  |
| NP_116277.2 | collagen alpha-1(XXVII) chain preproprotein | |  |  |  |
| SQKRKLQLGLQFLPGKTVVHLGSR | 2 | 2.21 | 12 |  |  |
| MGAGSARGAR | 2 | 2.33 | 11 |  |  |
| NP_001158886.1 | L-lactate dehydrogenase A chain isoform 3 | |  |  |  |
| NP_001158887.1 | L-lactate dehydrogenase A chain isoform 4 | |  |  |  |
| NP_001158888.1 | L-lactate dehydrogenase A chain isoform 5 | |  |  |  |
| NP_002291.1 | L-lactate dehydrogenase B chain |  |  |  |  |
| NP_001167568.1 | L-lactate dehydrogenase B chain |  |  |  |  |
| NP_005557.1 | L-lactate dehydrogenase A chain isoform 1 | |  |  |  |
| VIGSGCNLDSAR | 2 | 3.93 | 8 |  |  |
| LNLVQR | 1 | 2.14 | 8 |  |  |
| NP_001186143.1 | stathmin-2 isoform 1 |  |  |  |  |
| NP_008960.2 | stathmin-2 isoform 2 |  |  |  |  |
| ASGQAFELILKPPSPISEAPR | 2 | 3.31 | 11 |  |  |
| EANLAAIIER | 2 | 2.71 | 10 |  |  |
| NP_001106970.2 | MAM domain-containing glycosylphosphatidylinositol anchor protein 2 isoform 1 | | | | |
| WTKTAGSASDR | 1 | 1.87 | 10 |  |  |
| MSVWSAGLLR | 2 | 2.27 | 11 |  |  |
| NP_060282.1 | coiled-coil-helix-coiled-coil-helix domain-containing protein 3,  mitochondrial precursor | | | | |
| AAANEQLTR | 2 | 3.07 | 10 |  |  |
| LSENVIDR | 2 | 2.72 | 11 |  |  |
| NP_009194.2 | coatomer subunit epsilon isoform a | |  |  |  |
| NP_955476.1 | coatomer subunit epsilon isoform c | |  |  |  |
| DSIVAELDR | 2 | 2.96 | 9 |  |  |
| LVLQYAPSA | 1 | 1.78 | 9 |  |  |
| NP_543141.3 | probable G-protein coupled receptor 62 | |  |  |  |
| RAALRPPRPAR | 2 | 2.00 | 10 |  |  |
| LALGRLSR | 1 | 1.68 | 10 |  |  |
| NP_053733.2 | RNA-binding protein EWS isoform 1 | |  |  |  |
| NP_001156757.1 | RNA-binding protein EWS isoform 3 | |  |  |  |
| NP_001156758.1 | RNA-binding protein EWS isoform 4 | |  |  |  |
| NP_005234.1 | RNA-binding protein EWS isoform 2 | |  |  |  |
| GGFGGGR | 1 | 1.73 | 9 |  |  |
| GMDRGGFGGGR | 2 | 2.49 | 10 |  |  |
| NP_002461.2 | myosin-3 |  |  |  |  |
| QAAETLKHLR | 2 | 2.22 | 12 |  |  |
| RANLLQAEVEELR | 2 | 2.85 | 12 |  |  |
| NP_001135974.1 | relA-associated inhibitor |  |  |  |  |
| NP_006654.2 | relA-associated inhibitor |  |  |  |  |
| DLTLR | 1 | 1.78 | 12 |  |  |
| EGESVTVLR | 1 | 2.30 | 12 |  |  |
| NP_001129487.1 | annexin A2 isoform 2 |  |  |  |  |
| NP_001002857.1 | annexin A2 isoform 2 |  |  |  |  |
| NP_001002858.1 | annexin A2 isoform 1 |  |  |  |  |
| NP_004030.1 | annexin A2 isoform 2 |  |  |  |  |
| TNQELQEINR | 2 | 4.30 | 9 |  |  |
| AEDGSVIDYELIDQDAR | 2 | 5.24 | 9 |  |  |
| RAEDGSVIDYELIDQDAR | 2 | 5.50 | 8 |  |  |
| DLYDAGVKR | 2 | 2.47 | 8 |  |  |
| NP_055603.2 | tripartite motif-containing protein 14 | |  |  |  |
| NP_150088.1 | tripartite motif-containing protein 14 | |  |  |  |
| NP_150089.1 | tripartite motif-containing protein 14 | |  |  |  |
| MAGAATGSR | 1 | 1.54 | 12 |  |  |
| MAGAATGSRTPGR | 2 | 2.04 | 11 |  |  |
| NP_006151.3 | neurogenic differentiation factor 2 | |  |  |  |
| LAGAQCQAAGGLGGGAAHALR | 2 | 2.41 | 1 |  |  |
| NFLTEQGADGAGR | 2 | 2.14 | 2 |  |  |
| NP_775733.3 | papilin precursor |  |  |  |  |
| LRLDQNQPR | 2 | 2.69 | 4 |  |  |
| GAEGDLAPERLHAR | 2 | 2.45 | 3 |  |  |
| NP_001128527.1 | transketolase |  |  |  |  |
| NP_001055.1 | transketolase |  |  |  |  |
| DAIAQAVR | 1 | 3.11 | 5 |  |  |
| VLDPFTIKPLDR | 2 | 3.31 | 5 |  |  |
| MPSLPSYKVGDKIATR | 2 | 3.04 | 6 |  |  |
| KLILDSAR | 2 | 2.78 | 5 |  |  |
| NP_001036111.1 | trafficking kinesin-binding protein 1 isoform 1 | | |  |  |
| ENYLSERR | 1 | 1.60 | 9 |  |  |
| GLVPEGLPLR | 1 | 1.60 | 8 |  |  |
| NP_001185727.1 | activating signal cointegrator 1 complex subunit 1 isoform b | | | |  |
| NP_001185729.1 | activating signal cointegrator 1 complex subunit 1 isoform b | | | |  |
| FTVDSFGNYASCGQIDFS | 2 | 2.50 | 6 |  |  |
| HIVGKRGDTR | 2 | 2.19 | 6 |  |  |
| NP_001153467.1 | pro-neuregulin-1, membrane-bound isoform isoform HRG-beta1c | | | |  |
| NP_039250.2 | pro-neuregulin-1, membrane-bound isoform isoform HRG-beta1 | | | |  |
| NP_039251.2 | pro-neuregulin-1, membrane-bound isoform isoform HRG-beta2 | | | |  |
| NP_001153473.1 | pro-neuregulin-1, membrane-bound isoform isoform HRG-beta1d | | | |  |
| NP_039258.1 | pro-neuregulin-1, membrane-bound isoform isoform HRG-alpha | | | |  |
| NP_001153471.1 | pro-neuregulin-1, membrane-bound isoform isoform HRG-beta1b | | | |  |
| TKPNGHIANR | 1 | 1.59 | 1 |  |  |
| HSSPTGGPRGR | 1 | 1.79 | 2 |  |  |
| NP_004125.3 | stress-70 protein, mitochondrial precursor | |  |  |  |
| AQFEGIVTDLIR | 2 | 4.77 | 5 |  |  |
| TTPSVVAFTADGER | 2 | 4.53 | 5 |  |  |
| KDSETGENIR | 2 | 3.42 | 5 |  |  |
| NP_001129494.1 | nuclear factor of activated T-cells, cytoplasmic 4 isoform 1 | | | |  |
| NP_001185894.1 | nuclear factor of activated T-cells, cytoplasmic 4 isoform 3 | | | |  |
| NP_001185895.1 | nuclear factor of activated T-cells, cytoplasmic 4 isoform 4 | | | |  |
| NP_001185896.1 | nuclear factor of activated T-cells, cytoplasmic 4 isoform 5 | | | |  |
| NP_004545.2 | nuclear factor of activated T-cells, cytoplasmic 4 isoform 2 | | | |  |
| FGLGSPLPSPR | 2 | 2.34 | 12 |  |  |
| LGGPGGGAGGAGGGR | 1 | 1.67 | 12 |  |  |
| NP_006547.1 | phosphomevalonate kinase |  |  |  |  |
| MAPLGGAPR | 1 | 1.51 | 1 |  |  |
| KIVEGISQPIWLVSDTR | 2 | 2.45 | 1 |  |  |
| NP_620594.1 | A disintegrin and metalloproteinase with thrombospondin motifs 13  isoform 1 preproprotein | | | | |
| NP_620596.2 | A disintegrin and metalloproteinase with thrombospondin motifs 13  isoform 2 preproprotein | | | | |
| ILYCAR | 1 | 1.53 | 8 |  |  |
| QLLSLLSAGR | 2 | 2.39 | 7 |  |  |
| NP_005733.1 | protein disulfide-isomerase A6 precursor | |  |  |  |
| TGEAIVDAALSALR | 2 | 4.16 | 7 |  |  |
| DIVSR | 1 | 1.88 | 7 |  |  |
| GSTAPVGGGAFPTIVER | 2 | 5.29 | 7 |  |  |
| NP_001019820.1 | calnexin precursor |  |  |  |  |
| NP_001737.1 | calnexin precursor |  |  |  |  |
| EIEDPEDR | 2 | 2.45 | 4 |  |  |
| KIPNPDFFEDLEPFR | 2 | 3.72 | 4 |  |  |
| NP_001006933.1 | ribosomal protein S6 kinase alpha-2 isoform b | | |  |  |
| GGELLDR | 1 | 1.76 | 5 |  |  |
| EYLSPNQLSR | 2 | 2.09 | 4 |  |  |
| NP_001264.2 | chromodomain-helicase-DNA-binding protein 4 | | |  |  |
| RHDYWLLAGIINHGYAR | 2 | 2.69 | 10 |  |  |
| APEPTPQQVAQQQ | 2 | 2.06 | 10 |  |  |
| NP_004930.1 | ATP-dependent RNA helicase DDX1 | |  |  |  |
| FLICTDVAAR | 2 | 2.57 | 5 |  |  |
| ELLIIGGVAAR | 2 | 2.19 | 4 |  |  |
| NP_620591.3 | tetraspanin-32 |  |  |  |  |
| QELAAIQDVFLCCGKKSPFSR | 3 | 2.54 | 1 |  |  |
| EDCLQGIR | 1 | 1.89 | 2 |  |  |
| NP_005959.2 | heterogeneous nuclear ribonucleoprotein M isoform a | | |  |  |
| NP_112480.2 | heterogeneous nuclear ribonucleoprotein M isoform b | | |  |  |
| ADILEDKDGKSR | 2 | 2.12 | 10 |  |  |
| MGLAMGGGGGASFDR | 1 | 1.65 | 10 |  |  |
| NP_006022.3 | pericentrin |  |  |  |  |
| KDHVDELEPER | 2 | 2.02 | 8 |  |  |
| LLGLFGETLR | 2 | 2.26 | 7 |  |  |
| NP_002682.2 | DNA polymerase delta catalytic subunit | |  |  |  |
| ELNLAISR | 1 | 1.84 | 6 |  |  |
| FGVSSVAEAMALGR | 2 | 3.02 | 7 |  |  |
| NP_036339.1 | heterogeneous nuclear ribonucleoprotein H3 isoform a | | |  |  |
| NP_067676.2 | heterogeneous nuclear ribonucleoprotein H3 isoform b | | |  |  |
| ATENDIANFFSPLNPIR | 2 | 5.01 | 8 |  |  |
| YIELFLNSTPGGGSGMGGS GMGGYGR | 3 | 2.87 | 9 |  |  |
| NP_065208.2 | ankyrin-1 isoform 4 |  |  |  |  |
| NP_065210.2 | ankyrin-1 isoform 2 |  |  |  |  |
| NP_000028.3 | ankyrin-1 isoform 3 |  |  |  |  |
| NP_065209.2 | ankyrin-1 isoform 1 |  |  |  |  |
| EADAATSFLR | 2 | 2.15 | 6 |  |  |
| IGHTNMVKLLLENNANPN LATTAGHTPLHIAAR | 3 | 2.60 | 6 |  |  |
| NP_002503.1 | nucleoside diphosphate kinase B isoform a | |  |  |  |
| NP_000260.1 | nucleoside diphosphate kinase A isoform b | |  |  |  |
| NP_001018147.1 | nucleoside diphosphate kinase B isoform a | |  |  |  |
| NP_001018146.1 | NME1-NME2 protein |  |  |  |  |
| NP_001018148.1 | nucleoside diphosphate kinase B isoform a | |  |  |  |
| NP_001018149.1 | nucleoside diphosphate kinase B isoform a | |  |  |  |
| NP_937818.1 | nucleoside diphosphate kinase A isoform a | |  |  |  |
| GDFCIQVGR | 2 | 3.62 | 12 |  |  |
| TFIAIKPDGVQR | 2 | 3.88 | 12 |  |  |
| NP_115758.3 | conserved oligomeric Golgi complex subunit 8 | | |  |  |
| MNSLTLNR | 1 | 2.05 | 7 |  |  |
| FPEAQWR | 2 | 2.18 | 6 |  |  |
| NP_001116236.1 | protoporphyrinogen oxidase |  |  |  |  |
| NP_000300.1 | protoporphyrinogen oxidase |  |  |  |  |
| ELSIR | 1 | 1.87 | 4 |  |  |
| FLYVGGALHALPTGLR | 2 | 2.25 | 4 |  |  |
| NP_001004.2 | 40S ribosomal protein S9 |  |  |  |  |
| KTYVTPR | 2 | 2.34 | 12 |  |  |
| LFEGNALLR | 2 | 3.81 | 12 |  |  |
| NP_660286.1 | metalloendopeptidase OMA1, mitochondrial precursor | | |  |  |
| ITSKCTVWNDAFSR | 3 | 2.79 | 11 |  |  |
| NHVFFRFNSLSNWR | 2 | 2.10 | 10 |  |  |
| NP_056465.2 | TRAF3-interacting protein 1 isoform 1 | |  |  |  |
| NP_001132962.1 | TRAF3-interacting protein 1 isoform 2 | |  |  |  |
| RPPLTEKLLSKPPFR | 2 | 2.25 | 10 |  |  |
| KPREKDKDKEKAKENGGNR | 2 | 2.18 | 9 |  |  |
| NP_006301.3 | puromycin-sensitive aminopeptidase | |  |  |  |
| TQYSSAMLESLLPGIRDLSLPPVDR | 3 | 2.73 | 5 |  |  |
| LGLQNDLFSLAR | 2 | 4.16 | 4 |  |  |
| NP_079525.1 | uncharacterized protein KIAA1683 isoform b | |  |  |  |
| NP_001138777.1 | uncharacterized protein KIAA1683 isoform c | |  |  |  |
| TNKARAPETPLSR | 2 | 2.14 | 2 |  |  |
| FPCPVSLDAKCQPCLLTRTIR | 3 | 2.68 | 3 |  |  |
| NP_055553.3 | uncharacterized protein KIAA0195 | |  |  |  |
| GIKDDEHIVLEPGDLFPPF SPPPSPRGEVER | 3 | 2.97 | 9 |  |  |
| QEETISIIRLIEQAR | 2 | 3.38 | 8 |  |  |
| NP_000055.2 | complement C3 precursor |  |  |  |  |
| ASHLGLAR | 1 | 1.78 | 9 |  |  |
| NEQVEIR | 2 | 2.35 | 10 |  |  |
| NP_001355.2 | disks large homolog 2 isoform 2 |  |  |  |  |
| NP_001136174.1 | disks large homolog 2 isoform 4 |  |  |  |  |
| NP_001136171.1 | disks large homolog 2 isoform 1 |  |  |  |  |
| NP_001136172.1 | disks large homolog 2 isoform 3 |  |  |  |  |
| NP_001193698.1 | disks large homolog 2 isoform 5 |  |  |  |  |
| DYHFVISR | 1 | 1.63 | 11 |  |  |
| MMNHSMSSGSGSLR | 2 | 2.37 | 10 |  |  |
| NP_061992.2 | protocadherin beta-9 precursor |  |  |  |  |
| ENNSPALHIGSVSATDR | 2 | 2.26 | 5 |  |  |
| DINDHSPVFR | 1 | 1.81 | 5 |  |  |
| NP_542418.1 | regulator of nonsense transcripts 3A isoform hUpf3pdelta | | | |  |
| NP_075387.1 | regulator of nonsense transcripts 3A isoform hUpf3p | | |  |  |
| KERLANKDRPALQLYDPGAR | 2 | 2.13 | 3 |  |  |
| TALSKVVIRR | 2 | 2.92 | 3 |  |  |
| GPSGREKLSALEVQFHR | 2 | 2.40 | 3 |  |  |
| NP_006079.1 | tubulin beta-4B chain |  |  |  |  |
| NP_821133.1 | tubulin beta chain |  |  |  |  |
| NP_006078.2 | tubulin beta-4A chain |  |  |  |  |
| YLTVAAVFR | 2 | 2.83 | 7 |  |  |
| FPGQLNADLR | 2 | 2.84 | 7 |  |  |
| NP_942094.2 | breast carcinoma-amplified sequence 4 isoform b | | |  |  |
| NP_001010974.1 | breast carcinoma-amplified sequence 4 isoform c | | |  |  |
| NP_060313.3 | breast carcinoma-amplified sequence 4 isoform a | | |  |  |
| GGGAPR | 1 | 1.63 | 1 |  |  |
| MQRTGGGAPRPGR | 2 | 2.46 | 2 |  |  |
| NP_056667.2 | LIM domain only protein 7 isoform 2 | |  |  |  |
| RIPAQKKEVPLSGAPDR | 2 | 2.61 | 12 |  |  |
| LLQEKYQR | 1 | 1.52 | 11 |  |  |
| NP_001014985.2 | glycolipid transfer protein domain-containing protein 2 precursor | | | |  |
| FHASLKPEGDVGLSPYLAGWR | 3 | 2.87 | 8 |  |  |
| TLLLLHR | 2 | 2.17 | 9 |  |  |
| NP_055707.3 | protein SCAF8 |  |  |  |  |
| FPPIETR | 2 | 2.27 | 7 |  |  |
| DVVGRPIDPR | 2 | 2.11 | 6 |  |  |
| NP_001159450.1 | prominin-2 precursor |  |  |  |  |
| NP_653308.2 | prominin-2 precursor |  |  |  |  |
| NP_001159449.1 | prominin-2 precursor |  |  |  |  |
| VRAPGLLDSLYGTVR | 2 | 2.29 | 9 |  |  |
| WLAPRVR | 2 | 2.02 | 8 |  |  |
| NP_002128.1 | heterogeneous nuclear ribonucleoproteins A2/B1 isoform A2 | | | |  |
| NP_112533.1 | heterogeneous nuclear ribonucleoproteins A2/B1 isoform B1 | | | |  |
| GGGGNFGPGPGSNFR | 2 | 3.83 | 8 |  |  |
| QEMQEVQSSR | 2 | 3.13 | 8 |  |  |
| GGNFGFGDSR | 2 | 3.67 | 8 |  |  |
| NP_000823.4 | glutamate [NMDA] receptor subunit zeta-1 isoform NR1-1 precursor | | | |  |
| NP_067544.1 | glutamate [NMDA] receptor subunit zeta-1 isoform NR1-2 precursor | | | |  |
| NP_015566.1 | glutamate [NMDA] receptor subunit zeta-1 isoform NR1-3 precursor | | | |  |
| NP_001172019.1 | glutamate [NMDA] receptor subunit zeta-1 isoform NR1-4 precursor | | | |  |
| NP_001172020.1 | glutamate [NMDA] receptor subunit zeta-1 isoform 5 precursor | | | |  |
| EISGNALR | 2 | 2.04 | 4 |  |  |
| AAAMLNMTGSGYVWLVGER | 2 | 2.59 | 4 |  |  |
| NP_001087240.1 | thioredoxin reductase 1, cytoplasmic isoform 3 | | |  |  |
| FLIATGERPR | 2 | 3.08 | 6 |  |  |
| MGCAEGKAVAAAAPTEL QTKGKNGDGR | 2 | 2.35 | 7 |  |  |
| NP_055625.4 | centrosome-associated protein 350 | |  |  |  |
| LQEANKAAR | 1 | 2.27 | 6 |  |  |
| QHLPDFVKQLRTR | 2 | 2.00 | 6 |  |  |
| NP_001171577.1 | zinc finger protein 185 isoform 1 |  |  |  |  |
| NP_001171578.1 | zinc finger protein 185 isoform 2 |  |  |  |  |
| NP_001171579.1 | zinc finger protein 185 isoform 3 |  |  |  |  |
| NP_001171580.1 | zinc finger protein 185 isoform 5 |  |  |  |  |
| NP_001171581.1 | zinc finger protein 185 isoform 6 |  |  |  |  |
| NP_009081.2 | zinc finger protein 185 isoform 4 |  |  |  |  |
| QSSPSGSEQLVRR | 2 | 2.38 | 1 |  |  |
| MSISALGGR | 2 | 2.37 | 1 |  |  |
| NP_071387.1 | phosphorylated CTD-interacting factor 1 | |  |  |  |
| MANENHGSPR | 2 | 2.05 | 3 |  |  |
| EVELLR | 1 | 2.23 | 4 |  |  |
| NP_116262.2 | ubiquitin-associated and SH3 domain-containing protein B | | | |  |
| ARAQKALASTGGR | 2 | 2.14 | 11 |  |  |
| ETLLQE | 1 | 2.09 | 10 |  |  |
| NP_001013860.1 | leucine-rich repeat-containing protein 16C | |  |  |  |
| NEDGQLRPRPLSAGR | 2 | 2.60 | 11 |  |  |
| QVGEVCR | 2 | 2.13 | 12 |  |  |
| NP_060876.5 | mucin-4 isoform a precursor |  |  |  |  |
| NP_612154.2 | mucin-4 isoform e precursor |  |  |  |  |
| NP_004523.3 | mucin-4 isoform d precursor |  |  |  |  |
| GCSGAR | 1 | 2.20 | 9 |  |  |
| KMTNNGGYKAR | 2 | 2.09 | 9 |  |  |
| NP_005304.3 | protein disulfide-isomerase A3 precursor | |  |  |  |
| LAPEYEAAATR | 2 | 3.60 | 6 |  |  |
| ELSDFISYLQR | 2 | 3.84 | 6 |  |  |
| NP_056507.2 | C3 and PZP-like alpha-2-macroglobulin domain-containing protein 8 | | | |  |
| APGGGAR | 1 | 1.61 | 4 |  |  |
| YEVAGR | 1 | 1.51 | 3 |  |  |
| NP_001166095.1 | neogenin isoform 3 precursor |  |  |  |  |
| NP_001166094.1 | neogenin isoform 2 precursor |  |  |  |  |
| NP_002490.2 | neogenin isoform 1 precursor |  |  |  |  |
| QPLLLDDR | 2 | 2.09 | 4 |  |  |
| SPLVR | 1 | 1.70 | 5 |  |  |
| NP_001035088.2 | protein FAM83G |  |  |  |  |
| DSSDIGWVLELPKEEAPQN GTDHRLPR | 3 | 2.72 | 6 |  |  |
| TGGSQWASSDSKRR | 2 | 2.05 | 5 |  |  |
| NP_057692.1 | armadillo repeat-containing X-linked protein 1 | | |  |  |
| NP_055527.1 | histone-lysine N-methyltransferase SETD1A | |  |  |  |
| NP_849158.2 | mitochondrial cardiolipin hydrolase | |  |  |  |
| RAGGR | 1 | 1.61 | 8 |  |  |
| SRAGGR | 1 | 1.54 | 8 |  |  |
| NP_005289.2 | probable G-protein coupled receptor 25 | |  |  |  |
| ISSASSLSR | 2 | 2.08 | 12 |  |  |
| ARALDGACGR | 1 | 2.01 | 12 |  |  |
| NP_835455.1 | pancreas transcription factor 1 subunit alpha | | |  |  |
| GGGAGGCGGPGGGGR | 2 | 2.18 | 4 |  |  |
| GLSGAAAAAARR | 2 | 2.02 | 4 |  |  |
| NP_072045.1 | 40S ribosomal protein S18 |  |  |  |  |
| VLNTNIDGR | 2 | 3.37 | 12 |  |  |
| YAHVVLR | 2 | 2.07 | 12 |  |  |
| AGELTEDEVER | 2 | 4.09 | 12 |  |  |
| NP_001177893.1 | zinc finger MYM-type protein 2 |  |  |  |  |
| NP_001177894.1 | zinc finger MYM-type protein 2 |  |  |  |  |
| NP_932072.1 | zinc finger MYM-type protein 2 |  |  |  |  |
| NP_003444.1 | zinc finger MYM-type protein 2 |  |  |  |  |
| VEEDYLWR | 2 | 2.05 | 7 |  |  |
| YQVSSLCGTDNEDKITTGKR | 2 | 2.08 | 7 |  |  |
| NP_033611.1 | type-1 angiotensin II receptor |  |  |  |  |
| NP_004826.2 | type-1 angiotensin II receptor |  |  |  |  |
| NP_114038.1 | type-1 angiotensin II receptor |  |  |  |  |
| NP_114438.1 | type-1 angiotensin II receptor |  |  |  |  |
| NP_000676.1 | type-1 angiotensin II receptor |  |  |  |  |
| YLAIVHPMKSRLR | 2 | 2.25 | 8 |  |  |
| IQDDCPKAGR | 2 | 2.20 | 8 |  |  |
| NP_079524.2 | SRC kinase signaling inhibitor 1 |  |  |  |  |
| GSDELTVPR | 1 | 1.61 | 10 |  |  |
| TLGGGGGGGSGGR | 1 | 1.89 | 10 |  |  |
| NP_000219.2 | laminin subunit beta-3 precursor | |  |  |  |
| NP_001017402.1 | laminin subunit beta-3 precursor | |  |  |  |
| NP_001121113.1 | laminin subunit beta-3 precursor | |  |  |  |
| MEELRHQAR | 1 | 1.84 | 5 |  |  |
| SFNGLLTMYQR | 2 | 2.00 | 5 |  |  |
| NP_000594.2 | nitric oxide synthase, endothelial isoform 1 | |  |  |  |
| DIFSPKR | 1 | 1.56 | 4 |  |  |
| GFWQER | 2 | 2.06 | 4 |  |  |
| NP_001395.1 | elongation factor 1-gamma |  |  |  |  |
| TFLVGER | 2 | 2.69 | 7 |  |  |
| KLDPGSEETQTLVR | 2 | 4.37 | 7 |  |  |
| NP_689600.2 | BTB/POZ domain-containing protein KCTD18 | |  |  |  |
| KAAQRSAPSR | 1 | 1.91 | 6 |  |  |
| LNVGGCIYTAR | 2 | 2.09 | 6 |  |  |
| NP_004915.2 | alpha-actinin-4 |  |  |  |  |
| NP_001123476.1 | alpha-actinin-1 isoform a |  |  |  |  |
| NP_001123477.1 | alpha-actinin-1 isoform c |  |  |  |  |
| NP_001093.1 | alpha-actinin-1 isoform b |  |  |  |  |
| ELPPDQAEYCIAR | 2 | 3.29 | 4 |  |  |
| VGWEQLLTTIAR | 2 | 4.84 | 4 |  |  |
| ASFNHFDR | 2 | 2.22 | 4 |  |  |
| TINEVENQILTR | 2 | 4.64 | 4 |  |  |
| NP_003346.2 | mitochondrial uncoupling protein 2 | |  |  |  |
| AGGGRR | 1 | 1.58 | 11 |  |  |
| FQAQARAGGGR | 2 | 2.18 | 11 |  |  |
| NP_001017992.1 | beta-actin-like protein 2 |  |  |  |  |
| NP_001186822.1 | actin, gamma-enteric smooth muscle isoform 2 precursor | | |  |  |
| NP_001186883.1 | actin, cytoplasmic 2 |  |  |  |  |
| NP_001091.1 | actin, alpha skeletal muscle |  |  |  |  |
| NP_001604.1 | actin, aortic smooth muscle |  |  |  |  |
| NP_001092.1 | actin, cytoplasmic 1 |  |  |  |  |
| NP_001605.1 | actin, cytoplasmic 2 |  |  |  |  |
| NP_001606.1 | actin, gamma-enteric smooth muscle isoform 1 precursor | | |  |  |
| NP_001135417.1 | actin, aortic smooth muscle |  |  |  |  |
| NP_005150.1 | actin, alpha cardiac muscle 1 proprotein | |  |  |  |
| SYELPDGQVITIGNER | 2 | 4.88 | 10 |  |  |
| DLTDYLMKILTER | 2 | 2.12 | 10 |  |  |
| NP_079109.2 | zinc finger protein 671 |  |  |  |  |
| MLSPVSR | 1 | 1.68 | 2 |  |  |
| KQTLVLHQRVHAGEKL | 2 | 2.01 | 1 |  |  |
| NP_005306.1 | homeobox protein goosecoid-2 |  |  |  |  |
| VEVWFKNR | 2 | 2.48 | 3 |  |  |
| MAAAAGGAASR | 2 | 2.47 | 3 |  |  |
| NP_001171689.1 | clathrin light chain A isoform d |  |  |  |  |
| NP_001171690.1 | clathrin light chain A isoform e |  |  |  |  |
| NP_001070145.1 | clathrin light chain A isoform c |  |  |  |  |
| NP_001824.1 | clathrin light chain A isoform a |  |  |  |  |
| NP_009027.1 | clathrin light chain A isoform b |  |  |  |  |
| LEALDANSR | 2 | 3.70 | 9 |  |  |
| LQSEPESIR | 2 | 2.76 | 9 |  |  |
| AAEEAFVNDIDESSPGTEWER | 2 | 5.24 | 9 |  |  |
| NP_001153602.1 | potassium voltage-gated channel subfamily KQT member 5 isoform 2 | | | |  |
| NP_001153604.1 | potassium voltage-gated channel subfamily KQT member 5 isoform 3 | | | |  |
| NP_062816.2 | potassium voltage-gated channel subfamily KQT member 5 isoform 1 | | | |  |
| NP_001153605.1 | potassium voltage-gated channel subfamily KQT member 5 isoform 4 | | | |  |
| NP_001153606.1 | potassium voltage-gated channel subfamily KQT member 5 isoform 5 | | | |  |
| EAAFASDSLR | 2 | 2.13 | 4 |  |  |
| AATLGGGGGGLRESR | 2 | 2.24 | 5 |  |  |
| NP_001001937.1 | ATP synthase subunit alpha, mitochondrial precursor | | |  |  |
| NP_004037.1 | ATP synthase subunit alpha, mitochondrial precursor | | |  |  |
| VGSAAQTR | 1 | 1.81 | 10 |  |  |
| RAGLVSR | 1 | 1.88 | 11 |  |  |
| NP_001615.1 | absent in melanoma 1 protein |  |  |  |  |
| ELGRAAGAPGASDADGLKPR | 2 | 2.21 | 3 |  |  |
| FIDVEFSEPTIILFER | 2 | 2.02 | 3 |  |  |
| NP_000581.1 | interleukin-9 precursor |  |  |  |  |
| YPLIFSR | 1 | 1.94 | 5 |  |  |
| LSQMTNTTMQTR | 2 | 2.26 | 4 |  |  |
| NP_001407.1 | eukaryotic initiation factor 4A-I isoform 1 | |  |  |  |
| NP_001958.2 | eukaryotic initiation factor 4A-II |  |  |  |  |
| NP_001191439.1 | eukaryotic initiation factor 4A-I isoform 2 | |  |  |  |
| GIYAYGFEKPSAIQQR | 2 | 5.21 | 7 |  |  |
| QFYINVER | 2 | 2.11 | 7 |  |  |
| VLITTDLLAR | 2 | 4.16 | 7 |  |  |
| GIDVQQVSLVINYDLPTNR | 2 | 5.88 | 7 |  |  |
| NP_061859.3 | SH3 domain and tetratricopeptide repeats-containing protein 1 | | | |  |
| GSLAGSLR | 1 | 1.89 | 8 |  |  |
| ALPLAVTTGNR | 2 | 2.31 | 8 |  |  |
| NP_057688.2 | lysine-specific demethylase 3B |  |  |  |  |
| MADAAASPVGKR | 2 | 2.10 | 9 |  |  |
| DGRLNLASR | 2 | 2.43 | 9 |  |  |
| NP_001694.2 | brain-specific angiogenesis inhibitor 2 precursor | | |  |  |
| MLAGEGMSQVVRSLQELLAR | 2 | 2.50 | 6 |  |  |
| YLYLSLR | 2 | 2.17 | 5 |  |  |
| NP_001447.2 | filamin-A isoform 1 |  |  |  |  |
| NP_001104026.1 | filamin-A isoform 2 |  |  |  |  |
| EATTEFSVDAR | 2 | 3.54 | 1 |  |  |
| AEAGVPAEFSIWTR | 2 | 4.33 | 1 |  |  |
| IANLQTDLSDGLR | 2 | 4.75 | 1 |  |  |
| VANPSGNLTETYVQDR | 2 | 5.87 | 1 |  |  |
| NP_056986.2 | E3 ubiquitin-protein ligase UBR5 | |  |  |  |
| EEASLR | 1 | 1.53 | 11 |  |  |
| MTAREEASLR | 2 | 2.52 | 11 |  |  |
| NP_002789.1 | proteasome subunit beta type-6 |  |  |  |  |
| DGSSGGVIR | 2 | 2.67 | 11 |  |  |
| LAAIAESGVER | 2 | 4.38 | 11 |  |  |
| TTTGSYIANR | 2 | 3.43 | 11 |  |  |
| NP_001017963.2 | heat shock protein HSP 90-alpha isoform 1 | |  |  |  |
| NP_031381.2 | heat shock protein HSP 90-beta |  |  |  |  |
| NP_005339.3 | heat shock protein HSP 90-alpha isoform 2 | |  |  |  |
| GTKVILHLKEDQTEYLEER | 2 | 2.09 | 6 |  |  |
| GVVDSEDLPLNISR | 2 | 4.22 | 5 |  |  |
| NP_689805.3 | nesprin-3 |  |  |  |  |
| TRKSKLQELEAR | 2 | 2.24 | 10 |  |  |
| SLEDLVDRCR | 2 | 2.38 | 9 |  |  |
| NP_001279.2 | chloride intracellular channel protein 1 | |  |  |  |
| GFTIPEAFR | 2 | 2.66 | 10 |  |  |
| YLSNAYAR | 2 | 2.39 | 9 |  |  |
| NP_000341.2 | retinal-specific ATP-binding cassette transporter | | |  |  |
| ENVNPRHPCLGPR | 2 | 2.33 | 8 |  |  |
| FIIFSQR | 1 | 1.76 | 9 |  |  |
| NP_066022.2 | spectrin beta chain, brain 3 isoform sigma1 | |  |  |  |
| YEELAAELLAWIHR | 2 | 2.08 | 12 |  |  |
| FSSLRR | 1 | 1.59 | 12 |  |  |
| NP_068767.3 | brevican core protein isoform 1 precursor | |  |  |  |
| YEVDTVLR | 2 | 2.40 | 10 |  |  |
| EAEVLVAR | 2 | 2.04 | 11 |  |  |
| NP_001419.1 | alpha-enolase isoform 1 |  |  |  |  |
| AAVPSGASTGIYEALELR | 2 | 5.66 | 7 |  |  |
| EIFDSR | 1 | 1.58 | 7 |  |  |
| NP_001135745.1 | CLIP-associating protein 1 isoform 2 | |  |  |  |
| NP_001135746.1 | CLIP-associating protein 1 isoform 3 | |  |  |  |
| NP_001193980.1 | CLIP-associating protein 1 isoform 4 | |  |  |  |
| NP_056097.1 | CLIP-associating protein 1 isoform 1 | |  |  |  |
| KGALLELLKITR | 2 | 2.48 | 11 |  |  |
| ASTVSTKSVSTTGSLQRSR | 2 | 2.52 | 11 |  |  |
| NP_000964.1 | 60S ribosomal protein L8 |  |  |  |  |
| NP_150644.1 | 60S ribosomal protein L8 |  |  |  |  |
| AVVGVVAGGGR | 2 | 4.10 | 9 |  |  |
| AVDFAER | 2 | 2.53 | 9 |  |  |
| NP_056003.1 | TBC1 domain family member 12 |  |  |  |  |
| DCRDLEEAR | 2 | 2.11 | 10 |  |  |
| DLEEAR | 1 | 2.26 | 11 |  |  |
| NP_000217.2 | keratin, type I cytoskeletal 9 |  |  |  |  |
| FSSSSGYGGGSSR | 2 | 4.40 | 11 |  |  |
| SGGGGGGGLGSGGSIR | 2 | 5.23 | 11 |  |  |
| NP_000532.2 | S-arrestin |  |  |  |  |
| YGQEDIDVIGLTFRR | 2 | 2.20 | 6 |  |  |
| DKSVTIYLGNR | 2 | 2.23 | 5 |  |  |
| NP_006715.2 | mitogen-activated protein kinase kinase kinase 4 isoform b | | | |  |
| NP_005913.2 | mitogen-activated protein kinase kinase kinase 4 isoform a | | | |  |
| SVIEISR | 2 | 2.14 | 11 |  |  |
| RSWELR | 1 | 1.67 | 12 |  |  |
| NP_002286.2 | 40S ribosomal protein SA |  |  |  |  |
| NP_001012321.1 | 40S ribosomal protein SA |  |  |  |  |
| AIVAIENPADVSVISSR | 2 | 5.99 | 8 |  |  |
| LLVVTDPR | 2 | 3.38 | 8 |  |  |
| NP_005466.1 | SH2B adapter protein 3 |  |  |  |  |
| EPPPEALKEAVLR | 2 | 2.01 | 5 |  |  |
| VLELFDPPKSSRPKLQAACS SIQEVRWCTR | 3 | 2.61 | 4 |  |  |
| NP_071386.3 | zinc finger protein 667 |  |  |  |  |
| LSTLILHLR | 2 | 2.08 | 10 |  |  |
| NLVSLGLSFR | 1 | 2.05 | 9 |  |  |
| NP_001138303.1 | prohibitin-2 isoform 1 |  |  |  |  |
| NP_009204.1 | prohibitin-2 isoform 2 |  |  |  |  |
| AQVSLLIR | 2 | 3.11 | 9 |  |  |
| LGLDYEER | 2 | 3.30 | 9 |  |  |
| ESVFTVEGGHR | 2 | 3.50 | 9 |  |  |
| NP_062556.2 | RNA binding motif protein, X-linked-like-1 | |  |  |  |
| NP_001156008.1 | RNA binding motif protein, X-linked-like-1 | |  |  |  |
| NP_002130.2 | RNA-binding motif protein, X chromosome isoform 1 | | |  |  |
| SDLYSSGR | 2 | 2.59 | 7 |  |  |
| GAPRGGGR | 1 | 1.52 | 7 |  |  |
| REPLPSR | 2 | 2.55 | 7 |  |  |
| GPPPSYGGSSR | 2 | 2.85 | 7 |  |  |
| NP_001124197.1 | proteasome subunit beta type-5 isoform 2 | |  |  |  |
| NP_002788.1 | proteasome subunit beta type-5 isoform 1 | |  |  |  |
| DAYSGGAVNLYHVR | 2 | 4.63 | 11 |  |  |
| GYSYDLEVEQAYDLAR | 2 | 4.02 | 12 |  |  |
| NP_001143.2 | ADP/ATP translocase 2 |  |  |  |  |
| NP_001627.2 | ADP/ATP translocase 3 |  |  |  |  |
| NP_001142.2 | ADP/ATP translocase 1 |  |  |  |  |
| YFAGNLASGGAAGATS LCFVYPLDFAR | 2 | 2.31 | 10 |  |  |
| GNLANVIR | 2 | 2.47 | 10 |  |  |
| NP_919262.2 | serine/arginine repetitive matrix protein 4 | |  |  |  |
| DEKRHKKQSR | 1 | 1.52 | 4 |  |  |
| GTFKAVATPRPESIIVASITAR | 2 | 2.35 | 4 |  |  |
| NP_003742.2 | eukaryotic translation initiation factor 3 subunit B | | |  |  |
| NP_001032360.1 | eukaryotic translation initiation factor 3 subunit B | | |  |  |
| AQAVSEDAGGNEGR | 2 | 5.64 | 3 |  |  |
| DQYSVIFESGDR | 2 | 3.68 | 3 |  |  |
| NP_001008.1 | 40S ribosomal protein S13 |  |  |  |  |
| LILIESR | 2 | 2.18 | 12 |  |  |
| DSHGVAQVR | 2 | 2.78 | 12 |  |  |
| NP_001976.1 | electron transfer flavoprotein subunit beta isoform 1 | | |  |  |
| NP_001014763.1 | electron transfer flavoprotein subunit beta isoform 2 | | |  |  |
| EIDGGLETLR | 2 | 3.51 | 10 |  |  |
| LGPLQVAR | 2 | 2.89 | 10 |  |  |
| NP_075392.2 | ankyrin repeat domain-containing protein 57 | | |  |  |
| AHFKELVNAVATVR | 2 | 2.28 | 6 |  |  |
| FLAERGGR | 2 | 2.04 | 7 |  |  |
| NP_001175.2 | serine/threonine-protein kinase ATR | |  |  |  |
| AKEPILALR | 2 | 2.41 | 8 |  |  |
| EMALNTLSEIANVFDFPDLNR | 2 | 2.09 | 8 |  |  |
| NP_001012302.2 | anoctamin-9 |  |  |  |  |
| LDAIKMVWLQR | 2 | 2.02 | 6 |  |  |
| FFTLQFFTHFSSLIYIAFILGRI NGHPGKSTR | 3 | 2.76 | 7 |  |  |
| NP_001155855.1 | rho guanine nucleotide exchange factor 2 isoform 1 | | |  |  |
| NP_001155856.1 | rho guanine nucleotide exchange factor 2 isoform 2 | | |  |  |
| MSRIESLTR | 2 | 2.15 | 10 |  |  |
| IESLTRAR | 2 | 2.21 | 10 |  |  |
| NP_612202.1 | NACHT, LRR and PYD domains-containing protein 6 | | |  |  |
| FLFGLLSAER | 1 | 1.96 | 1 |  |  |
| TAAGGVGTLLR | 2 | 2.42 | 2 |  |  |
| NP_055392.1 | bromodomain-containing protein 1 | |  |  |  |
| KTAYCDVHTPPGCTR | 2 | 2.27 | 8 |  |  |
| LTPLTVLLR | 2 | 2.13 | 8 |  |  |
| NP_079388.3 | zinc finger protein ZXDC isoform 1 | |  |  |  |
| NP_001035743.1 | zinc finger protein ZXDC isoform 2 | |  |  |  |
| YKLKR | 1 | 1.64 | 8 |  |  |
| CPVSTCNR | 2 | 2.20 | 8 |  |  |
| NP_079411.2 | FH1/FH2 domain-containing protein 3 | |  |  |  |
| IIILKIVHRR | 2 | 3.06 | 9 |  |  |
| ISTLQANSQTQDESVR | 2 | 2.68 | 8 |  |  |
| NP_004230.2 | thyroid receptor-interacting protein 11 | |  |  |  |
| TDVNPFLAPR | 2 | 2.12 | 1 |  |  |
| ETIQNLSR | 1 | 1.65 | 1 |  |  |
| NP_000393.4 | glucose-6-phosphate 1-dehydrogenase isoform a | | |  |  |
| NP_001035810.1 | glucose-6-phosphate 1-dehydrogenase isoform b | | |  |  |
| EDQIYR | 2 | 2.14 | 6 |  |  |
| LSNHISSLFR | 2 | 3.73 | 6 |  |  |
| DGLLPENTFIVGYAR | 2 | 4.41 | 7 |  |  |
| IFGPIWNR | 2 | 3.01 | 6 |  |  |
| GGYFDEFGIIR | 2 | 4.16 | 6 |  |  |
| NP_003323.1 | TYRO protein tyrosine kinase-binding protein isoform 1 precursor | | | |  |
| NP_001166985.1 | TYRO protein tyrosine kinase-binding protein isoform 3 precursor | | | |  |
| GRGAAEAATR | 2 | 2.44 | 3 |  |  |
| GAAEAATR | 1 | 1.51 | 2 |  |  |
| NP_005057.1 | splicing factor, proline- and glutamine-rich | |  |  |  |
| FATHAAALSVR | 2 | 3.44 | 4 |  |  |
| FGQGGAGPVGGQGPR | 2 | 4.68 | 4 |  |  |
| FAQHGTFEYEYSQR | 3 | 3.82 | 4 |  |  |
| AVVIVDDR | 2 | 2.69 | 4 |  |  |
| NP_115735.2 | uncharacterized protein C3orf26 isoform 1 | |  |  |  |
| NP_001161396.1 | uncharacterized protein C3orf26 isoform 2 | |  |  |  |
| KENTTKTRKR | 2 | 2.14 | 12 |  |  |
| KNHSEKKSVLMLIICSSAVR | 2 | 2.14 | 12 |  |  |
| NP_066576.1 | myotubularin-related protein 3 isoform c | |  |  |  |
| NP_694690.1 | myotubularin-related protein 3 isoform a | |  |  |  |
| NP_694691.1 | myotubularin-related protein 3 isoform b | |  |  |  |
| SCLVNSGKDR | 2 | 2.05 | 5 |  |  |
| FKNEVER | 2 | 2.33 | 5 |  |  |
| NP_001092879.1 | EH domain-binding protein 1-like protein 1 | |  |  |  |
| ETQAQACPQEGTEAHGAR | 2 | 2.36 | 6 |  |  |
| EAPPR | 1 | 1.65 | 5 |  |  |
| NP_001677.2 | ATP synthase subunit beta, mitochondrial precursor | | |  |  |
| VALTGLTVAEYFR | 2 | 5.53 | 6 |  |  |
| FTQAGSEVSALLGR | 2 | 5.39 | 6 |  |  |
| DQEGQDVLLFIDNIFR | 2 | 6.17 | 6 |  |  |
| AIAELGIYPAVDPLDSTSR | 2 | 5.01 | 6 |  |  |
| NP_003325.2 | ubiquitin-like modifier-activating enzyme 1 | |  |  |  |
| NP_695012.1 | ubiquitin-like modifier-activating enzyme 1 | |  |  |  |
| QMNPHIR | 2 | 2.14 | 3 |  |  |
| LAGTQPLEVLEAVQR | 2 | 3.72 | 2 |  |  |
| NP_003837.1 | peroxisomal membrane protein 11B isoform 1 | | |  |  |
| NP_001171724.1 | peroxisomal membrane protein 11B isoform 2 | | |  |  |
| LQVLLLAR | 2 | 2.00 | 5 |  |  |
| YYLFSLIMNLSR | 2 | 2.50 | 5 |  |  |
| NP_003728.1 | protocadherin-16 precursor |  |  |  |  |
| IDAHSGDVCTTRTLDR | 2 | 2.09 | 11 |  |  |
| TEIAR | 1 | 1.75 | 11 |  |  |
| NP_036205.1 | T-complex protein 1 subunit epsilon | |  |  |  |
| IADGYEQAAR | 2 | 3.88 | 6 |  |  |
| WVGGPEIELIAIATGGR | 2 | 4.93 | 6 |  |  |
| NP_057302.1 | DNA polymerase kappa |  |  |  |  |
| SMSVER | 1 | 2.02 | 4 |  |  |
| FAMELEQSR | 1 | 2.31 | 3 |  |  |
| NP_004697.2 | rho guanine nucleotide exchange factor 1 isoform 2 | | |  |  |
| NP_945328.1 | rho guanine nucleotide exchange factor 1 isoform 3 | | |  |  |
| NP_945353.1 | rho guanine nucleotide exchange factor 1 isoform 1 | | |  |  |
| LLLKSHSR | 2 | 2.18 | 12 |  |  |
| KGGVGMPSR | 2 | 2.02 | 11 |  |  |
| NP_004583.2 | splicing factor, suppressor of white-apricot homolog | | |  |  |
| LAAAAR | 1 | 1.72 | 9 |  |  |
| KSGAKEEAGPGGAGGGGSR | 2 | 2.08 | 8 |  |  |
| NP_001295.2 | carboxypeptidase D isoform 1 precursor | |  |  |  |
| EVVGR | 1 | 1.57 | 8 |  |  |
| EAAAAGLPGLAR | 2 | 2.08 | 7 |  |  |
| NP_055665.1 | SLIT-ROBO Rho GTPase-activating protein 3 isoform a | | |  |  |
| AECGTTRPPCLPPKPQKMR | 2 | 2.49 | 3 |  |  |
| FSSKIRSSR | 2 | 2.45 | 4 |  |  |
| NP_003350.1 | UDP-glucose 6-dehydrogenase isoform 1 | |  |  |  |
| NP_001171629.1 | UDP-glucose 6-dehydrogenase isoform 2 | |  |  |  |
| VLIGGDETPEGQR | 2 | 4.23 | 6 |  |  |
| VTVVDVNESR | 2 | 4.13 | 6 |  |  |
| NP_004229.1 | probable E3 ubiquitin-protein ligase TRIP12 | |  |  |  |
| DAVSR | 1 | 1.53 | 11 |  |  |
| EGQQSFHLS | 1 | 1.88 | 11 |  |  |
| NP_065996.1 | WD repeat- and FYVE domain-containing protein 4 | | |  |  |
| LIAKEMNISSR | 1 | 1.77 | 7 |  |  |
| QVMCEAGLLGTLMASCHR | 2 | 3.70 | 7 |  |  |
| NP_060250.2 | chromodomain-helicase-DNA-binding protein 7 | | |  |  |
| NLMEMVAQLR | 2 | 2.87 | 9 |  |  |
| IPVINLEDGTR | 2 | 2.26 | 9 |  |  |
| NP_002625.1 | prohibitin |  |  |  |  |
| IFTSIGEDYDER | 2 | 4.51 | 10 |  |  |
| FDAGELITQRELVSR | 2 | 2.58 | 11 |  |  |
| NVPVITGSKDLQNVNITLR | 3 | 2.99 | 10 |  |  |
| FDAGELITQR | 2 | 4.24 | 10 |  |  |
| NP_056355.2 | RNA polymerase II-associated protein 1 | |  |  |  |
| LYHRASDTPSGLSPTDTMGTAMR | 2 | 2.33 | 7 |  |  |
| HDQHITAVLTKIIER | 2 | 2.23 | 7 |  |  |
| NP_001360.1 | dynein heavy chain 5, axonemal |  |  |  |  |
| LTSTQGSLVEDESLIVVLSNTKR | 2 | 2.55 | 12 |  |  |
| VILTEKQELEKER | 2 | 2.52 | 11 |  |  |
| NP_071342.2 | C-X-C motif chemokine 16 precursor | |  |  |  |
| NP_001094282.1 | C-X-C motif chemokine 16 precursor | |  |  |  |
| MSGSQSEVAPSPQSPR | 2 | 2.10 | 7 |  |  |
| SPEMGRDLRPGSR | 2 | 2.27 | 6 |  |  |
| NP_060482.2 | kinesin-like protein KIF26B |  |  |  |  |
| SPASPR | 1 | 1.54 | 9 |  |  |
| AVSGRISELLQGGAGAR | 2 | 2.82 | 9 |  |  |
| NP_705618.1 | nuclear pore complex protein Nup155 isoform 1 | | |  |  |
| NP_004289.1 | nuclear pore complex protein Nup155 isoform 2 | | |  |  |
| YYEKNRSFSNAAR | 2 | 2.12 | 5 |  |  |
| QPLARPNTLTLVHVR | 2 | 2.08 | 6 |  |  |
| NP_064533.3 | protein spire homolog 1 isoform b | |  |  |  |
| NP_001122098.1 | protein spire homolog 1 isoform a | |  |  |  |
| QFLPPSRQSSR | 2 | 2.85 | 12 |  |  |
| EPGAAGGAAGGSRDALSLEEILR | 2 | 2.26 | 12 |  |  |
| NP_065825.1 | E3 ubiquitin-protein ligase MIB1 |  |  |  |  |
| WTFNPAVLTKANIVR | 2 | 2.48 | 2 |  |  |
| CSGAYDLR | 2 | 2.20 | 2 |  |  |
| NP_689506.2 | transcriptional adapter 2-beta |  |  |  |  |
| EEQLLLDAIEQFGFGNWE DMAAHVGASR | 3 | 3.23 | 1 |  |  |
| LSPAR | 1 | 1.82 | 1 |  |  |
| NP_523240.1 | cyclin-dependent kinase 4 inhibitor C | |  |  |  |
| NP_001253.1 | cyclin-dependent kinase 4 inhibitor C | |  |  |  |
| NEVVSLMQANGAGGATNLQ | 2 | 2.57 | 11 |  |  |
| TALQVMKLGNPEIAR | 2 | 2.41 | 11 |  |  |
| NP_001073879.2 | F-box only protein 41 |  |  |  |  |
| LERLSEEVEQKIAGQVGR | 2 | 2.37 | 10 |  |  |
| ALEKLEVDR | 2 | 2.10 | 11 |  |  |
| NP_003364.1 | vinculin isoform VCL |  |  |  |  |
| NP_054706.1 | vinculin isoform meta-VCL |  |  |  |  |
| EEVFDER | 2 | 2.25 | 3 |  |  |
| DPSASPGDAGEQAIR | 2 | 4.42 | 3 |  |  |
| ELTPQVVSAAR | 2 | 3.54 | 3 |  |  |
| MALLMAEMSR | 2 | 2.39 | 4 |  |  |
| GLVAEGHR | 2 | 2.45 | 3 |  |  |
| TDAGFTLR | 2 | 2.60 | 3 |  |  |
| NP_066363.1 | NFX1-type zinc finger-containing protein 1 | |  |  |  |
| LLREDFVRPLR | 2 | 2.04 | 6 |  |  |
| YLLMGGR | 1 | 1.69 | 7 |  |  |
| NP_001008800.1 | T-complex protein 1 subunit gamma isoform c | | |  |  |
| NP_005989.3 | T-complex protein 1 subunit gamma isoform a | | |  |  |
| IARACGAR | 1 | 1.57 | 12 |  |  |
| ACGARIVSRPEELR | 2 | 2.12 | 12 |  |  |
| NP_065744.2 | Down syndrome cell adhesion molecule-like protein 1 | | |  |  |
| YEELAR | 1 | 2.38 | 7 |  |  |
| NTELVLPDEAISIR | 2 | 3.52 | 8 |  |  |
| NP_001122390.1 | MAP/microtubule affinity-regulating kinase 3 isoform a | | |  |  |
| NP_001122391.1 | MAP/microtubule affinity-regulating kinase 3 isoform b | | |  |  |
| NP_001122392.1 | MAP/microtubule affinity-regulating kinase 3 isoform d | | |  |  |
| NP_001122393.1 | MAP/microtubule affinity-regulating kinase 3 isoform e | | |  |  |
| NP_002367.4 | MAP/microtubule affinity-regulating kinase 3 isoform c | | |  |  |
| FLVLNPIKR | 2 | 2.33 | 2 |  |  |
| FPRGTASR | 1 | 1.63 | 1 |  |  |
| NP_055756.1 | MORC family CW-type zinc finger protein 2 | |  |  |  |
| TPESTQIGQYGNGLKSGSMR | 2 | 2.29 | 11 |  |  |
| QVQNRAITLR | 2 | 2.19 | 10 |  |  |
| NP_863654.1 | cytosolic acyl coenzyme A thioester hydrolase isoform hBACHb | | | |  |
| FEEGKGR | 1 | 1.81 | 6 |  |  |
| LVAGQGCVGPRR | 2 | 2.12 | 5 |  |  |
| NP_002273.3 | keratin, type II cuticular Hb3 |  |  |  |  |
| NP_002272.2 | keratin, type II cuticular Hb1 |  |  |  |  |
| NP_002275.1 | keratin, type II cuticular Hb6 |  |  |  |  |
| RLLEGEEQR | 2 | 2.89 | 10 |  |  |
| LLEGEEQR | 2 | 3.49 | 11 |  |  |
| LCEGIGAVNVCVSSSR | 2 | 3.19 | 11 |  |  |
| TKEEINELNR | 2 | 3.97 | 11 |  |  |
| NP_695006.1 | bestrophin-4 |  |  |  |  |
| FGGFSGLLLR | 2 | 2.05 | 11 |  |  |
| YANLASVLVLR | 2 | 2.19 | 11 |  |  |
| NP_003008.1 | serine/arginine-rich splicing factor 3 | |  |  |  |
| AFGYYGPLR | 2 | 3.29 | 11 |  |  |
| NPPGFAFVEFEDPR | 2 | 4.60 | 11 |  |  |
| NP_001230199.1 | contactin-5 isoform 1 precursor |  |  |  |  |
| NP_001230200.1 | contactin-5 isoform 3 precursor |  |  |  |  |
| NP_780775.1 | contactin-5 isoform 2 precursor |  |  |  |  |
| NP_055176.1 | contactin-5 isoform 1 precursor |  |  |  |  |
| NGTEIDLESDYR | 2 | 2.85 | 8 |  |  |
| MIRTNEAVPKTAPTNVSGR | 3 | 2.88 | 7 |  |  |
| NP_065073.3 | brefeldin A-inhibited guanine nucleotide-exchange protein 3 | | | |  |
| EWLGRVGR | 1 | 1.52 | 6 |  |  |
| SLSTAPVVQPLSIQDLVR | 2 | 2.00 | 5 |  |  |
| NP_001034200.1 | tripartite motif-containing protein 71 | |  |  |  |
| ALDILLAR | 2 | 2.21 | 6 |  |  |
| SAPGGPAASPSALLLR | 2 | 2.22 | 7 |  |  |
| NP_057417.3 | serine/arginine repetitive matrix protein 2 | |  |  |  |
| SPSPASGRR | 2 | 2.15 | 11 |  |  |
| TPTAPAVNLAGAR | 2 | 2.39 | 10 |  |  |
| NP_115997.5 | unconventional myosin-XVIIIb |  |  |  |  |
| FELEIER | 1 | 1.65 | 10 |  |  |
| FKGDVACQVLESER | 2 | 3.02 | 9 |  |  |
| NP_004184.1 | Golgi-specific brefeldin A-resistance guanine nucleotide exchange factor 1  isoform 1 | | | | |
| NP_001186307.1 | Golgi-specific brefeldin A-resistance guanine nucleotide exchange factor 1  isoform 2 | | | | |
| NP_001186308.1 | Golgi-specific brefeldin A-resistance guanine nucleotide exchange factor 1  isoform 3 | | | | |
| GESTVLSFVSWLTLSGPEQSSVR | 3 | 3.35 | 3 |  |  |
| RLSELLR | 2 | 2.62 | 4 |  |  |
| NP_008823.1 | DNA (cytosine-5)-methyltransferase 3B isoform 1 | | |  |  |
| NP_787044.1 | DNA (cytosine-5)-methyltransferase 3B isoform 2 | | |  |  |
| NP_787045.1 | DNA (cytosine-5)-methyltransferase 3B isoform 3 | | |  |  |
| NP_787046.1 | DNA (cytosine-5)-methyltransferase 3B isoform 6 | | |  |  |
| NHVDESPVEFPATR | 2 | 2.27 | 2 |  |  |
| KGLYEGTGR | 2 | 2.21 | 2 |  |  |
| NP_057322.2 | anaphase-promoting complex subunit 7 isoform a | | |  |  |
| NP_001131136.1 | anaphase-promoting complex subunit 7 isoform b | | |  |  |
| ILYRLFESVLPPLPAALQSR | 2 | 2.56 | 3 |  |  |
| EQKYEDGIALLR | 2 | 2.67 | 2 |  |  |
| NP_976048.1 | zinc finger CCHC domain-containing protein 13 | | |  |  |
| QIPTSSQGMSQ | 1 | 2.58 | 5 |  |  |
| GGAGGRR | 1 | 1.58 | 6 |  |  |
| NP_001073930.1 | homeobox protein unc-4 homolog | |  |  |  |
| LDLVESR | 1 | 1.89 | 8 |  |  |
| EALALR | 1 | 1.55 | 8 |  |  |
| NP_001177396.1 | putative uncharacterized protein FLJ22184 | |  |  |  |
| VLGAAGAVGR | 1 | 1.95 | 8 |  |  |
| SALSAGARR | 1 | 1.66 | 7 |  |  |
| NP_002145.3 | heat shock 70 kDa protein 4 |  |  |  |  |
| AGGIETIANEYSDR | 2 | 4.62 | 4 |  |  |
| EFSITDVVPYPISLR | 2 | 4.35 | 4 |  |  |
| NP_056134.2 | tensin-like C1 domain-containing phosphatase isoform 1 | | |  |  |
| NP_736610.2 | tensin-like C1 domain-containing phosphatase isoform 2 | | |  |  |
| NP_938072.1 | tensin-like C1 domain-containing phosphatase isoform 3 | | |  |  |
| RYISYFSGLLSGSIR | 2 | 2.51 | 9 |  |  |
| SPVPTTLPGLR | 2 | 2.16 | 10 |  |  |
| NP_002215.2 | inositol 1,4,5-trisphosphate receptor type 3 | |  |  |  |
| EPVDPTTKGRVASFSIPGSSSR | 3 | 2.60 | 10 |  |  |
| QRLGFVDVQNCISR | 2 | 2.65 | 9 |  |  |
| NP_005773.3 | THO complex subunit 4 |  |  |  |  |
| SLGTADVHFER | 2 | 2.25 | 9 |  |  |
| GAGGFGGGGGTR | 1 | 1.65 | 8 |  |  |
| NP_443196.2 | cytokine-dependent hematopoietic cell linker | | |  |  |
| MPSQISLR | 2 | 2.57 | 10 |  |  |
| MNKPLLDWER | 2 | 2.24 | 9 |  |  |
| LERVDKPISKDVR | 2 | 2.23 | 9 |  |  |
| NP_065178.1 | actin-related protein 3B isoform 1 | |  |  |  |
| NP_005712.1 | actin-related protein 3 |  |  |  |  |
| LSEELSGGR | 2 | 3.25 | 7 |  |  |
| DITYFIQQLLR | 2 | 3.22 | 7 |  |  |
| NP_066919.2 | voltage-dependent T-type calcium channel subunit alpha-1I isoform a | | | | |
| NP_001003406.1 | voltage-dependent T-type calcium channel subunit alpha-1I isoform b | | | | |
| LSPAAR | 1 | 1.56 | 9 |  |  |
| RTLSLDNR | 2 | 2.06 | 9 |  |  |
| SPSWAADR | 1 | 1.81 | 10 |  |  |
| NP_064695.3 | aldo-keto reductase family 1 member B10 | |  |  |  |
| ACNVLQSSHLEDYPFNAEY | 2 | 4.02 | 8 |  |  |
| FHIQR | 1 | 1.54 | 8 |  |  |
| NP_071925.2 | ectoderm-neural cortex protein 2 | |  |  |  |
| YFEAMFSHGLRESR | 2 | 2.07 | 4 |  |  |
| KEFSASAIGCKVYVTGGR | 2 | 2.29 | 5 |  |  |
| NP_005843.2 | chromodomain-helicase-DNA-binding protein 3 isoform 2 | | |  |  |
| NP_001005273.1 | chromodomain-helicase-DNA-binding protein 3 isoform 1 | | |  |  |
| NP_001005271.2 | chromodomain-helicase-DNA-binding protein 3 isoform 3 | | |  |  |
| DEYREKSESGGSEYGTGPGR | 2 | 2.44 | 8 |  |  |
| IDGGITGALR | 1 | 1.51 | 9 |  |  |
| NP_079054.3 | LON peptidase N-terminal domain and RING finger protein 3 isoform 2 | | | | |
| NP_001027026.1 | LON peptidase N-terminal domain and RING finger protein 3 isoform 1 | | | | |
| LNGIR | 1 | 1.93 | 9 |  |  |
| AQLPFLAMR | 1 | 2.05 | 9 |  |  |
| NP_001188474.1 | putative RNA-binding protein 15 isoform 2 | |  |  |  |
| NP_073605.4 | putative RNA-binding protein 15 isoform 1 | |  |  |  |
| AKLAMSGKIIIR | 2 | 2.14 | 8 |  |  |
| EYDTGGGSSSSR | 1 | 1.76 | 8 |  |  |
| KNSASAER | 2 | 2.09 | 8 |  |  |
| NP_003310.4 | titin isoform N2-B |  |  |  |  |
| NP_596869.4 | titin isoform N2-A |  |  |  |  |
| NP_597676.3 | titin isoform novex-1 |  |  |  |  |
| NP_597681.3 | titin isoform novex-2 |  |  |  |  |
| ATAVIKFCDR | 2 | 2.02 | 11 |  |  |
| VVAEEKVPVPR | 2 | 2.22 | 10 |  |  |
| FEVLHGREAKVTETAR | 2 | 3.49 | 11 |  |  |
| DSMEVQWNEPISDGGSR | 2 | 3.11 | 11 |  |  |
| NP_001243731.1 | 40S ribosomal protein S3 |  |  |  |  |
| NP_000996.2 | 40S ribosomal protein S3 |  |  |  |  |
| TEIIILATR | 2 | 4.06 | 9 |  |  |
| ELAEDGYSGVEVR | 2 | 4.92 | 9 |  |  |
| NP_733821.1 | prelamin-A/C isoform prelamin A precursor | |  |  |  |
| NP_733822.1 | prelamin-A/C isoform prelamin A delta10 | |  |  |  |
| NP_005563.1 | prelamin-A/C isoform lamin C |  |  |  |  |
| LAVYIDR | 2 | 2.54 | 5 |  |  |
| ITESEEVVSR | 2 | 4.13 | 5 |  |  |
| SGAQASSTPLSPTR | 2 | 4.71 | 5 |  |  |
| LADALQELR | 2 | 3.94 | 5 |  |  |
| TLEGELHDLR | 2 | 3.53 | 5 |  |  |
| SLETENAGLR | 2 | 3.64 | 5 |  |  |
| NP_001167541.1 | protein FAM53A |  |  |  |  |
| NP_001013644.1 | protein FAM53A |  |  |  |  |
| ESVTSDGSRR | 2 | 2.88 | 12 |  |  |
| RCDSGGSATR | 1 | 1.81 | 12 |  |  |
| NP_004749.2 | peripheral-type benzodiazepine receptor-associated protein 1 isoform a | | | | |
| NP_077729.1 | peripheral-type benzodiazepine receptor-associated protein 1 isoform b | | | | |
| CEELELQLR | 2 | 2.58 | 10 |  |  |
| GGGSPEKPPSR | 2 | 2.18 | 10 |  |  |
| NP_150229.2 | multidrug resistance-associated protein 9 | |  |  |  |
| FFDTTPTGR | 2 | 2.14 | 4 |  |  |
| GVQELKKVENVSR | 2 | 2.85 | 4 |  |  |
| NP_001193727.1 | pyruvate kinase isozymes M1/M2 isoform e | |  |  |  |
| EAEAAIYHLQLFEELR | 2 | 5.86 | 6 |  |  |
| APIIAVTR | 1 | 2.33 | 6 |  |  |
| NP_115645.4 | coiled-coil domain-containing protein 135 | |  |  |  |
| EEKLSR | 1 | 1.66 | 10 |  |  |
| FEQEQEVKKQQEIR | 2 | 2.61 | 9 |  |  |
| NP_001108104.1 | jmjC domain-containing protein 7 | |  |  |  |
| FMMPAER | 1 | 1.68 | 8 |  |  |
| YPSYSQAQALR | 2 | 2.02 | 8 |  |  |
| NP_009099.1 | ATP-binding cassette sub-family A member 8 | | |  |  |
| KGCFSKRKNKIATR | 2 | 2.61 | 7 |  |  |
| KNKIATR | 1 | 1.52 | 6 |  |  |
| NP_065799.1 | integrator complex subunit 2 |  |  |  |  |
| AFPPLYEDIMSLLIQIGQ VCASDVATQTR | 3 | 2.54 | 10 |  |  |
| QDEESLGGR | 1 | 1.68 | 11 |  |  |
| NP_997270.2 | leucine-rich repeat-containing protein FAM211A isoform 2 | | | |  |
| NP_001107039.1 | leucine-rich repeat-containing protein FAM211A isoform 1 | | | |  |
| QTKGSLAER | 1 | 1.60 | 11 |  |  |
| RERPDFWASLLLR | 2 | 2.75 | 12 |  |  |
| NP_653292.2 | calpain-12 |  |  |  |  |
| CCLRPGHYLVVPSTAHAG DEADFTLR | 3 | 2.62 | 1 |  |  |
| TDVCQGSLGNCWFLAA AASLTLYPRLLR | 3 | 2.55 | 1 |  |  |
| NP_004822.2 | mediator of RNA polymerase II transcription subunit 26 | | |  |  |
| NDLQRLPGQR | 2 | 2.23 | 4 |  |  |
| ADSPVHMEQQSR | 2 | 2.43 | 3 |  |  |
| NP_872314.1 | uncharacterized protein C13orf30 | |  |  |  |
| EALSYALVLRDSTKR | 3 | 2.52 | 5 |  |  |
| EALSYALVLR | 2 | 2.24 | 5 |  |  |
| NP_064710.4 | UPF0505 protein C16orf62 |  |  |  |  |
| MAPAGVVTR | 2 | 2.06 | 10 |  |  |
| SLGLNLALADPPESDR | 3 | 2.62 | 11 |  |  |
| NP_008855.1 | serine/arginine-rich splicing factor 1 isoform 1 | | |  |  |
| NP_001071634.1 | serine/arginine-rich splicing factor 1 isoform 2 | | |  |  |
| DGTGVVEFVR | 2 | 3.14 | 9 |  |  |
| GGGGAPR | 1 | 1.51 | 10 |  |  |
| SHEGETAYIR | 2 | 3.69 | 9 |  |  |
| IYVGNLPPDIR | 2 | 3.88 | 9 |  |  |
| DAEDAVYGR | 2 | 3.14 | 9 |  |  |
| EAGDVCYADVYR | 2 | 3.56 | 9 |  |  |
| NP_004658.3 | E3 ubiquitin-protein ligase HERC2 | |  |  |  |
| FLGVLLGIAIR | 2 | 2.03 | 12 |  |  |
| LSSLPR | 1 | 1.68 | 11 |  |  |
| SSGIDCKIHGLILLGR | 2 | 2.11 | 12 |  |  |
| NP_000194.2 | alpha-L-iduronidase precursor |  |  |  |  |
| THWLLELVTTRGSTGR | 2 | 2.00 | 5 |  |  |
| LGGPGDSFHTPPR | 2 | 2.06 | 6 |  |  |
| NP_001075109.1 | DNA-dependent protein kinase catalytic subunit isoform 2 | | | |  |
| NP_008835.5 | DNA-dependent protein kinase catalytic subunit isoform 1 | | | |  |
| WVELAKLYR | 1 | 1.62 | 7 |  |  |
| DQNILLGTTYR | 2 | 2.13 | 7 |  |  |
| NP_057095.2 | trans-2-enoyl-CoA reductase, mitochondrial isoform a | | |  |  |
| MWVCSTLWRVR | 2 | 2.28 | 11 |  |  |
| LALNCVGGKSSTELLR | 2 | 2.47 | 12 |  |  |
| NP_001087.2 | ATP-citrate synthase isoform 1 |  |  |  |  |
| NP_942127.1 | ATP-citrate synthase isoform 2 |  |  |  |  |
| GGPNYQEGLR | 2 | 3.26 | 3 |  |  |
| TIAIIAEGIPEALTR | 2 | 5.48 | 3 |  |  |
| NP_000680.2 | retinal dehydrogenase 1 |  |  |  |  |
| IFVEESIYDEFVR | 2 | 5.38 | 6 |  |  |
| LADLIER | 2 | 2.42 | 6 |  |  |
| TIPIDGNFFTYTR | 2 | 3.43 | 6 |  |  |
| NP_001338.2 | diacylglycerol kinase theta |  |  |  |  |
| DARADAAPAPESDPR | 2 | 2.00 | 2 |  |  |
| EGNLPSGAR | 1 | 1.69 | 2 |  |  |
| FYVAESR | 1 | 1.58 | 3 |  |  |
| NP_056142.2 | protein SMG5 |  |  |  |  |
| YLEAEFKKGNR | 2 | 2.04 | 2 |  |  |
| TNPDLIIVCAQSSQSLWNR | 2 | 2.10 | 3 |  |  |
| NP_056390.2 | PERQ amino acid-rich with GYF domain-containing protein 2 isoform b | | | | |
| NP_001096616.1 | PERQ amino acid-rich with GYF domain-containing protein 2 isoform b | | | | |
| NP_001096617.1 | PERQ amino acid-rich with GYF domain-containing protein 2 isoform a | | | | |
| EEQNGEDEDGGWR | 2 | 2.42 | 3 |  |  |
| WRPHSPDGPR | 2 | 2.10 | 2 |  |  |
| NP_112552.1 | heterogeneous nuclear ribonucleoprotein K isoform b | | |  |  |
| NP_112553.1 | heterogeneous nuclear ribonucleoprotein K isoform a | | |  |  |
| NP_002131.2 | heterogeneous nuclear ribonucleoprotein K isoform a | | |  |  |
| TDYNASVSVPDSSGPER | 2 | 4.82 | 6 |  |  |
| VVLIGGKPDR | 2 | 2.90 | 6 |  |  |
| NLPLPPPPPPR | 2 | 2.50 | 6 |  |  |
| GRPVGFPMRGR | 2 | 2.23 | 6 |  |  |
| NP_005631.1 | transcription initiation factor TFIID subunit 4B | | |  |  |
| EMKYSRALYLALLK | 2 | 2.44 | 7 |  |  |
| KDLEEREMLLKAAKSR | 2 | 2.34 | 6 |  |  |
| NP_699199.2 | gap junction delta-4 protein |  |  |  |  |
| RRPGPPTSPSIR | 2 | 2.34 | 10 |  |  |
| IVLAGR | 1 | 1.65 | 9 |  |  |
| NP_542189.1 | abhydrolase domain-containing protein 16B | |  |  |  |
| GWPVAFRWDDVR | 2 | 2.07 | 1 |  |  |
| TQDDVVSTSGR | 1 | 1.52 | 1 |  |  |
| NP_001074003.1 | protein unc-13 homolog C |  |  |  |  |
| TLHGLKLGALR | 2 | 2.51 | 2 |  |  |
| EDARGLTPR | 2 | 2.86 | 1 |  |  |
| NP_543151.2 | E3 ubiquitin-protein ligase MIB2 isoform 1 | |  |  |  |
| NP_001164157.1 | E3 ubiquitin-protein ligase MIB2 isoform 2 | |  |  |  |
| NP_001164158.1 | E3 ubiquitin-protein ligase MIB2 isoform 3 | |  |  |  |
| NP_001164159.1 | E3 ubiquitin-protein ligase MIB2 isoform 4 | |  |  |  |
| APALGR | 1 | 1.75 | 12 |  |  |
| RATGR | 1 | 1.54 | 12 |  |  |
| SLARCGPSSR | 2 | 2.30 | 11 |  |  |
| NP_055907.3 | serine/threonine-protein kinase SMG1 | |  |  |  |
| NLVLKESQR | 2 | 2.13 | 11 |  |  |
| NSASPKHSLNGESR | 2 | 2.01 | 10 |  |  |
| NP_689854.2 | AT-rich interactive domain-containing protein 2 | | |  |  |
| KLGINDIEGQR | 2 | 2.17 | 4 |  |  |
| DFVKFWKDIVDDNEVR | 2 | 2.85 | 5 |  |  |
| NP_004896.1 | peroxiredoxin-6 |  |  |  |  |
| NFDEILR | 2 | 2.69 | 11 |  |  |
| DFTPVCTTELGR | 2 | 3.66 | 11 |  |  |
| NP_598377.3 | A disintegrin and metalloproteinase with thrombospondin motifs 19 preproprotein | | | | |
| GVGGGGSAR | 1 | 1.86 | 8 |  |  |
| TCSAGISSRER | 1 | 2.05 | 8 |  |  |
| NP_056969.2 | pre-mRNA cleavage complex 2 protein Pcf11 | |  |  |  |
| FEGCHALR | 1 | 1.77 | 3 |  |  |
| SPIIHSPKR | 2 | 2.25 | 4 |  |  |
| NP_004238.3 | 116 kDa U5 small nuclear ribonucleoprotein component isoform a | | | |  |
| NP_001136077.1 | 116 kDa U5 small nuclear ribonucleoprotein component isoform b | | | |  |
| GGGQIIPTAR | 2 | 3.06 | 4 |  |  |
| EGPLCDELIR | 2 | 2.05 | 3 |  |  |
| NP_653297.3 | axonemal dynein light chain domain-containing protein 1 | | | |  |
| MENDMKKLVAER | 2 | 2.56 | 9 |  |  |
| VEVAQLNDVMDTMLER | 2 | 2.73 | 9 |  |  |
| NP_056419.2 | DDB1- and CUL4-associated factor 4 isoform 1 | | |  |  |
| NP_851938.2 | DDB1- and CUL4-associated factor 4 isoform 3 | | |  |  |
| NP_001156980.1 | DDB1- and CUL4-associated factor 4 isoform 4 | | |  |  |
| MNKSRWQSR | 1 | 1.60 | 8 |  |  |
| DSEDRSDSR | 2 | 2.22 | 8 |  |  |
| NP_002256.2 | importin subunit beta-1 |  |  |  |  |
| VLANPGNSQVAR | 2 | 3.84 | 4 |  |  |
| DTAAWTVGR | 2 | 2.55 | 4 |  |  |
| NP_006422.1 | T-complex protein 1 subunit beta isoform 1 | |  |  |  |
| NP_001185771.1 | T-complex protein 1 subunit beta isoform 2 | |  |  |  |
| VQDDEVGDGTTSVTVLAAELLR | 2 | 6.99 | 6 |  |  |
| QVLLSAAEAAEVILR | 2 | 3.71 | 6 |  |  |
| GATQQILDEAER | 2 | 4.67 | 6 |  |  |
| NP_055456.2 | mediator of DNA damage checkpoint protein 1 | | |  |  |
| FTPELQPKASQSRKR | 2 | 2.27 | 3 |  |  |
| EAVLKDHTKIRALVR | 2 | 2.14 | 2 |  |  |
| NP_061142.2 | ATP-binding cassette sub-family A member 5 | | |  |  |
| NP_758424.1 | ATP-binding cassette sub-family A member 5 | | |  |  |
| KKGENVEALR | 2 | 2.19 | 2 |  |  |
| GIGYR | 1 | 1.55 | 1 |  |  |
| NP_064550.3 | E3 ubiquitin-protein ligase RAD18 | |  |  |  |
| EIENIEKTRMR | 2 | 2.73 | 2 |  |  |
| MDSLAESR | 1 | 1.54 | 2 |  |  |
| NP_000758.1 | cytochrome P450 2B6 precursor |  |  |  |  |
| FSVTTMR | 1 | 1.61 | 12 |  |  |
| FSDLLPMGVPHIVTQHTSFR | 2 | 2.89 | 12 |  |  |
| NP_079466.3 | ankyrin repeat domain-containing protein 36B | | |  |  |
| SALILAVTLGEKDIVILL LQHNIDVFSR | 3 | 2.93 | 5 |  |  |
| EKKDGEISR | 2 | 2.30 | 4 |  |  |
| NP_004318.3 | breakpoint cluster region protein isoform 1 | |  |  |  |
| NP_067585.2 | breakpoint cluster region protein isoform 2 | |  |  |  |
| IVGVR | 1 | 1.67 | 7 |  |  |
| QCVEEIER | 1 | 1.98 | 6 |  |  |
| NP_847884.2 | RILP-like protein 1 |  |  |  |  |
| TSPQPESGIKR | 2 | 2.99 | 3 |  |  |
| VLEILEVLVSR | 2 | 2.24 | 4 |  |  |
| NP_001242954.1 | rho GTPase-activating protein 22 isoform 2 | |  |  |  |
| IEEGSADLR | 1 | 1.98 | 4 |  |  |
| MLPTASSKR | 1 | 2.11 | 5 |  |  |
| NP_002335.2 | leukocyte tyrosine kinase receptor isoform 1 precursor | | |  |  |
| CVGLSLRATPR | 2 | 2.26 | 12 |  |  |
| GGAAGGGGGWTSR | 2 | 2.27 | 11 |  |  |
| NP_112210.1 | phosphatase and actin regulator 1 | |  |  |  |
| NP_001229577.1 | phosphatase and actin regulator 1 | |  |  |  |
| SKSDTPYLAEAR | 2 | 2.46 | 7 |  |  |
| RADKPWTR | 2 | 2.17 | 7 |  |  |
| NP_149120.1 | transcriptional repressor scratch 2 | |  |  |  |
| RGGGGGDAGGSGDAGGAGGR | 2 | 2.09 | 10 |  |  |
| AGAQAGGGHR | 1 | 1.53 | 11 |  |  |
| NP_055940.3 | protein capicua homolog |  |  |  |  |
| HPNQDNR | 2 | 2.83 | 7 |  |  |
| TSAKGPETMASKFPSSSSDWR | 2 | 2.15 | 8 |  |  |
| NP_002784.1 | proteasome subunit beta type-1 |  |  |  |  |
| LSEGFSIHTR | 2 | 3.70 | 11 |  |  |
| DVYTGDALR | 2 | 2.49 | 11 |  |  |
| NP_006089.1 | guanine nucleotide-binding protein subunit beta-2-like 1 | | |  |  |
| VWQVTIGTR | 2 | 3.88 | 9 |  |  |
| DETNYGIPQR | 2 | 3.77 | 10 |  |  |
| LWDLTTGTTTR | 2 | 3.82 | 9 |  |  |
| NP_001018126.1 | muscle-related coiled-coil protein | |  |  |  |
| FKKSISNAAPSKEAFKMR | 2 | 2.25 | 1 |  |  |
| SLRKGKDR | 1 | 1.63 | 1 |  |  |
| NP_000691.1 | annexin A1 |  |  |  |  |
| ALYEAGER | 2 | 2.91 | 8 |  |  |
| SEDFGVNEDLADSDAR | 2 | 5.46 | 8 |  |  |
| KGTDVNVFNTILTTR | 2 | 6.22 | 8 |  |  |
| NP_783318.1 | zygote arrest protein 1 |  |  |  |  |
| IDAAVQCSLGRR | 2 | 2.10 | 11 |  |  |
| TVAVYSPLALRR | 2 | 2.26 | 12 |  |  |
| NP_001145.1 | annexin A5 |  |  |  |  |
| GTVTDFPGFDER | 2 | 4.19 | 9 |  |  |
| SEIDLFNIR | 2 | 3.59 | 9 |  |  |
| NP_061836.2 | bromodomain and WD repeat-containing protein 1 isoform A | | | |  |
| NP_387505.1 | bromodomain and WD repeat-containing protein 1 isoform B | | | |  |
| VTSLLGAGR | 2 | 2.54 | 3 |  |  |
| QPVDLVEYPDYRDIIDTPMDFGTVR | 3 | 2.57 | 2 |  |  |
| NP_009057.1 | transitional endoplasmic reticulum ATPase | |  |  |  |
| ESIESEIR | 2 | 2.63 | 4 |  |  |
| EVDIGIPDATGR | 2 | 3.68 | 4 |  |  |
| RSVSDNDIR | 2 | 2.72 | 4 |  |  |
| GGNIGDGGGAADR | 2 | 4.34 | 4 |  |  |
| KGDIFLVR | 2 | 3.00 | 4 |  |  |
| SVSDNDIR | 2 | 2.66 | 4 |  |  |
| NP_004964.2 | protein Jumonji |  |  |  |  |
| FEPKNGLIHGVAPR | 2 | 2.19 | 3 |  |  |
| ATVDVPPSR | 2 | 2.02 | 2 |  |  |
| NP_055906.2 | protein FAM179B |  |  |  |  |
| VSRNLFQNSR | 1 | 1.53 | 12 |  |  |
| EENPALR | 1 | 1.93 | 12 |  |  |
| NP_055796.1 | myosin-15 precursor |  |  |  |  |
| CNGVLEGTR | 1 | 1.70 | 10 |  |  |
| LEEKEALINQLSR | 2 | 2.70 | 9 |  |  |
| NP_612355.1 | uncharacterized protein KIAA2013 precursor | |  |  |  |
| MWLQQRLKGLPGLLSSSWAR | 3 | 2.59 | 1 |  |  |
| GEVVPLGPGVPALVANGF LALDVAANR | 3 | 2.55 | 1 |  |  |
| NP_001231639.1 | glucosamine--fructose-6-phosphate aminotransferase [isomerizing] 1 isoform 1 | | | | |
| NP_002047.2 | glucosamine--fructose-6-phosphate aminotransferase [isomerizing] 1 isoform 2 | | | | |
| YCKERGALTVGITNTVGSSISR | 2 | 2.61 | 6 |  |  |
| GALTVGITNTVGSSISR | 2 | 2.04 | 7 |  |  |
| NP_002038.2 | glycine--tRNA ligase precursor |  |  |  |  |
| LLEFNQGKLPFAAAQIGNSFR | 2 | 2.54 | 5 |  |  |
| LGDAVEQGVINNTVLGYFIGR | 2 | 5.98 | 5 |  |  |
| NP_597732.1 | ankyrin repeat domain-containing protein 24 | | |  |  |
| QLSPSAQEHLASLQEQVAVLTR | 3 | 2.56 | 4 |  |  |
| EALFMKSER | 1 | 1.94 | 5 |  |  |
| NP_150254.1 | 60S ribosomal protein L13 isoform 1 | |  |  |  |
| NP_000968.2 | 60S ribosomal protein L13 isoform 1 | |  |  |  |
| NP_001230059.1 | 60S ribosomal protein L13 isoform 2 | |  |  |  |
| NP_001230060.1 | 60S ribosomal protein L13 isoform 3 | |  |  |  |
| VATWFNQPAR | 2 | 3.50 | 11 |  |  |
| TIGISVDPR | 2 | 2.41 | 11 |  |  |
| GFSLEELR | 2 | 2.85 | 11 |  |  |
| NP_003602.1 | oral-facial-digital syndrome 1 protein | |  |  |  |
| VKELQQEAER | 2 | 2.38 | 10 |  |  |
| VVSEQPQVGTLEER | 2 | 2.01 | 10 |  |  |
| NP_689472.3 | treslin |  |  |  |  |
| LLFQQLVSR | 2 | 2.28 | 12 |  |  |
| AVGCGAGSSSGR | 2 | 2.07 | 11 |  |  |
| NP_001367.2 | cytoplasmic dynein 1 heavy chain 1 | |  |  |  |
| IQGLTVEQAEAVVR | 2 | 2.18 | 3 |  |  |
| DLEASIAR | 2 | 2.12 | 4 |  |  |
| NP_006658.1 | membrane-associated progesterone receptor component 1 | | | |  |
| DFTPAELR | 2 | 2.25 | 11 |  |  |
| FDGVQDPR | 2 | 2.25 | 11 |  |  |
| NP_055427.2 | constitutive coactivator of PPAR-gamma-like protein 1 | | |  |  |
| GSLVGGGR | 1 | 1.74 | 7 |  |  |
| NHMDITTPPLPPVAPEVLR | 2 | 2.75 | 8 |  |  |
| NP_037535.2 | striatin-4 isoform 1 |  |  |  |  |
| NP_001034966.1 | striatin-4 isoform 2 |  |  |  |  |
| FLDNR | 1 | 1.55 | 10 |  |  |
| SLELNGAVEPSEGAPR | 2 | 2.28 | 10 |  |  |
| NP_001131022.1 | leucine-rich repeat flightless-interacting protein 1 isoform 1 | | | |  |
| NP_001131023.1 | leucine-rich repeat flightless-interacting protein 1 isoform 2 | | | |  |
| RQYEEKNKEFER | 2 | 2.14 | 5 |  |  |
| LEKMKANR | 1 | 1.60 | 6 |  |  |
| NP_958929.1 | kinesin light chain 4 isoform a |  |  |  |  |
| NP_958930.1 | kinesin light chain 4 isoform a |  |  |  |  |
| NP_958931.1 | kinesin light chain 4 isoform b |  |  |  |  |
| LSQEEILGSTR | 2 | 2.93 | 5 |  |  |
| QGKLEAAETLEECALR | 2 | 2.95 | 4 |  |  |
| NP_005180.1 | chromobox protein homolog 2 isoform 1 | |  |  |  |
| AVAPPTPASKR | 2 | 2.52 | 9 |  |  |
| MTQSQAQAASR | 2 | 3.21 | 9 |  |  |
| NP_065831.1 | patched domain-containing protein 2 | |  |  |  |
| IELIFLAR | 2 | 2.46 | 12 |  |  |
| AKFQSFVVTYVAMLAKQSTSK VQVLYGGTDLFDYEVR | 3 | 2.86 | 11 |  |  |
