# Supplementary material for: Elucidation of Toxicity Pathways in Lung Epithelial Cells Induced by Silicon Dioxide Nanoparticles
Source: PLoS One. 2013 Sep 4;8(9):e72363. doi: 10.1371/journal.pone.0072363 (PMC3762866; doi:10.1371/journal.pone.0072363)
Supplement: Table S2 — Identification of proteins bound to nano-SiO2 particles. The identification of the proteins listed in Table 3 was based on MS data of tryptic peptides that was analysed by SEQUEST. For each protein the corresponding peptides found are indicated along with their charge and cross-correlation score (Xcorr). The final score (Sf) for each protein is also shown. (DOCX) [file pone.0072363.s003.docx]

**Table S2. Identification of proteins bound to nano-SiO_2_ particles.**

| **Protein description** |  | **NCBI code** | **Protein Sf** |
| --- | --- | --- | --- |
|  | **Peptide [charge]** | **Xcorr** |  |
| albumin precursor [Bos taurus] | | NP_851335.1 | 21.5 |
|  | AEFVEVTK [1+] | 2.75 |  |
|  | AEFVEVTKLVTDLTK [3+] | 3.74 |  |
|  | ALKAWSVAR [2+] | 2.85 |  |
|  | ATEEQLK [2+] | 2.65 |  |
|  | AWSVAR [1+] | 1.67 |  |
|  | DAFLGSFLYEYSR [2+] | 4.59 |  |
|  | DAIPENLPPLTADFAEDK [2+] | 4.20 |  |
|  | DDSPDLPK [2+] | 2.44 |  |
|  | DLGEEHFK [2+] | 2.50 |  |
|  | EACFAVEGPK [2+] | 2.71 |  |
|  | EKVLTSSAR [2+] | 2.23 |  |
|  | FKDLGEEHFK [3+] | 4.59 |  |
|  | FPKAEFVEVTK [3+] | 2.56 |  |
|  | HLVDEPQNLIK [2+] | 3.55 |  |
|  | HPEYAVSVLLR [3+] | 4.60 |  |
|  | HPYFYAPELLYYANK [3+] | 4.52 |  |
|  | KQTALVELLK [2+] | 3.33 |  |
|  | KVPQVSTPTLVEVSR [3+] | 4.88 |  |
|  | LGEYGFQNALIVR [2+] | 4.86 |  |
|  | LKHLVDEPQNLIK [3+] | 4.33 |  |
|  | LSQKFPK [2+] | 2.69 |  |
|  | LVNELTEFAK [2+] | 4.03 |  |
|  | LVTDLTK [1+] | 2.33 |  |
|  | LVVSTQTALA [2+] | 3.33 |  |
|  | QTALVELLK [2+] | 2.44 |  |
|  | RHPEYAVSVLLR [3+] | 4.56 |  |
|  | RHPYFYAPELLYYANK [3+] | 5.11 |  |
|  | RPCFSALTPDETYVPK [3+] | 2.92 |  |
|  | SLHTLFGDELCK [3+] | 2.99 |  |
|  | TVMENFVAFVDK [2+] | 3.82 |  |
|  | VLTSSAR [1+] | 1.83 |  |
|  | VPQVSTPTLVEVSR [2+] | 4.16 |  |
|  | YICDNQDTISSK [2+] | 3.01 |  |
|  | YLYEIAR [2+] | 2.66 |  |
|  | YTRKVPQVSTPTLVEVSR [3+] | 3.42 |  |
| alpha-1-antiproteinase precursor [Bos taurus] | | NP_776307.1 | 8.5 |
|  |  | 2.37 |  |
|  | ADLSGITK [2+] | 2.40 |  |
|  | INDYVEK [2+] | 2.60 |  |
|  | LVDTFLEDVK [2+] | 4.15 |  |
|  | SVLGDVGITEVFSDR [2+] | 4.37 |  |
|  | VLDPNTVFALVNYISFK [2+] | 3.99 |  |
|  | VVNPTQA [1+] | 1.79 |  |
| alpha-2-HS-glycoprotein precursor [Bos taurus] | | NP_776409.1 | 7.9 |
|  | ALGGEDVR [2+] | 2.12 |  |
|  | CDSSPDSAEDVR [2+] | 3.84 |  |
|  | EPACDDPDTEQAALAAVDYINK [2+] | 4.53 |  |
|  | EVVDPTK [1+] | 2.41 |  |
|  | HTLNQIDSVK [2+] | 3.16 |  |
|  | QDGQFSVLFTK [2+] | 2.87 |  |
|  | TPIVGQPSIPGGPVR [2+] | 2.77 |  |
| alpha-2-macroglobulin precursor [Bos taurus] | | NP_001103265.1 | 10.7 |
|  | ATVLNYLPK [2+] | 2.89 |  |
|  | DTGLGLSPTASLR [2+] | 3.62 |  |
|  | DTIIKPLLVEPEGLEK [3+] | 3.38 |  |
|  | GATEITTTITK [2+] | 3.09 |  |
|  | LLIYAILPDGEVVGDSAR [2+] | 4.02 |  |
|  | LPPNVVEESAR [2+] | 3.49 |  |
|  | LSFVTVDSNLR [2+] | 3.96 |  |
|  | SLFTDVVAEK [2+] | 3.05 |  |
|  | SNSFVYLEPLPR [2+] | 2.61 |  |
|  | STGTLLNNAIK [2+] | 3.52 |  |
|  | VSVQLEASPAFLAVPEK [2+] | 4.37 |  |
| annexin A2 [Bos taurus] |  | NP_777141.1 | 7.6 |
|  | DALNIETAIK [2+] | 3.52 |  |
|  | GLGTDEDSLIEIICSR [2+] | 4.36 |  |
|  | GVDEVTIVNILTNR [3+] | 5.18 |  |
|  | QDIAFAYQR [2+] | 2.96 |  |
|  | RAEDGSVIDYELIDQDAR [3+] | 5.76 |  |
|  | SLYYYIQQDTK [2+] | 3.78 |  |
|  | TNQELQEINR [2+] | 4.06 |  |
|  | TPAQYDASELK [2+] | 3.69 |  |
| apolipoprotein A-I preproprotein [Bos taurus] | | NP_776667.2 | 21.7 |
|  | AKPVLEDLR [2+] | 2.23 |  |
|  | DFATVYVEAIK [2+] | 3.64 |  |
|  | DFATVYVEAIKDSGR [2+] | 3.09 |  |
|  | DLEEVK [1+] | 2.22 |  |
|  | DSGRDYVAQFEASALGK [3+] | 2.96 |  |
|  | DYVAQFEASALGK [2+] | 4.39 |  |
|  | EGGGSLAEYHAK [2+] | 3.07 |  |
|  | EQLGPVTQEFWDNLEK [2+] | 4.58 |  |
|  | LLDNWDTLASTLSK [2+] | 2.09 |  |
|  | LSPLAQELR [2+] | 2.71 |  |
|  | PVLEDLR [2+] | 2.27 |  |
|  | QGLLPVLESLK [2+] | 2.67 |  |
|  | QKVQELQDK [2+] | 2.24 |  |
|  | QQLAPYSDDLR [2+] | 2.52 |  |
|  | VAPLGEEFR [2+] | 2.27 |  |
|  | VAPLGEEFREGAR [2+] | 3.40 |  |
|  | VKDFATVYVEAIK [3+] | 3.90 |  |
|  | VKDFATVYVEAIKDSGR [3+] | 4.98 |  |
|  | VQELQDK [2+] | 2.34 |  |
|  | VQPYLDEFQK [2+] | 2.90 |  |
|  | VQPYLDEFQKK [2+] | 2.65 |  |
|  | VREQLGPVTQEFWDNLEK [2+] | 5.76 |  |
|  | VSILAAIDEASK [2+] | 2.09 |  |
|  | VSILAAIDEASKK [3+] | 3.07 |  |
| apolipoprotein A-II precursor [Bos taurus] | | NP_001039381.1 | 3.2 |
|  | AGTDLLNFLSSFIDPK [2+] | 4.18 |  |
|  | AYFEK [1+] | 1.65 |  |
|  | GSELQTQAK [2+] | 3.74 |  |
|  | TQEELTPFFK [2+] | 3.79 |  |
| apolipoprotein B-100 [Bos taurus] | | XP_003582860.1 | 33.8 |
|  | ANWEEGAASELLSSLK [2+] | 4.84 |  |
| ` | AQLSDPTLNPLVLK [2+] | 4.46 |  |
|  | ATGALYDYVNK [2+] | 4.34 |  |
|  | ATSNFPVDLSAFPK [2+] | 3.43 |  |
|  | DPATGQLTGESNLR [2+] | 3.65 |  |
|  | DTVLPVYDK [2+] | 4.12 |  |
|  | EVFNTAR [2+] | 3.93 |  |
|  | EVLLQTFLDDTSPGDKR [2+] | 3.84 |  |
|  | FSVPAGILVPSFGTLTAR [2+] | 2.21 |  |
|  | GFEPTLEALFGK [2+] | 2.09 |  |
|  | ILGEELGFVK [2+] | 2.30 |  |
|  | INFLGQEVSLNVNTENQK [2+] | 3.21 |  |
|  | IPSFQINFK [2+] | 3.88 |  |
|  | ISALVQVR [2+] | 3.18 |  |
|  | ITELSTSAQEVIK [2+] | 3.78 |  |
|  | LATALSLSNR [2+] | 3.76 |  |
|  | LDNIYSSDK [2+] | 3.82 |  |
|  | LEGTSSLTR [2+] | 2.86 |  |
|  | LEVLNFDFQAK [2+] | 2.88 |  |
|  | LLVEDAR [2+] | 2.81 |  |
|  | LNTNIAGLASTVDIR [2+] | 3.08 |  |
|  | LSQQVSDYLSTFNWER [2+] | 3.17 |  |
|  | LVAAASTWLQETSR [2+] | 4.34 |  |
|  | LVTQTEGVK [2+] | 3.82 |  |
|  | NVDASVTTTAR [2+] | 3.67 |  |
|  | QLEALSFNQFLEEVNR [2+] | 2.68 |  |
|  | QQVEAVDVR [2+] | 2.57 |  |
|  | SISLPSLDLVSAK [2+] | 2.46 |  |
|  | SVQLAQQYK [2+] | 3.60 |  |
|  | TEVIPPLIENR [2+] | 4.20 |  |
|  | VAPGEFTITF [1+] | 3.99 |  |
|  | VKENFAGEATLQR [3+] | 2.07 |  |
|  | VLLDQLR [2+] | 2.03 |  |
|  | VPQTDLTFR [2+] | 5.27 |  |
|  | VQIPILK [2+] | 4.79 |  |
|  | VSALLTLAEQTGDWK [2+] | 4.91 |  |
|  | VTEEINAFR [2+] | 5.16 |  |
|  | VVVIGALETVQK [2+] | 4.12 |  |
|  | YENYELTLK [2+] | 2.50 |  |
|  | YNFNSPK [2+] | 4.05 |  |
| complement C3 preproprotein [Bos taurus] | | NP_001035559.2 | 19.5 |
|  | ACEPGVDYVYK [2+] | 3.03 |  |
|  | ADIGCTPGSGR [2+] | 3.76 |  |
|  | AGQYSSDLR [2+] | 2.42 |  |
|  | AQFILQGDACVK [2+] | 2.05 |  |
|  | DICEAQVNSLGR [2+] | 3.76 |  |
|  | DPLTITVR [2+] | 2.28 |  |
|  | EPGQDLVVLPLTITSDFIPSFR [3+] | 2.78 |  |
|  | EVTLEDR [2+] | 2.00 |  |
|  | EYVLPSFEVQLEPEEK [2+] | 4.42 |  |
|  | FLYGEQVDGTAFVIFGVQDGDRR [3+] | 3.28 |  |
|  | FYYIDDPDGLK [2+] | 3.01 |  |
|  | GYTQQLAFR [2+] | 2.85 |  |
|  | IEADQGAR [2+] | 2.18 |  |
|  | ILWESASLLR [2+] | 3.73 |  |
|  | KDYDTTPPVVR [2+] | 3.83 |  |
|  | KGYTQQLAFR [2+] | 2.30 |  |
|  | KIWDVVEK [2+] | 3.00 |  |
|  | LLPVGQTVFITIETPDGIPVK [3+] | 2.86 |  |
|  | LSINTQNK [2+] | 2.53 |  |
|  | LYNVEATSYALLALLAR [2+] | 5.10 |  |
|  | NEQVEIR [2+] | 2.73 |  |
|  | NRWEEPNQK [3+] | 2.77 |  |
|  | NTLIIYLDK [2+] | 2.81 |  |
|  | NYAGVFTDAGLTLK [2+] | 4.12 |  |
|  | RDPLTITVR [2+] | 2.85 |  |
|  | SGSDEVQVK [2+] | 2.99 |  |
|  | SSVAVPYVIVPLK [2+] | 3.54 |  |
|  | TIYTPGSTVLYR [2+] | 3.22 |  |
|  | TSQGLETQQR [2+] | 3.33 |  |
|  | VELLYNPAFCSLATAK [2+] | 4.08 |  |
|  | VFALAANLIAIDSK [2+] | 4.95 |  |
|  | VGLVAVDK [2+] | 2.86 |  |
|  | VPINDGNGEAILK [2+] | 2.62 |  |
|  | VSIRPAPETVK [2+] | 2.92 |  |
|  | VVPEGVR [1+] | 1.77 |  |
|  | VYSYYNLDETCIR [2+] | 3.88 |  |
| complement factor B precursor [Bos taurus] | | NP_001035616.1 | 10.4 |
|  | AGQVLEYLCPSGFYPYPTQIR [3+] | 4.76 |  |
|  | DIEALFVSESK [2+] | 3.85 |  |
|  | DVSEVVTPR [2+] | 3.18 |  |
|  | FLCTGGVDPYADPNTCK [2+] | 4.45 |  |
|  | GIPEFYDYDVALVR [3+] | 3.22 |  |
|  | LQNEDLGFL [1+] | 2.23 |  |
|  | RPQQVPGYAR [2+] | 3.24 |  |
|  | STGSWSTLQTQDR [2+] | 3.43 |  |
|  | VASYGVKPK [2+] | 2.34 |  |
|  | VKDVSEVVTPR [2+] | 4.01 |  |
|  | VTYYCNR [2+] | 2.23 |  |
|  | YGLVTYATEPK [2+] | 4.10 |  |
| fibronectin precursor [Bos taurus] | | NP_001157250.1 | 16.6 |
|  | DLQFVEVTDVK [2+] | 2.07 |  |
|  | EESLPLVGQQSTVSDVPR [2+] | 4.10 |  |
|  | ESVPISDTIIPAVPPPTDLR [3+] | 2.57 |  |
|  | GATYNIIVEAVK [2+] | 3.92 |  |
|  | GDSPASSKPVSINYR [2+] | 3.11 |  |
|  | IGDQWDK [2+] | 2.24 |  |
|  | LGVRPSQGGEAPR [3+] | 4.44 |  |
|  | NSITLTNLNPGTEYVVSIVALNSK [3+] | 4.08 |  |
|  | PAQGVVTTLENVSPPR [3+] | 3.56 |  |
|  | QYNVGPAASQYPLR [2+] | 4.07 |  |
|  | SSPVVIDASTAIDAPSNLR [2+] | 4.83 |  |
|  | STTPDITGYR [2+] | 2.32 |  |
|  | TFYQIGDSWEK [2+] | 2.89 |  |
|  | VTWAPPSSIELTNLLVR [2+] | 3.87 |  |
|  | WLPSSSPVTGYR [2+] | 2.82 |  |
|  | YEVSVYALK [2+] | 2.47 |  |
| gelsolin isoform b [Bos taurus] | | NP_001029799.1 | 10.5 |
|  | AGALNSNDAFVLK [2+] | 4.10 |  |
|  | AQPVQVAEGSEPDSFWEALGGK [3+] | 2.85 |  |
|  | EVQGFESATFLGYFK [2+] | 4.52 |  |
|  | LFACSNK [2+] | 2.31 |  |
|  | PALPAGTEDTAK [2+] | 2.37 |  |
|  | QTQVSVLPEGGETPLFK [2+] | 4.54 |  |
|  | TASDFISK [2+] | 2.36 |  |
|  | TPSAAYLWVGAGASEAEK [2+] | 6.11 |  |
|  | YIETDPANR [2+] | 2.98 |  |
| hemoglobin subunit alpha [Bos taurus] | | NP_001070890.2 | 3.6 |
|  | FLANVSTVLTSK [2+] | 0.40 |  |
|  | LRVDPVNFK [2+] | 0.40 |  |
|  | MFLSFPTTK [2+] | 0.35 |  |
|  | VAAALTK [1+] | 0.31 |  |
|  | VDPVNFK [1+] | 0.33 |  |
| hemoglobin, gamma 2 [Bos taurus] | | NP_001103979.1 | 8.6 |
|  | AAVTSLFAK [2+] | 3.47 |  |
|  | FFESFGDLSSADAILGNPK [2+] | 4.78 |  |
|  | FGSEFSPELQASFQK [2+] | 4.69 |  |
|  | KVLDSFCEGLK [2+] | 3.76 |  |
|  | LLGNVLVVVLAR [2+] | 4.37 |  |
|  | VDEVGGEALGR [2+] | 3.71 |  |
|  | VKVDEVGGEALGR [2+] | 4.28 |  |
|  | VLDSFCEGLK [2+] | 3.42 |  |
| inter-alpha-trypsin inhibitor heavy chain H2 precursor [Bos taurus] | | NP_001091485.1 | 8.6 |
|  | FYNQVSTPLLR [2+] | 3.75 |  |
|  | IIEPQGLR [2+] | 2.10 |  |
|  | IQPSGGTNINEALLR [2+] | 3.64 |  |
|  | IYGNQDTSVQLK [2+] | 3.84 |  |
|  | LWAYLTINQLLAER [2+] | 4.69 |  |
|  | NDLVSATK [2+] | 2.61 |  |
|  | PTVAQQR [2+] | 2.08 |  |
|  | TEDVDQVTVYSYK [2+] | 4.49 |  |
|  | TEVSIAPGAK [2+] | 2.88 |  |
|  | VANTVIQTK [2+] | 2.97 |  |
|  | VQSTITSR [2+] | 2.82 |  |
| peroxiredoxin-1 [Bos taurus] | | 40.19 | 3.6 |
|  | ADEGISFR [2+] | 2.49 |  |
|  | GLFIIDDK [2+] | 2.69 |  |
|  | LVQAFQFTDK [2+] | 3.62 |  |
|  | TIAQDYGVLK [2+] | 2.65 |  |
| pigment epithelium-derived factor precursor [Bos taurus] | | NP_776565.1 | 11.4 |
|  | ALYYDLISNPDIHGTYK [3+] | 2.61 |  |
|  | ASFIPPLEK [2+] | 2.22 |  |
|  | DLLASVTAPQK [1+] | 2.49 |  |
|  | DTDTGALLFIGK [2+] | 4.41 |  |
|  | IAQLPLTGSTSIIFFLPQK [3+] | 6.08 |  |
|  | KTSLEDFYLDEER [2+] | 4.62 |  |
|  | LAAAVSNFGYDLYR [2+] | 4.41 |  |
|  | LQSLFDAPDFSK [2+] | 3.79 |  |
|  | SGESPTANVLLSPLSVATALSALSLGAEQR [2+] | 6.22 |  |
|  | SVQELKLQSLFDAPDFSK [3+] | 2.73 |  |
|  | TSLEDFYLDEER [2+] | 3.32 |  |
|  | TVQAVLTIPK [2+] | 3.68 |  |
|  | VGFEWNEDGAGTNSSPGVQPAR [2+] | 5.48 |  |
|  | YGLDSDLNCK [2+] | 3.87 |  |
| vitronectin precursor [Bos taurus] | | NP_001030222.1 | 7.7 |
|  | DSWVDIFR [2+] | 3.15 |  |
|  | DVWGIEGPIDAAFTR [2+] | 4.27 |  |
|  | FQDGVLEPDFPR [2+] | 3.55 |  |
|  | GLYCYELDEK [2+] | 3.17 |  |
|  | LLFWGGSYGGAGQPQLISR [2+] | 5.71 |  |
|  | NWFGLPGR [2+] | 2.39 |  |
|  | RVDAVIPPYPR [2+] | 2.33 |  |
|  | VDAVIPPYPR [2+] | 2.93 |  |
